# Studies toward Pestalachloride B: Synthesis of the 6/7/6 Tricyclic Scaffold

**DOI:** 10.1021/acs.orglett.5c05259

**Published:** 2026-02-19

**Authors:** Benjamin E. Deprez, Andrew R. LeBlanc, Qimin Winnie Yang, Alexander P. Smith, William M. Wuest

**Affiliations:** Department of Chemistry, Emory University, Atlanta, Georgia 30322, United States

## Abstract

Antibiotic therapy is a critical part of modern healthcare, and the discovery of antibiotics with novel mechanisms of action is needed to combat the rise of antimicrobial resistance. Pestalachloride B, isolated from *Pestalotiopsis adusta*, is a compound belonging to the pestalone family of natural products which demonstrates potent and selective antimicrobial activity against clinically relevant, antimicrobial-resistant Gram-positive bacteria. Here, we report the first synthetic studies focused specifically on pestalachloride B. Two key disconnections were evaluated for the construction of the rare 6/7/6 tricyclic scaffold, and a key intramolecular Parham-type cyclization was found to afford the desired scaffold in 74% yield, enabling us to obtain a late-stage intermediate bearing the complete pestalachloride B framework in 8% overall yield over 11 steps.

## Graphical abstract

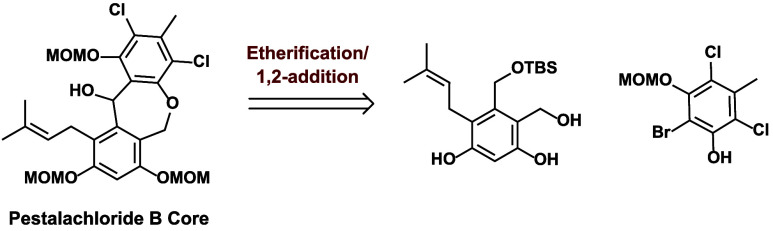

The benzophenone natural product pestalone was first isolated in 2001 from *Pestalotia* strain CNL-365. Since its isolation, more than 20 related benzophenone natural products have been discovered.^1^ Members of the pestalone family of natural products possess a range of antimicrobial and cytotoxic activities and, as a result, have been targets for synthetic chemists over the past decade. The first synthetic effort toward pestalone was reported in 2003 by Kaiser and Schmalz.^2^ Their strategy involved the early joining of two aromatic fragments, followed by decoration of the biaryl core. However, they were unsuccessful in a late-stage formylation and were only able to access des-formyl-pestalone. Less than a year later, Iijima and co-workers successfully synthesized pestalone by joining two predecorated aromatic fragments.^3^ More recently, Arredondo and Slavov have independently published additional syntheses of pestalone and related benzophenone natural products.^4,5^

Despite these synthetic advancements, there are no reported syntheses of pestalachloride B (**1**) or pestalotinones B–C, which all possess an unusual 6/7/6 tricyclic core. In particular, **1** was determined to have potent antibacterial activity with a minimum inhibitory concentration (MIC, μg/mL) of 2.5, 1.25, and 5 against methicillin-susceptible *Staphylococcus aureus* (MSSA), methicillin-resistant *S. aureus* (MRSA), and vancomycin-susceptible *Enterococcus faecium* (VRE), respectively (Figure 1).^6^ In contrast with other members of the pestalone family, **1** was found to have no cytotoxic activity (>50 μM) against a panel of mammalian-derived cell lines.^6^ The selective antibiotic activity coupled with its unique 6/7/6 tricyclic scaffold prompted our group’s investigation of this compound.

**Figure 1:**
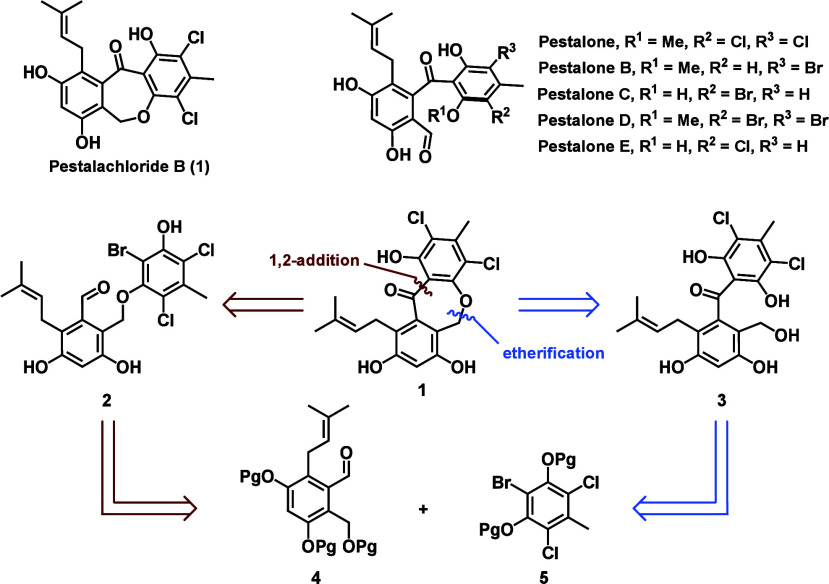
Structures of the pestalone natural products and retrosynthetic analysis of pestalachloride B.

Retrosynthetically, we envisioned two clear disconnections to form the central 7-membered ring: 1) 1,2-addition (Figure 1, red), giving linear ether precursor **2**; and 2) substitution (Figure 1, blue), giving linear benzophenone precursor **3**. Notably, either approach would allow us to begin with the same functionalized fragments (**4** and **5**). Given the precedent set by Kaiser and Schmalz^2^ and Iijima,^3^ we first opted to follow the etherification disconnection to form the 7-membered core.

At the outset, we recognized that protection of the phenolic functionalities would be necessary, and we began by optimizing the choice of protecting group (Figure 2). To prepare the protected western fragments (**12** and **13**), we first performed a dibromination of 3,5-dihydroxybenzoic acid (**6**) to access **7**,^7^ then protected the phenols and carboxylic acid using MOMCl or MEMCl. Both triprotections proceeded in moderate yield. Next, reduction with DIBAL occurred smoothly for the MOM- and MEM-protected intermediates.^8^ Aldehyde fragments **12** and **13** were obtained by DMP oxidation in moderate to high yield.

**Figure 2:**
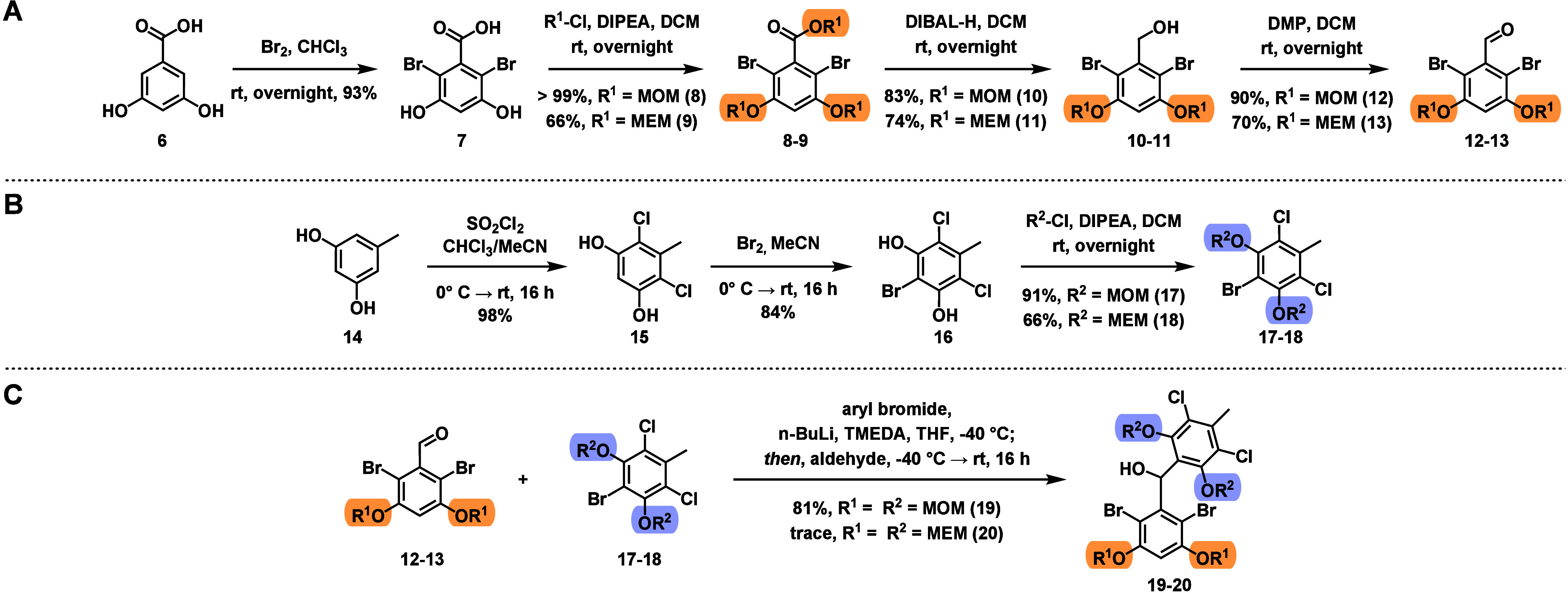
Evaluation of alcohol protecting groups for the synthesis of intermediates **19** and **20**.

To prepare the eastern fragments, orcinol (**14**) was reacted with SO_2_Cl_2_, with excellent selectivity for dichlorination when 2.05 equiv was used. Bromination of the remaining aromatic position with Br_2_ then gave **16**,^2^ and finally, the phenols were protected with either MOMCl or MEMCl to give **17** and **18**. With a series of protected eastern and western aromatic fragments in hand, we set out to see which fragment would be the most optimal in the 1,2-addition reaction. We found that MOM was the most optimal protecting group; when R^1^ = R^2^ = MOM, **19** is obtained in 81% yield. In comparison, when R^1^ = R^2^ = MEM, **20** is only obtained in trace amounts despite complete consumption of **18**, likely due to the increased steric bulk ortho to the reactive site.

Next, oxidation of the diaryl carbinol to the benzophenone proceeded smoothly upon treatment with DMP and NaHCO_3_ to furnish **21** (See Table S1). Decoration of the western ring with the prenyl functionality could be achieved in 78% yield following optimization (confirmed by X-ray crystallography, CCDC 2491749).^5^ We then turned our attention to the installation of the hydroxymethyl group that would be necessary for completion of the 7-membered ether.

Originally, we attempted to perform a Stille coupling between **22** and (tributylstannyl)­methanol, but after extensive screening we were unable to detect any of the desired benzylic alcohol (Table S2).^9,10^ As an alternative, we considered whether we could access a benzylic alcohol through the nucleophilic addition of an organolithium species derived from **22** into the appropriate electrophile. To directly access the hydroxymethylated compound, we envisioned using an electrophile such as formaldehyde,^11^ but neither formaldehyde nor paraformaldehyde reacted as desired. Instead, we screened other one-carbon electrophiles to see whether any could provide an intermediate that could be reduced to the desired benzylic alcohol and found that ethyl formate was competent when a large excess was used (Table S3, confirmed via X-ray crystallography, CCDC 2491791). At this point, we attempted to optimize the reaction by examining different metal sources and additives but were able to observe only a modest improvement to 21% using PhLi (Figure 3, *Inset*, Entry 5; Table S4). Attempts to perform formylation prior to prenylation were explored but were overall unsuccessful (Scheme S1). We hypothesize that the low yields were a result of a sterically hindered environment around the reactive site, with a major contribution from the substituents of the neighboring aromatic moiety (see crystal structure of **22**).

**Figure 3:**
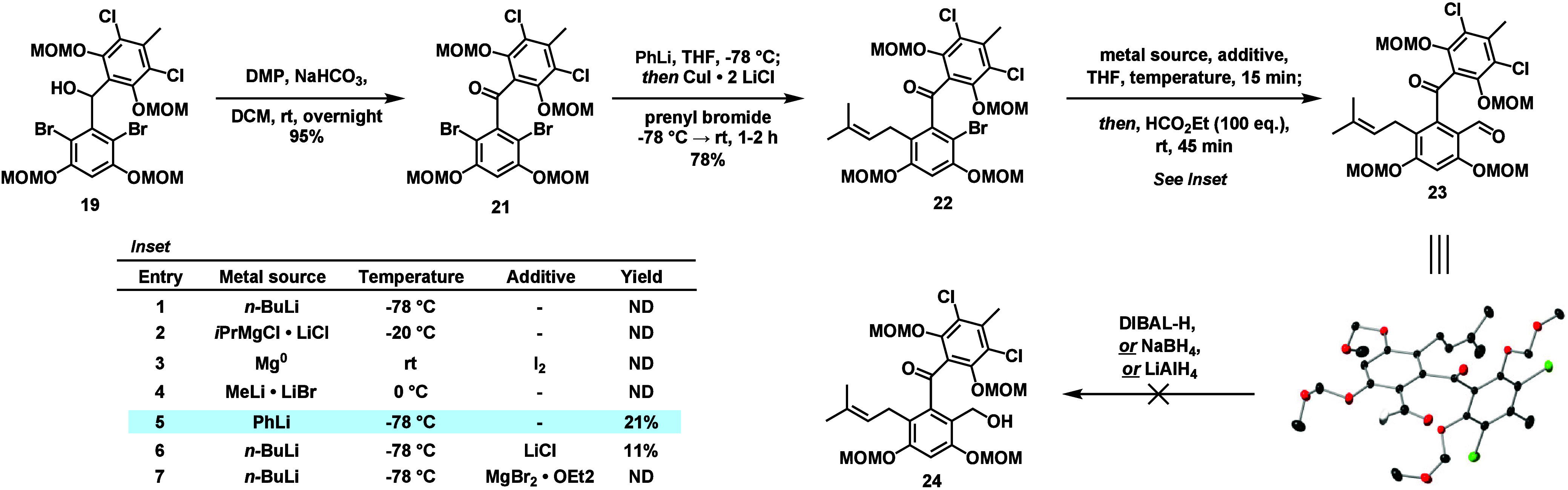
Attempted “benzophenone-first” synthetic route toward pestalachloride B from **20**.

Despite the poor yield of formylated intermediate **23**, we attempted to complete the synthesis as planned, reasoning that the formylation reaction could be further studied if later steps were successful. From **23**, we imagined a one-pot global deprotection and reductive etherification to access pestalachloride B.^12–14^ Unfortunately, most combinations of silane and Brønsted/Lewis acid tested failed to convert **23** to **1**. Separately, we evaluated alternative conditions for the reduction of the aldehyde; however, treatment with various hydride reducing agents returned only starting material or decomposition products.

The challenges associated with installation and reduction of the formyl group suggest that the lithiation approach taken by Kaiser and Schmalz^2^ is inapplicable to the construction of the pestalachloride B core. Therefore, we considered an alternative synthetic strategy based on a different disconnection (Figure 1, red), where the benzyl aryl ether would be formed first, and the cyclization would take place through intramolecular 1,2-addition between the eastern ring and the western aldehyde. We felt that this strategy would be beneficial as the western aromatic fragment could be decorated before joining with the eastern aromatic fragment, which we hoped would simplify the prenylation and formylation reactions and improve the efficiency of the overall route.

To this end, we protected **10** with TBSCl to give **25** in a good yield. Surprisingly, the prenylation reaction, which gave only moderate yields on benzophenone **21**, proceeded in very high yield when it was performed on the TBS-protected alcohol. The subsequent formylation of **26** also gave a higher yield than we observed for other substrates. It is possible that the presence of a primary carbon *ortho* to the reactive sites in these reactions reduces the steric demand compared to **21** or that differences in the electronics or chelating abilities of the silyl ether compared to the benzophenone led to enhanced reactivity of the aryllithium intermediate. Reduction of **27** to the corresponding benzylic alcohol proceeded smoothly with NaBH_4_ (Figure 4A).

**Figure 4:**
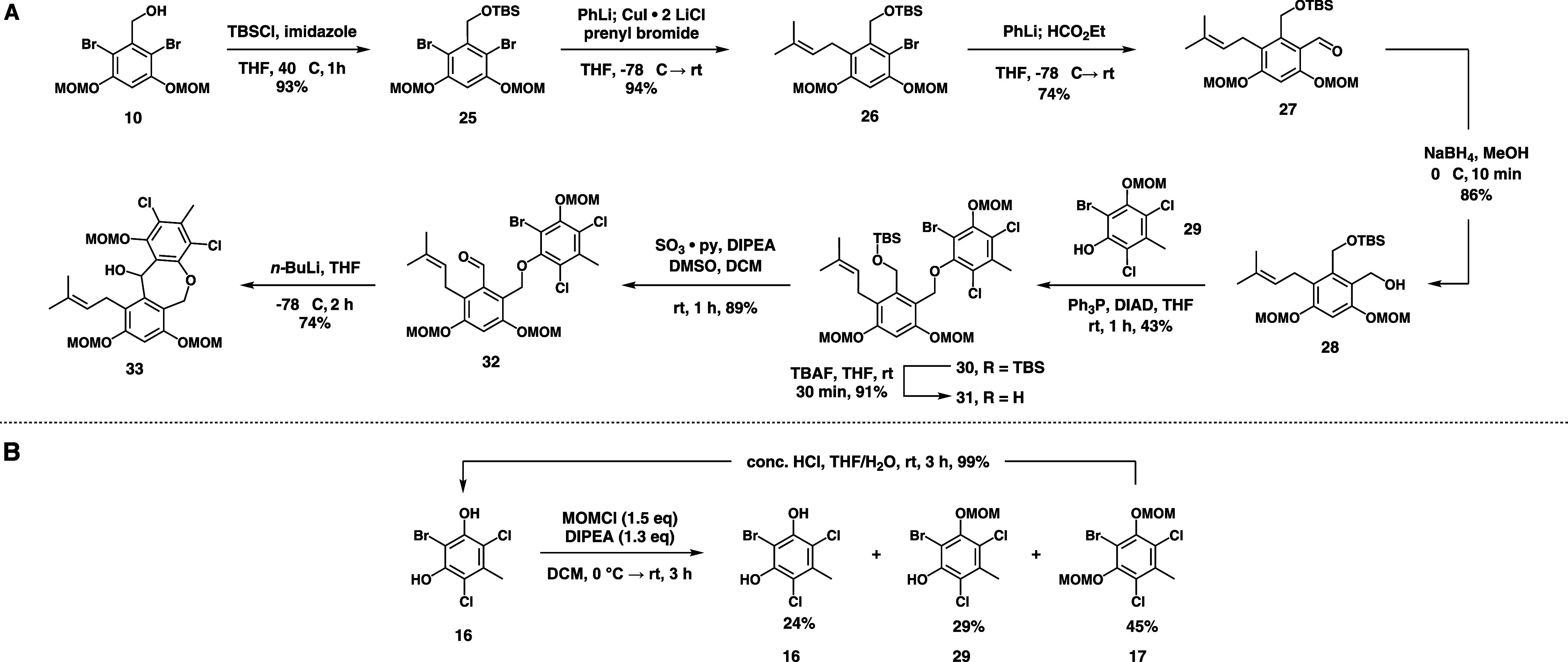
“Ether-first” synthetic route to access **33**.

To assemble linear precursor **30**, we converted **16** to monoprotected eastern fragment **29** (Figure 4B),^2^ which could be coupled to western fragment **28** using the Mitsunobu reaction.^15^ Finally, TBAF deprotection and oxidation of the resulting primary alcohol gave the key aldehyde **32** (Figure 4A). Surprisingly, we were only able to retrieve a single example of an intramolecular reaction between an aryl/alkenyl/alkyl bromide^16^ and an aryl aldehyde to form a 7-membered ring, in addition to a single report of the corresponding addition to simple benzonitriles.^17^ Despite the limited precedent for the desired transformation, simple treatment of **32** with *n*-BuLi in THF produced 74% of the diaroxazepine **33** within 2 h at −78 °C. With the natural product scaffold completed, we faced the endgame of the synthesis requiring peripheral modifications; the cyclic diaryl carbinol would need to be oxidized to the benzophenone and the phenolic MOM groups would have to be removed, although the order for these steps was not obvious.

We first attempted the removal of the MOM protecting group prior to oxidation. Unfortunately, treatment with a wide variety of Lewis acids (including TMOTf/bpy,^18^ NaHSO4/SiO_2_,^19^ Sc­(OTf)_2_, Ce­(OTf)_3_, BF_3_·Et_2_O) all resulted in either partial deprotection or decomposition (Table S5).^20^ We then rationalized that oxidizing **33** to the corresponding benzophenone could allow for a smoother deprotection, as it would limit potential benzylic carbocation formation. We screened over 30 oxidation conditions (Table S6), but unfortunately, none of these conditions resulted in benzophenone formation.

In conclusion, we were able to construct the 6/7/6 tricyclic core of pestalachloride B for the first time. The precedented strategy (i.e., early benzophenone construction)^2,3,5^ toward pestalone-family natural products could not be applied successfully, leading us to a distinct approach in which the ether bond was formed first, followed by intramolecular addition to complete the central 7-membered ring. Of note, 7-exo-trig cyclizations of the type described here are rare in the literature and have not, to our knowledge, been reported on any natural product scaffolds. Although we were ultimately unsuccessful in our efforts to synthesize pestalachloride B, we hope the synthetic discoveries uncovered in the course of this work can be applied to future work on pestalone natural products and provide value to the wider synthetic community.

## Supplementary Material



## Data Availability

The data underlying this study are available in the published article and its Supporting Information.

## Notes

Detailed experimental procedures, characterization data, NMR spectra, and X-ray crystallography reports. Compound **22** corresponds to CCDC accession code 2491749 and compound **23** to CCDC accession code 2491791 (PDF)

## Notes

Present Addresses **Benjamin E. Deprez** – Department of Medical Oncology, Dana-Farber Cancer Institute, Boston, MA, 02115, United States

## Notes

**Winnie Yang** – Department of Chemistry, Yale University, New Haven, CT, 06511, United States
