# Supplementary material for: Studies toward Pestalachloride B: Synthesis of the 6/7/6 Tricyclic Scaffold
Source: Org Lett. 2026 Feb 19;28(9):2831–5. doi: 10.1021/acs.orglett.5c05259 (PMC12973290; doi:10.1021/acs.orglett.5c05259)
Supplement: Supplementary file 1 [file ol5c05259_si_001.pdf]

Supporting Information

Studies toward Pestalachloride B: Synthesis of the 6/7/6 Tricyclic Scaffold

Benjamin E. Deprez, Andrew R. LeBlanc, Qimin Winnie Yang, Alexander P. Smith, William M. Wuest*

Department of Chemistry, Emory University, 1515 Dickey Dr, Atlanta, GA 30322, United States

Table of Contents

List of Abbreviations 2
Optimization of Key Transformations 3
Alternative Route 10
Instrumentation and General Notes 11
Experimental Procedures 11
References 26
Spectral Data 26
X-ray Crystallography Report for **22** 57
X-ray Crystallography Report for **23** 70

Supplementary Figures and Tables

Table S1. Oxidation of **19** 3
Table S2. Attempts to install hydroxymethyl functionality via Stille coupling 4
Table S3. Electrophile screen for formylation of **22** 5
Table S4. Optimization of formylation using HCO_2Et 6
Scheme S1. Formylation of **21**. 6
Table S5. Conditions screen for deprotection of **33** 7
Table S6. Conditions screen for oxidation of **33** 8
Table S7. D incorporation experiment to optimize lithiation reaction 9
Figure S1. ^{1}H NMR Spectra from Table S6 9
Figure S2. Alternative "ether-first" route employing dioxolane protecting group 10

List of Abbreviations

Abbreviation	Long Form
APCI	atmospheric-pressure chemical ionization
aq.	aqueous
DCM	dichloromethane
DI	deionized
DIAD	diisopropyl azodicarboxylate
DIBAL-H	diisobutylaluminum hydride
DIPEA	N,N-diisopropylethylamine
DMP	Dess-Martin periodinane
DMSO	dimethylsulfoxide
ESI	electrospray ionization
hex	hexanes
MEM	methoxyethoxymethyl
MOM	methoxymethyl
NBS	N-bromosuccinimide
NMR	nuclear magnetic resonance
PCC	pyridinium chlorochromate
SEM	2-(trimethylsilyl)ethoxymethyl
TBAF	tetrabutylammonium fluoride
TBATB	tetrabutylammonium tribromide
TFA	trifluoroacetic acid
TLC	thin-layer chromatography
TMEDA	N,N,N,N-tetramethylethylenediamine
py	pyridine

Optimization of Key Transformations

Table S1. Oxidation of **20**

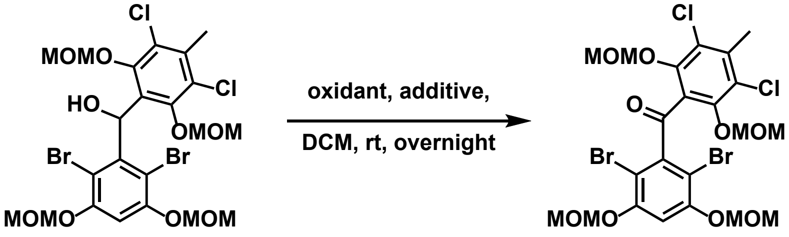

19 **21**

Entry	Oxidant	Additive	Yield
1	DMP	-	80%
2	DMP	$NaHCO_3$	95%
3	PCC	NaOAc	93%
4	PDC	-	40%

Table S2. Attempts to install hydroxymethyl functionality via Stille coupling

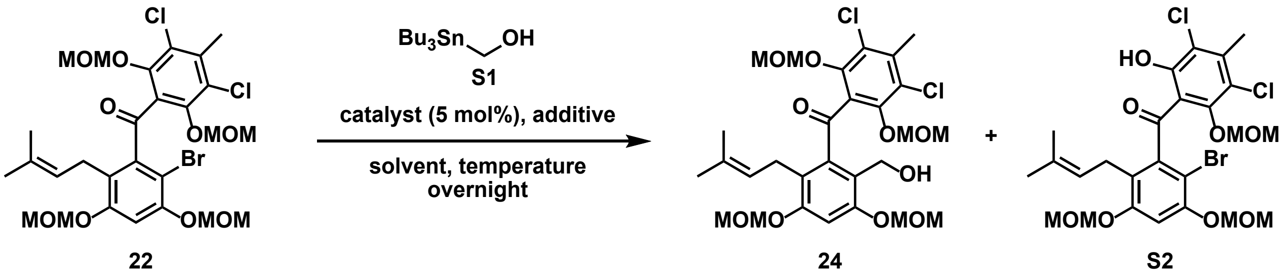

Entry	Catalyst	Solvent	Temperature	Additives	Result
1	$Pd(PPh_3)_4$	dioxane	70 °C	LiCl	S2
2	$Pd(PPh_3)_4$	dioxane	45 °C	LiCl	S2
3	$Pd(PPh_3)_4$	DMF	45 °C	LiCl	22 + S2
4	$Pd(PPh_3)_4$	DMF	45 °C	LiCl + K_2CO_3	S2
7	$Pd(PPh_3)_4$	DMF	45 °C	-	22
5	$Pd(PPh_3)_4$	DMF	45 °C	LiCl + CuI	S2
6	$Pd(PPh_3)_4$	DMF	45 °C	CuI	22 + S2
8	$Pd(PPh_3)_4$	DMF	45 °C	CuI + CsF	22 + S2
9	$Pd(P^tBu_3)_2$	THF	45 °C	LiCl	22
10	$Pd(P^tBu_3)_2$	THF	45 °C	CuI + CsF	22
11	$Pd(P^tBu_3)_2$	DMF	45 °C	CuI + CsF	22 + S2
12	$Pd(PPh_3)_2Cl_2$	dioxane	45 °C	LiCl	S2
13	$Pd(PPh_3)_2Cl_2$	DMF	45 °C	LiCl	S2

Table S3. Electrophile screen for formylation of **22**

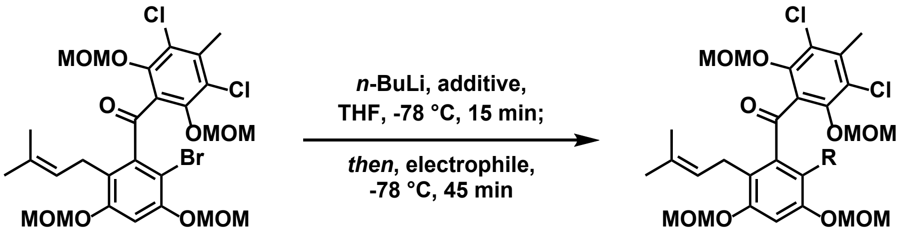

22

R = H, S3
R = CHO, 23

Entry	Electrophile	Additive	Result
1	formaldehyde (g)	-	S3
2	paraformaldehyde	-	S3
3	paraformaldehyde	TMEDA	S3
4	$ClCO_2Et$	$MgBr_2 \cdot OEt_2$	S3
5	DMF	-	S3
6	HCO_2Et	$MgBr_2 \cdot OEt_2$	S3
8	HCO_2Et (65 equiv.)	LiCl	S3 + 23 (12%)

Table S4. Optimization of formylation using HCO_2Et

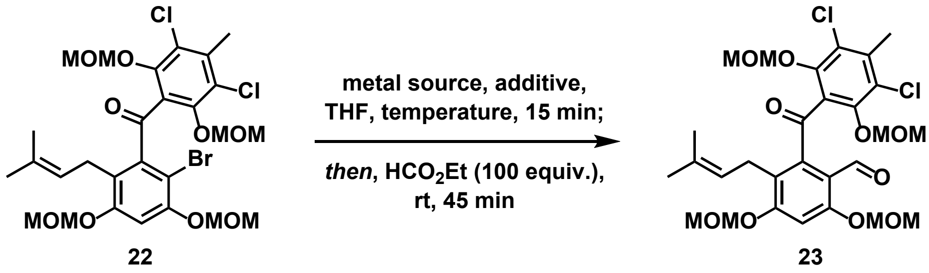

Entry	Metal source	Temperature	Additive	Yield
1	*n*-BuLi	-78 °C	-	ND
2	*i*PrMgCl • LiCl	-20 °C	-	ND
3	Mg^0	rt	I_2	ND
4	MeLi • LiBr	0 °C	-	ND
5	PhLi	-78 °C	-	21%
6	*n*-BuLi	-78 °C	LiCl	11%
7	*n*-BuLi	-78 °C	$MgBr_2$ • OEt_2	ND
8	*n*-BuLi	-78 °C	TMEDA	ND
9	PhLi	-78 °C	LiCl	ND
10	PhLi	-78 °C	CuI • 2 LiCl	ND
11	PhLi	-78 °C	$MgBr_2$ • OEt_2	1%

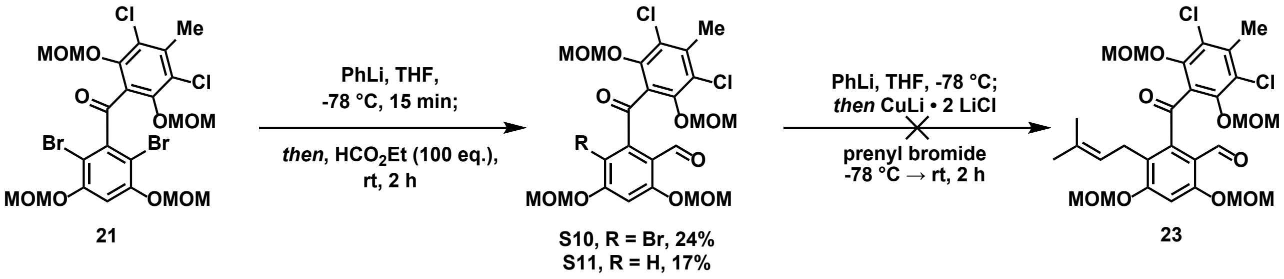

Scheme S1. Formylation of **21**.

Table S5. Conditions screen for deprotection of **33**

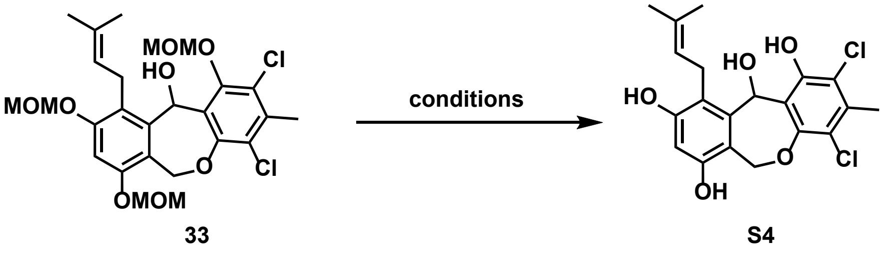

Entry	Lewis Acid	Solvent	Result
1	TMSOTf/bpy	MeCN	NR
2	$NaHSO_4$	DCM	NR
3	$Sc(OTf)_3$	$MeNO_2$	NR
4	$Ce(OTf)_3$	$MeNO_2$	NR
5	TMSCl/TBAB	DCM	NR
6	BF_3•OEt_2	DCM	NR

Table S6. Conditions screen for oxidation of **33**

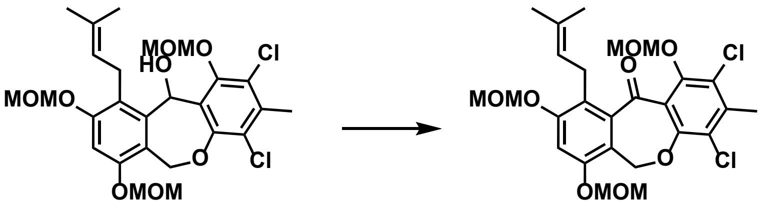

Entry	Oxidant	Additive	Solvent	Result
1	DMP	-	THF	NR
2	DMP	TEA	DCM	NR
3	DMP	-	DCM	NR
4	DMP	2,6-Lut.	DCM	NR
5	PCC	-	DCM	NR
6	PCC	40°C	DCM	NR
7	PDC	-	DCM	NR
8	PDC	40°C	DCM	NR
9	Oxone	$NaHCO_3$	MeOH	NR
10	MnO_2	-	DCM	NR
11	Oxone, TEMPO	NBu_4Br	DCM	NR
12	Oxone, TEMPO	NaBr	DCM	Decomp.
13	$Fe(NO_3)_2$	-	DCM	NR
14	$Fe(NO_3)_2$, ABNO	-	DCM	NR
15	TEMPO, NaOCl	KBr	DCM	NR
16	NIS, DMS, TEA	-	DCM	NR
17	Bobbit's Salt	-	DCM	Decomp.

Table S7. D incorporation experiment to optimize lithiation reaction

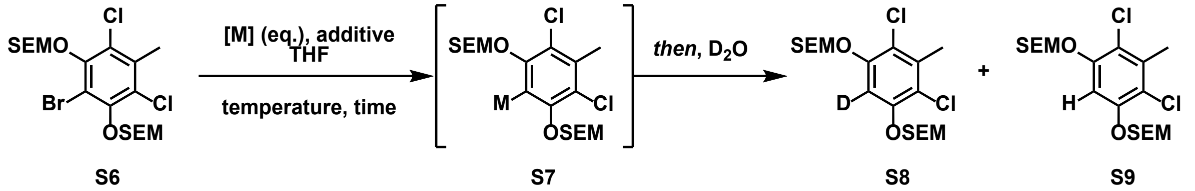

Entry	[M] (equiv.)	Additive	Lithiation time	Concentration	Temperature	% Conv.	% D incorp.
1	*n*-BuLi (1.5 equiv.)	TMEDA (1.8 equiv.)	1 h	0.1 M	-78 °C	100	44
2	*n*-BuLi (1.5 equiv.)	TMEDA (1.8 equiv.)	30 min	0.1 M	-78 °C	98	83
3	*n*-BuLi (1.5 equiv.)	TMEDA (1.8 equiv.)	10 min	0.1 M	-78 °C	99	99
4	*i*PrMgCl • LiCl (1.5 equiv.)	-	1 h	0.1 M	-20 °C	52	78
5	*i*PrMgCl • LiCl (1.5 equiv.)	-	1 h	0.1 M	-20 °C → rt	99	99

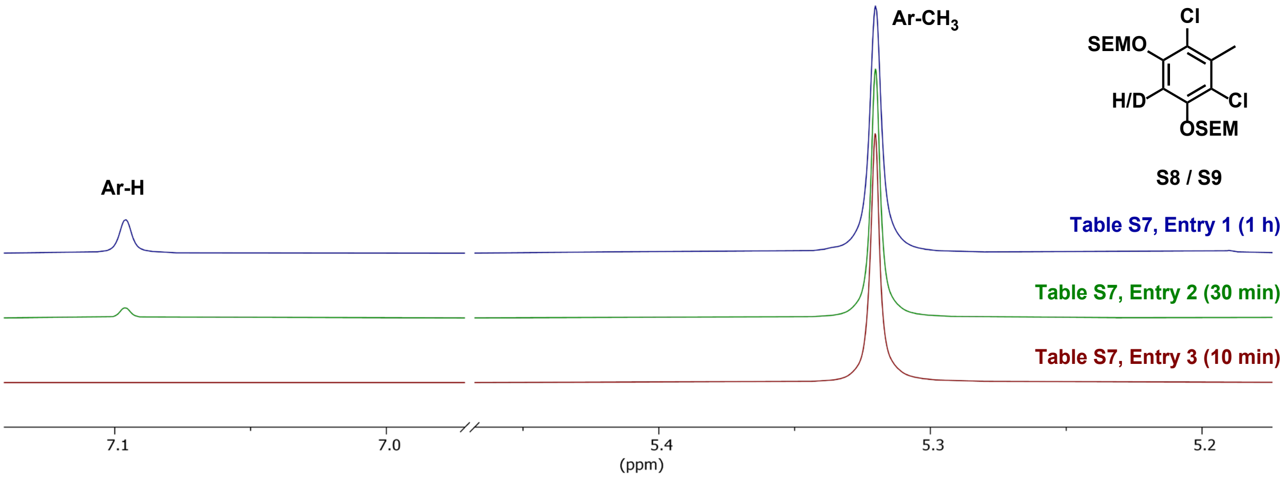

Figure S1. ^{1}H NMR Spectra from Table S6

Alternative Route

Originally, we attempted to mask the aldehyde functionality of **12** as the corresponding dioxolane, as an alternative to the TBS-protected benzylic alcohol shown in Scheme 1 of the main text. One interesting finding from this exploration (Fig S2 A) was the sensitivity of the reduced benzylic alcohol intermediate **S15** to aqueous conditions, presumably due to formation of transacetal **S17** as demonstrated in Fig. S2 B. While this could be circumvented through the use of Na_2SO_4 • 10 H_2O to work up the reduction reaction, selective deprotection of the dioxolane proved challenging, and this route was abandoned in favor of the TBS-protected alcohol route.

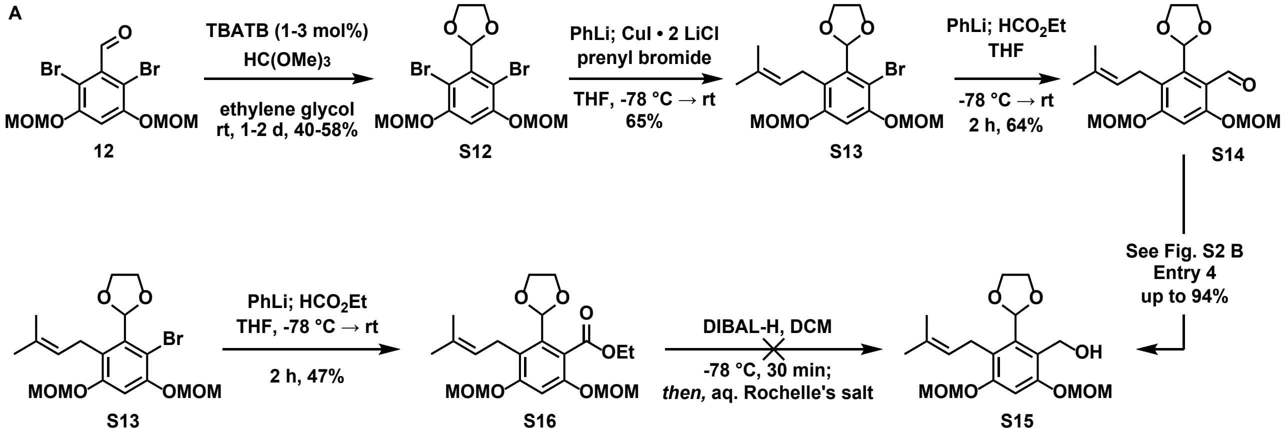

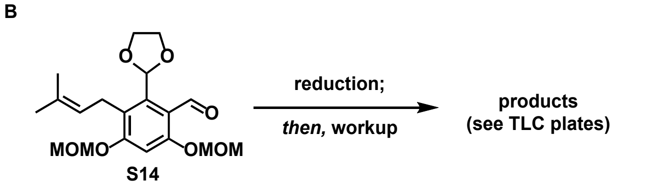

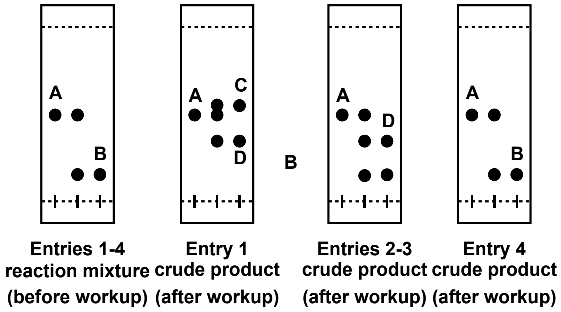

Entry	Reducing agent/solvent	Workup procedure
1	DIBAL-H/DCM	aq. Rochelle's salt, overnight, LLE
2	$LiAlH_4$/THF	Na_2SO_4 • 10 H_2O; H_2O, LLE
3	$NaBH_4$/MeOH	H_2O, LLE
4	$LiAlH_4$/THF	Na_2SO_4 • 10 H_2O, filtration

LLE = liquid-liquid extraction with EtOAc

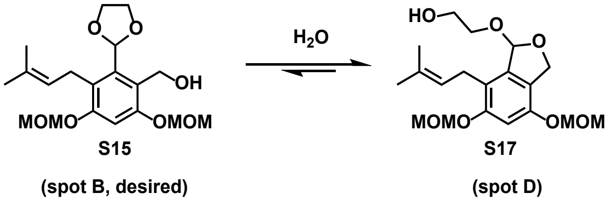

Figure S2. Alternative "ether-first" route employing dioxolane protecting group

Instrumentation and General Notes

Non-aqueous reactions were carried out under an atmosphere of argon in flame-dried glassware. Tetrahydrofuran (THF), dichloromethane (DCM), diethyl ether (Et_2O), toluene, and N,N-dimethylformamide (DMF) were dried by passage through alumina. Triethylamine was freshly distilled from CaH_2 prior to use. All other commercial reagents and anhydrous solvents were used as received (from Sigma Aldrich, Oakwood Chemical, Combi Blocks, Alfa Aesar, Fisher Scientific, TCI America, or AK Scientific) without further purification. Brine refers to a saturated aqueous solution of sodium chloride. Reactions were monitored by analytical thin layer chromatography (TLC) using EMD Millipore silica gel 60 F_{254} precoated plates and visualized using UV and/or ninhydrin, vanillin, or $KMnO_4$ stains. Flash chromatography employing Siliaflash silica gel (40-63 µm) was performed on a Biotage Isolera One instrument with a linear normal-phase gradient.

Nuclear magnetic resonance (^1H, ^{13}C NMR) spectra were recorded using the following spectrometers: Bruker Avance NEO800 (800 MHz), Varian INOVA600 (600 MHz), Bruker Ascend (600 MHz), Varian INOVA500 (500 MHz), Varian INOVA400 (400 MHz), Bruker Avance NEO400 (400 MHz), and Bruker Avance III (400 MHz). Chemical shifts are reported in parts per million (ppm) relative to tetramethylsilane and referenced to the residual solvent signal. Signal patterns are indicated as follows: s (singlet), d (doublet), t (triplet), q (quartet), m (multiplet), br (broad signal). Structural assignments were made with additional information from gCOSY, gHSQC, and gHMBC experiments. High-resolution mass spectra were measured using a Thermo Q Exactive Orbitrap mass analyzer using either APCI or ESI techniques.

Experimental Procedures

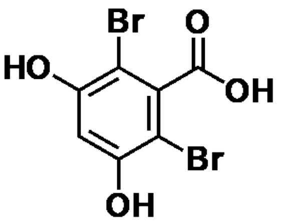

2,6-dibromo-3,5-dihydroxybenzoic acid (7): To a suspension of 3,5-dihydroxybenzoic acid (12.0 g, 1 equivequiv, 77.9 mmol) in $CHCl_3$ (160 mL) was added a solution of bromine (26.1 g, 8.42 mL, 2.1 equivequiv, 164 mmol) in $CHCl_3$ (40 mL) at room temperature over 30 min from an addition funnel. After the addition of bromine, the mixture was stirred at room temperature for 8 h. Air was blown over the mixture and the exhaust were bubbled through aq. NaOH/Na_2SO_4 solution, then the precipitate was filtered and

the residue was washed with cold H2O (3 x 30 mL) and CHCl3 (3 x 60 mL). The resulting white solid was dried overnight in a vacuum desiccator to give the title compound (22.5 g, 72.1 mmol, 93 %) as a white powder. ^{1}H NMR (400 MHz, DMSO-d_6) δ 13.64 (s, 1H), 10.56 (s, 2H), 6.68 (s, 1H). ^{13}C NMR (101 MHz, DMSO-d_6) δ 167.08, 154.28, 140.00, 103.17, 95.34. NMR data for **7** are consistent with literature report.[1]

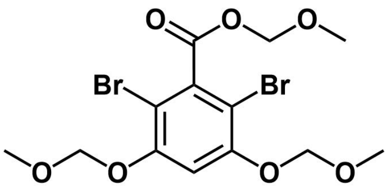

methoxymethyl 2,6-dibromo-3,5-bis(methoxymethoxy)benzoate (8): MOMCl was prepared in situ: A 3-neck round bottom flask was fitted with an addition funnel and charged with zinc(II) bromide (48.87 mg, 0.1 mol%, 217 µmol), then flame dried under vacuum and placed under Ar atmosphere. Dimethoxymethane (19.2 mL, 1 equivequiv, 217 mmol) and DCM (110 mL) were added, then oxalyl chloride (9.497 mL, 0.5 equivequiv, 109 mmol) was added over a 5 min period. When the addition was complete, the reaction began to warm, then cool to room temperature over a period of ~ 1 h.

To the solution of MOMCl (4.0 equiv, 217 mmol), **7** (16.88 g, 1 equiv, 54.2 mmol) was added and the mixture was cooled to 0 °C. DIPEA (47.1 mL, 5.0 equiv, 271 mmol) was added over a 30 min period, then the cooling bath was removed, and the mixture was stirred overnight. The reaction was quenched with sat. aq. NH_4Cl and the aqueous layer was extracted with DCM. The combined organic layers were washed with brine, dried over Na_2SO_4, and concentrated. The residue was purified by flash column chromatography (2 x 100 g, 0-40% EtOAc/hex) to afford the title compound (24.02 g, 54.1 mmol, > 99 %) as a white solid. ^{1}H NMR (400 MHz, DMSO-d_6) δ 7.27 (d, J = 2.4 Hz, 2H), 6.96 (t, J = 2.3 Hz, 1H), 5.43 (s, 2H), 5.24 (s, 4H), 3.45 (s, 3H), 3.39 (s, 6H). ^{13}C NMR (101 MHz, DMSO-d_6) δ 164.8, 157.9, 131.5, 110.0, 109.8, 94.1, 90.8, 57.2, 55.8. HRMS (APCI+): calcd for $C_{13}H_{16}O_7{}^{79}Br_2$ 441.9257, found 441.9264.

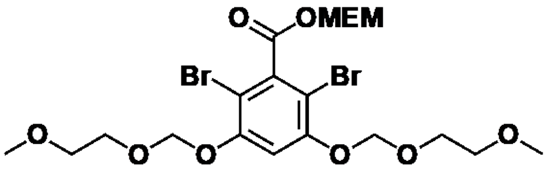

(2-methoxyethoxy)methyl 2,6-dibromo-3,5-bis((2-methoxyethoxy)methoxy)benzoate (9): A DCM (8.0 mL) suspension of **7** (500 mg, 1 equiv, 1.60 mmol) was treated with DIPEA (1.68 mL, 6 equiv, 9.62 mmol). The resulting white suspension was cooled to 0 °C and MEMCl (824 µL, 4.5 equiv, 7.21 mmol) was added over 1 h via addition funnel. The reaction was allowed to slowly warm to room temperature while stirring

overnight. The resulting brown solution was partitioned with $NaHCO_3$ and the aqueous layer was extracted with DCM. The combined organic layers were washed with brine, dried over Na_2SO_4, and purified by flash chromatography (20-80% EtOAc/Hex) to afford the title compound (611 mg, 1.06 mmol, 66 %) as a white solid. ^{1}H NMR (400 MHz, DMSO-d_6) δ 7.23 (s, 1H), 5.53 (s, 2H), 5.40 (s, 4H), 3.88 – 3.80 (m, 2H), 3.80 – 3.72 (m, 4H), 3.52 – 3.46 (m, 6H), 3.25 (s, 3H), 3.22 (s, 6H). ^{13}C NMR (101 MHz, DMSO-d_6) δ 164.9, 153.7, 138.3, 105.4, 101.2, 94.2, 91.1, 70.9, 70.8, 69.5, 68.0, 58.1, 58.1.HRMS (APCI+): calcd for $C_{19}H_{28}O_{10}{}^{79}Br_2$ 574.0044, found 574.0049.

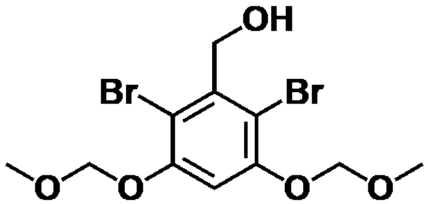

(2,6-dibromo-3,5-bis(methoxymethoxy)phenyl)methanol (10): A solution of **8** (5.00 g, 95 wt%, 1 equiv, 10.7 mmol) in DCM (43 mL) was cooled to -78 °C and DIBAL-H (32 mL, 1.0 molar in DCM, 3 equiv, 32 mmol) was added dropwise. The reaction was stirred for 6 h at -78 °C, at which point TLC indicated complete reaction. The mixture was quenched carefully with MeOH, then warmed to room temperature and treated with aq. Rochelle's salt. The biphasic mixture was stirred for 3 h, then the aqueous layer was extracted with DCM and the combined organic layers were washed with brine, dried over Na_2SO_4, and concentrated to afford the title compound as a white solid without the need for purification (3.48 g, 9.01 mmol, 83 %). ^{1}H NMR (400 MHz, $CDCl_3$) δ 7.03 (s, 1H), 5.23 (s, 4H), 5.07 (d, J = 7.2 Hz, 2H), 3.51 (s, 6H), 2.29 (t, J = 7.2 Hz, 1H). ^{13}C NMR (101 MHz, $CDCl_3$) δ 154.0, 140.1, 108.7, 104.3, 95.6, 65.9, 56.7. HRMS (APCI+): calcd for $C_{11}H_{14}O_5{}^{79}Br_2$ $[M]^+$ 383.9203, found 383.9199.

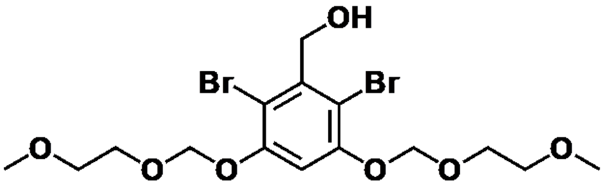

(2,6-dibromo-3,5-bis((2-methoxyethoxy)methoxy)phenyl)methanol (11): A solution of **9** (544 mg, 1 equiv, 944 μmol) in DCM (4.7 mL) was cooled to -78 °C and DIBAL-H (4.72 mL, 1.0 molar in DCM, 5 equiv, 4.72 mmol) was added dropwise. The reaction was stirred for 90 min at -78 °C, at which point TLC indicated complete reaction. The mixture was quenched carefully with MeOH, then warmed to room temperature and treated with aq. Rochelle's salt. The biphasic mixture was stirred for 3 h, then the aqueous layer was extracted with DCM and the combined organic layers were washed with brine, dried over Na_2SO_4, and concentrated. The crude was purified by flash chromatography (EtOAc/hex) to afford the title compound (331 mg, 698 μmol, 74 %) as a white solid. ^{1}H NMR (400 MHz, DMSO-d_6) δ 7.12 (s, 1H), 5.35 (s, 4H), 5.13 (t, J = 5.3 Hz, 1H), 4.80 (d, J = 5.3 Hz, 2H), 3.78 – 3.75 (m,

4H), 3.50 – 3.45 (m, 4H), 3.23 (s, 6H). ^{13}C NMR (101 MHz, $CDCl_3$) δ 153.9, 140.0, 108.9, 104.8, 94.5, 71.5, 68.3, 65.8, 59.1. HRMS (ESI+): calcd for $C_{15}H_{22}O_7{}^{79}Br_2{}^{23}Na$ [M+Na]$^+$ 494.9625, found 468. 494.9624.

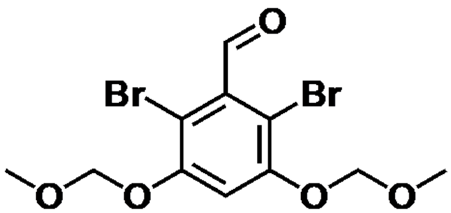

2,6-dibromo-3,5-bis(methoxymethoxy)benzaldehyde (12): A DCM (20 mL) solution of **10** (4.874 g, 1 equiv, 12.63 mmol) was added to a stirred suspension of PCC (8.98 g, 3.3 equiv, 41.7 mmol) and sodium acetate (830 mg, 0.8 equiv, 10.1 mmol) in DCM (60 mL) at rt. The reaction mixture quickly turned from orange-red to brown and the reaction was allowed to continue for 3 h before a second loading of PCC (2.72 g, 1 equiv, 12.6 mmol) was added. The reaction was stirred an additional 3 h at which point complete consumption of starting material was observed by TLC. The mixture was diluted with Et_2O and the resulting suspension was stirred with celite for 20 min, then filtered over a pad of celite. The filtrate was concentrated and purified by flash chromatography to afford the title compound (4.36 g, 11.4 mmol, 90 %) as a white solid. ^1H NMR (400 MHz, DMSO-d_6) δ 10.08 (s, 1H), 7.27 (s, 1H), 5.36 (s, 5H), 3.42 (s, 7H). ^{13}C NMR (101 MHz, DMSO-d_6) δ 192.8, 153.7, 136.6, 106.8, 104.4, 95.3, 56.4. HRMS (APCI+): calcd for $C_{11}H_{13}O_5{}^{79}Br_2$ [M+H]$^+$ 382.9124, found 382.9121.

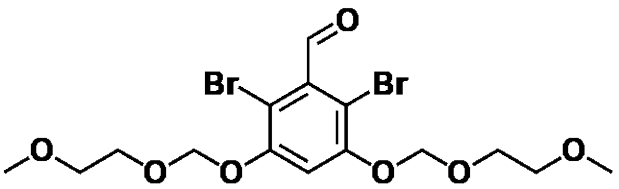

2,6-dibromo-3,5-bis((2-methoxyethoxy)methoxy)benzaldehyde (13): A DCM (10 mL) solution of **11** (331 mg, 1 equiv, 698 μmol) was cooled to 0 °C. DMP (1.18 g, 4 equiv, 2.79 mmol) was added in one portion and the mixture was stirred for 1 h before the ice bath was removed. The mixture was stirred for overnight, then DCM was added and insoluble material was removed by vacuum filtration through sand/celite. The filtrate was concentrated to and the residue was purified by flash chromatography (20-80% EtOAc/hex) to afford the title compound (197 mg, 417 μmol, 70 %) as a white solid. ^1H NMR (400 MHz, DMSO-d_6) δ 10.08 (s, 1H), 7.33 (s, 1H), 5.41 (s, 4H), 3.80 – 3.69 (m, 4H), 3.51 – 3.44 (m, 4H), 3.22 (s, 6H). ^{13}C NMR (101 MHz, $CDCl_3$) δ 191.6, 154.2, 135.8, 107.9, 106.7, 94.6, 71.5, 68.5, 59.2. HRMS (ESI+): calcd for $C_{15}H_{20}O_7{}^{79}Br_2{}^{23}Na$ [M+Na]$^+$ 492.947, found 492.946.

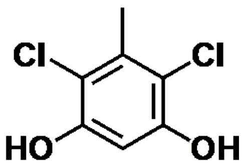

4,6-dichloro-5-methylbenzene-1,3-diol (15): Sulfuryl chloride (22.3 g, 13.4 mL, 2.05 equiv, 165 mmol, solution in 30 mL $CHCl_3$) was added over 30 min to a 0 °C solution of orcinol (**14**, 10.0 g, 1 equiv, 80.6 mmol) in $CHCl_3$ (170 mL) and MeCN (34 mL). After the addition was complete, the reaction mixture was warmed to room temperature and stirred for 16 h, after which TLC confirmed consumption of starting material. 10% NaOH solution (20 mL) was added to quench excess sulfuryl chloride and the mixture was stirred 30 min before adjusting to pH ~4. The mixture was partitioned with additional water and the aqueous layer was extracted with DCM. The combined organics were washed with brine, dried over Na_2SO_4, and concentrated in vacuo. The crude product was recrystallized from ~800 mL of hot 15:1 CyH/EtOAc (10.43 g, 54.04 mmol, 67 %, white needles), and the mother liquor was concentrated and redissolved in ~300 mL of the same solvent mixture to afford a second crop of crystals (2.45 g, 12.7 mmol, 15.8 %, white needles). The remaining mother liquor was concentrated and purified by flash chromatography (100 g; 0-30% EtOAc/hex) to afford a final batch of product (300 mg, 1.55 mmol, 1.93 %, white powder). In total 13.18 g pure **15** was isolated (68.28 mmol, 85 %). ^1H NMR (400 MHz, DMSO-d_6) δ 10.07 (s, 2H), 6.56 (s, 1H), 2.33 (s, 3H). ^{13}C NMR (101 MHz, DMSO-d_6) δ 152.1, 134.4, 111.1, 101.6, 17.7. NMR data for **12** match literature report.[2]

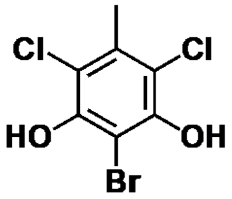

2-bromo-4,6-dichloro-5-methylbenzene-1,3-diol (16) :A solution of **15** (11.00 g, 1 equiv, 56.99 mmol) in MeCN (190.0 mL) was cooled to 0 °C. A solution of bromine (13.66 g, 4.404 mL, 1.5 equiv, 85.48 mmol) in 15 mL MeCN was added dropwise over a 15 min period, turning the reaction mixture dark red, and the ice bath was removed. The reaction appeared complete by TLC after 4 h at rt. Sat. aq. $Na_2S_2O_3$ was added to quench excess Br_2 and a faintly green suspension formed. EtOAc and DI water were added and the layers were partitioned. The aqueous layer was extracted with EtOAc and the combined organic layers were washed with brine, dried over Na_2SO_4, and concentrated. The solid residue was recrystallized from hot CyH, affording a first crop of fine, cotton-like white needles (9.00 g, 33.1 mmol, 58.1 %). The mother liquor was concentrated and heated to redissolve precipitate. Slow cooling of this solution afforded an additional 2.25 g (8.27 mmol, 14.5 %, fine white needles), and column chromatography of the concentrated mother liquor (50 g, 0-50% EtOAc/hex) gave 1.87 g (6.88 mmol, 12.1 %) of white solid. In total 13.12 g of **16** was isolated (48.25 mmol, 85 %). ^1H NMR (400 MHz, DMSO-d_6) δ 9.91 (s, 2H), 2.35

(d, J = 1.8 Hz, 3H). ^{13}C NMR (101 MHz, DMSO-d_6) δ 149.4, 133.0, 113.5, 100.1, 18.2. NMR data for **16** match literature report.[2]

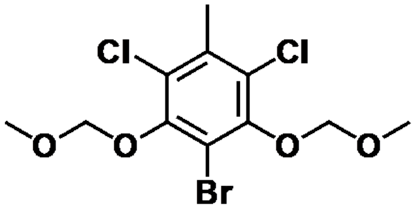

1-bromo-3,5-dichloro-2,6-bis(methoxymethoxy)-4-methylbenzene (17): MOMCl was prepared *in situ*: A 3-neck round bottom flask was fitted with an addition funnel and charged with zinc(II) bromide (23 mg, 0.1 mol%, 100 μmol), then flame dried under vacuum and placed under Ar atmosphere. dimethoxymethane (9.1 mL, 1 equiv, 102 mmol) and DCM (50 mL) were added, then oxalyl chloride (4.48 mL, 0.5 equiv, 51.2 mmol) was added over a 5 min period. When the addition was complete, the reaction began to warm, then cool to room temperature over a period of ~ 1 h.

To the solution of MOMCl (3.1 equiv with respect to substrate, 102 mmol), **16** (8.97 g, 1 equiv, 33.0 mmol) was added and the mixture was cooled to 0 °C. DIPEA (17.05 g, 23.0 mL, 4 equiv, 132 mmol) was added over a 30 min period, then the mixture was stirred at 0 °C for 3 h. The reaction was quenched with sat. aq. NH_4Cl and the aqueous layer was extracted with DCM. The combined organic layers were washed with brine, dried over Na_2SO_4, and concentrated. The residue was purified by flash chromatography (200 g, 0-40% EtOAc/hex) to afford the title compound (10.84 g, 30.1 mmol, 91 %) as a white solid. ^1H NMR (400 MHz, DMSO-d_6) δ 5.14 (d, J = 2.2 Hz, 4H), 3.59 (s, 6H), 2.44 (d, J = 2.2 Hz, 3H). ^{13}C NMR (101 MHz, DMSO-d_6) δ 150.3, 135.5, 125.6, 113.0, 99.9, 58.4, 18.9. NMR data for **14** match literature report.[3]

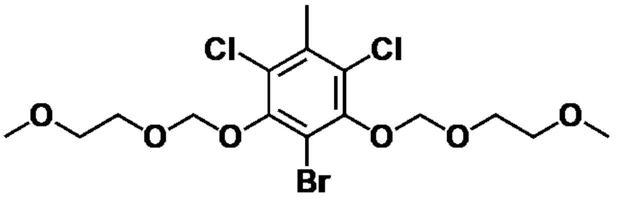

1-bromo-3,5-dichloro-2,6-bis((2-methoxyethoxy)methoxy)-4-methylbenzene (18): A DCM (3.7 mL) suspension of **16** (200 mg, 1 equiv, 736 μmol) was treated with DIPEA (512 μL, 4 equiv, 2.94 mmol). The resulting white suspension was cooled to 0 °C and MEMCl (210 μL, 2.5 equiv, 1.84 mmol) was added dropwise by syringe. The reaction was allowed to slowly warm to room temperature while stirring overnight. The reaction mixture was quenched with NH_4Cl, extracted with EtOAc, washed with brine and dried over Na_2SO_4. The crude material was purified by flash chromatography to afford the title compound (236 mg, 527 μmol, 71.6 %) as a white solid. ^1H NMR (400 MHz, DMSO-d_6) δ 5.20 (s, 4H), 4.02 – 3.85 (m, 4H), 3.54 – 3.46 (m, 4H), 3.25 (s, 6H), 2.44 (s, 3H). ^{13}C

NMR (101 MHz, DMSO-d_6) δ 149.6, 135.0, 125.2, 112.6, 98.0, 71.0, 69.3, 58.1, 18.3. HRMS (ESI+): calcd for $C_{15}H_{21}O_6{}^{79}Br^{35}Cl_2{}^{23}Na$ [M+Na]$^+$ 468.979, found 468.9785.

(((((2-bromo-4,6-dichloro-5-methyl-1,3-phenylene)bis(oxy))bis(methylene))bis(oxy))bis(ethane-2,1-diyl))bis(trimethylsilane)

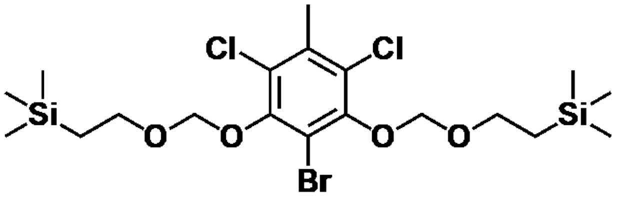

(S6): A DCM (3.68 mL) suspension of **16** (500 mg, 1 equiv, 1.84 mmol) was treated with DIPEA (951 mg, 1.28 mL, 4 equiv, 7.36 mmol). The resulting white suspension was cooled to 0 °C and SEMCl (766 mg, 814 µL, 2.5 equiv, 4.60 mmol) was added dropwise by syringe. The reaction was allowed to slowly warm to room temperature while stirring overnight. Sat. aq. NH_4Cl was added and the aqueous layer was extracted with EtOAc. The combined organic layers were washed with brine, dried over Na_2SO_4, and concentrated. The residue was purified by flash chromatography (50 g; 0-10% Et_2O/hexane) to afford the title compound (770 mg, 1.45 mmol, 79%) as a colorless oil. ^1H NMR (400 MHz, DMSO-d_6) δ 5.18 (s, 4H), 3.97 – 3.87 (m, 4H), 2.44 (s, 3H), 1.00 – 0.89 (m, 4H), 0.02 (s, 18H ^{13}C NMR (101 MHz, DMSO-d_6) δ 149.8, 135.1, 125.1, 112.6, 97.4, 67.5, 18.4, 17.7, -1.3, -1.3, -1.3. HRMS (ESI–): calcd for $C_{19}H_{33}O_4{}^{79}Br^{35}Cl_3{}^{28}Si_2$ [M+Cl]$^-$ 565.0172, found 565.0168.

(((((4,6-dichloro-5-methyl-1,3-phenylene)bis(oxy))bis(methylene))bis(oxy))bis(ethane-2,1-diyl))bis(trimethylsilane) (S9):

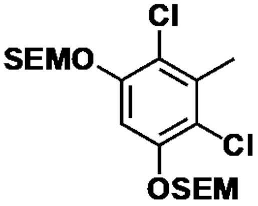

An analytical sample of was prepared to facilitate analysis of the crude mixtures. A THF solution (0.5 mL) of **S5** (25 mg, 1 equiv, 47 µmol) and TMEDA (9.8 mg, 13 µL, 1.8 equiv, 85 µmol). was cooled to -40 °C and n-butyllithium (4.5 mg, 39 µL, 1.8 molar, 1.5 equiv, 70 µmol) was added dropwise, then the reaction was stirred at the same temperature for 1 h. The cooling bath was removed and the reaction was quenched with water. After 10 min, the mixture was acidified with 1 N HCl and the aqueous layer was extracted with EtOAc. The combined organic layers were washed with brine, dried over Na_2SO_4, and concentrated to afford the title compound as an amorphous yellow solid (yield not determined). ^1H NMR (400 MHz, DMSO-d_6) δ 7.09 (s, 1H), 5.31 (s, 4H), 3.82 – 3.61 (m, 4H), 2.42 (s, 3H), 0.95 – 0.81 (m, 4H), -0.03 (s, 18H). ^{13}C NMR (101 MHz, DMSO-d_6) δ 151.6, 135.1, 116.4, 102.8, 93.5, 66.0, 17.8, 17.5, -1.4. HRMS (ESI–): calcd for $C_{19}34_3O_4Cl_3{}^{35}Si_2$[M–H]$^-$ 487.1067, found 487.1065.

(2,6-dibromo-3,5-bis(methoxymethoxy)phenyl)(3,5-dichloro-2,6-bis(methoxymethoxy)-4-methylphenyl)methanol (19): A round bottom flask was charged with activated powdered 4 Å mol. sieves and flamed dried under vacuum then purged with Ar. **17** (1.740 g, 1 equiv, 4.83 mmol) was added and the vessel was evacuated and purged with Ar. THF (16 mL) and TMEDA (1.30 mL, 1.8 equiv, 8.70 mmol) were added, the solution was cooled to -40 °C, and n-butyllithium (2.82 mL, 2.40 molar in hexane, 1.4 equiv, 6.77 mmol) was added dropwise. The mixture was stirred for 15 min at the same temperature, then a solution of **12** (2.227 g in 7.5 mL THF, 1.2 equiv, 5.80 mmol) was added. The reaction mixture was allowed to warm to room temperature while stirring overnight. Water and brine were added, and the aqueous layer was extracted with EtOAc. The combined organic layers were washed with brine, dried over Na_2SO_4, and concentrated. The residue was purified by flash chromatography (50 g, 0-40% EtOAc/hex) to afford the title compound (2.589 g, 3.89 mmol, 81 %) as a white solid. ^{1}H NMR (400 MHz, DMSO-d_6) δ 7.04 (s, 1H), 6.63 (d, J = 6.4 Hz, 1H), 5.89 (d, J = 6.5 Hz, 1H), 5.33 – 5.24 (m, 4H), 4.84 (d, J = 4.9 Hz, 2H), 4.69 (d, J = 5.0 Hz, 2H), 3.40 (s, 12H), 2.44 (s, 3H). ^{13}C NMR (101 MHz, DMSO-d_6) δ 153.0, 150.2, 142.1, 134.9, 130.1, 124.7, 107.7, 102.9, 99.5, 95.0, 72.2, 57.4, 56.2, 18.4. HRMS (APCI+): calcd for $C_{22}H_{25}O_8{}^{79}Br_2{}^{35}Cl_2$ [M-OH]$^+$ 644.9288, found 644.9300

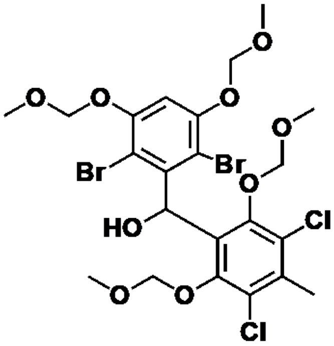

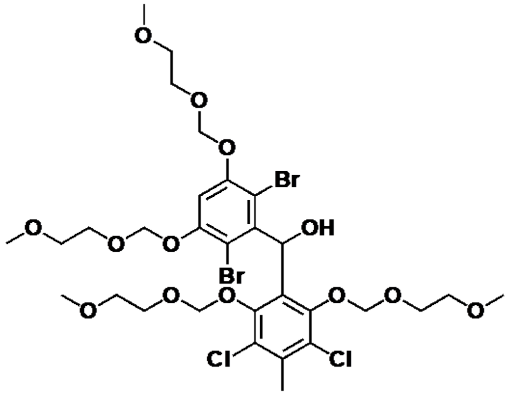

(2,6-dibromo-3,5-bis((2-methoxyethoxy)methoxy)phenyl)(3,5-dichloro-2,6-bis((2-methoxyethoxy)methoxy)-4-methylphenyl)methanol (20): A round bottom flask was charged with activated powdered 4 Å mol. sieves and flamed dried under vacuum then purged with Ar. **18** (47 mg, 1 equiv, 0.10 mmol) was added and the vessel was evacuated and purged with Ar. THF (1.0 mL) and TMEDA (28 μL, 1.8 equiv, 0.19 mmol) were added, the solution was cooled to -40 °C, and n-butyllithium (2.82 mL, 2.0 molar in hexane, 1.4 equiv, 0.15 mmol) was added dropwise. The mixture was stirred for 15 min at the same temperature, then a solution of **13** (60 mg in 0.5 mL THF, 1.2 equiv, 0.12 mmol) was added. The reaction mixture was allowed to warm to room temperature while stirring overnight. Water and brine were added, and the aqueous layer was extracted with EtOAc. The combined organic layers were washed with brine, dried over Na_2SO_4, and concentrated.

The residue was purified by flash chromatography (25 g, 0-40% EtOAc/hex) to only afford trace amounts of **20**.

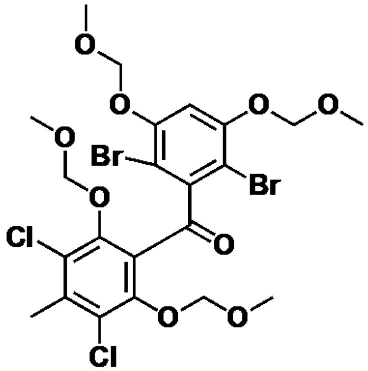

(2,6-dibromo-3,5-bis(methoxymethoxy)phenyl)(3,5-dichloro-2,6-bis(methoxymethoxy)-4-methylphenyl)methanone (21): A suspension of **20** (1.808 g, 1 equiv, 2.718 mmol) and sodium bicarbonate (1.14 g, 5 equiv, 13.6 mmol) was treated with DMP (3.46 g, 3 equiv, 8.16 mmol) at 0 °C. The reaction was allowed to warm to room temperature while stirring overnight, then quenched with sat. aq. $NaHCO_3$ and $Na_2S_2O_3$. The aqueous layer was extracted with DCM and the combined organic layers were washed with brine, dried over Na_2SO_4, and concentrated. The residue was purified by flash chromatography (50 g, 0-40% EtOAc/hex) to afford the title compound (1.719 g, 2.592 mmol, 95%) as an off-white solid. ^{1}H NMR (400 MHz, DMSO-d_6) δ 7.18 (s, 1H), 5.34 (s, 4H), 4.88 (s, 4H), 3.41 (s, 6H), 3.39 (s, 6H), 2.53 (s, 3H). ^{13}C NMR (101 MHz, $CDCl_3$) δ 189.8, 154.2, 151.6, 143.9, 141.5, 127.4, 126.2, 105.2, 104.2, 100.9, 95.7, 58.2, 56.7, 19.4. HRMS (APCI–): calcd for $C_{22}H_{23}O_9{}^{79}Br_2{}^{35}Cl_2$ $[M–H]^-$ 658.9091, found 658.9082.

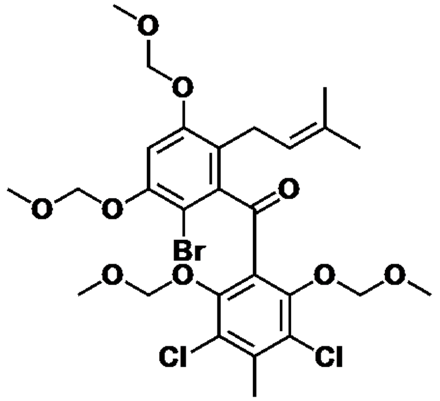

(2-bromo-3,5-bis(methoxymethoxy)-6-(3-methylbut-2-en-1-yl)phenyl)(3,5-dichloro-2,6-bis(methoxymethoxy)-4-methylphenyl)methanone (22): Freshly purified and dried CuI • 2 LiCl (50 mg, 260 μmol) was added to a pre-weighed vial containing LiCl (22 mg, 0.530 μmol) and having been stored in the oven. The vial was flame dried under vacuum and purged with Ar, then THF (1.3 mL) was added and the mixture was stirred vigorously until the solids were dissolved and a pale yellow solution formed. [*Note:* The solution of CuI • 2 LiCl should be colorless to pale yellow. Dark yellow, green or brown solutions are indicative of impure CuI and were found to give poor yields.]

A solution of **21** (267 mg, 1 equiv, 400 μmol) in anhydrous THF (4.0 mL) was cooled to -78 °C and phenyllithium (360 μL, 1.9 molar in *i*Bu_2O, 1.7 equiv, 680 μmol) was added dropwise. After 20 min at -78 °C, CuI • 2 LiCl (1.0 mL, 0.2 molar, 0.5 equiv, 200 μmol) in THF and prenyl bromide (196 μL, 95% Wt, 4 equiv, 1.60 mmol) were sequentially added. The mixture was allowed to stir for 50 min at room

temperature before the reaction was quenched by addition of a mixture of brine and aqueous NH_3 (1:1). After extraction with EtOAc, the combined organic layers were dried with Na_2SO_4, concentrated, and purified by flash chromatography (0-15% EtoAc/Hex) to afford the title compound (206 mg, 316 µmol, 78 %) as a yellowish amorphous solid. The title compound was carried forward crude. ^{1}H NMR (400 MHz, DMSO-d_6) δ 7.06 (s, 1H), 5.29 (s, 2H), 5.26 (s, 2H), 4.84 (s, 4H), 4.72 – 4.67 (m, 1H), 3.42 (s, 9H), 3.35 (s, 3H), 3.08 (d, J = 6.0 Hz, 2H), 1.52 (d, J = 1.4 Hz, 3H), 1.45 (d, J = 1.6 Hz, 3H). ^{13}C NMR (101 MHz, DMSO-d_6) δ 191.5, 154.9, 152.3, 150.5, 142.3, 139.9, 130.7, 129.0, 125.3, 123.3, 122.2, 104.2, 101.7, 100.6, 95.1, 94.1, 57.5, 56.2, 55.8, 26.1, 25.1, 18.9, 17.6. A single crystal suitable for diffraction was grown by slow evaporation of an EtOAc solution. Structure confirmed by x-ray crystallography. See x-Ray data.

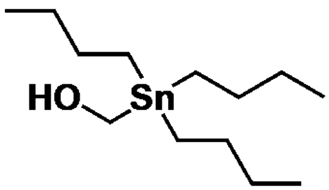

(tributylstannyl)methanol (S19): THF (10.3 mL) was added to a reaction flask, followed by bis(tributyltin) (3.00 g, 2.61 mL, 1 equiv, 5.17 mmol) and TMEDA (1.09 mL, 1.4 equiv, 7.24 mmol). The solution was cooled to 0 °C, then *n*-BuLi (2.59 mL, 2.2 M in hexane, 1.1 equiv, 5.69 mmol) was added dropwise. The mixture was stirred an additional 15 min at 0 °C, after which some gray precipitate had formed. paraformaldehyde (311 mg, 2.0 equiv, 10.3 mmol) was added as a suspension in THF (4 mL). The reaction was allowed to warm to room temperature and stirred for 2 h, then diluted with Et_2O and quenched with water. The aqueous layer was extracted with Et_2O and the combined organic layers were washed with brine, dried over Na_2SO_4, and concentrated. The crude product was purified by flash chromatography (0-20% EtOAc/hex) to afford **S2** (537 mg, 1.67 mmol, 32 %) as a colorless oil. ^{1}H NMR (400 MHz, $CDCl_3$) δ 4.02 (s, 2H), 1.58 – 1.45 (m, 6H), 1.36 – 1.24 (m, 6H), 0.95 – 0.85 (m, 15H). NMR data match literature report.[4]

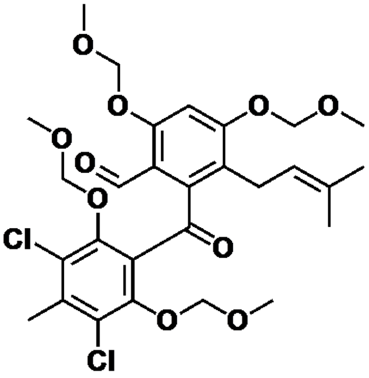

2-(3,5-dichloro-2,6-bis(methoxymethoxy)-4-methylbenzoyl)-4,6-bis(methoxymethoxy)-3-(3-methylbut-2-en-1-yl)benzaldehyde (23): 22 (364 mg, 1 equiv, 558 µmol) was added to a reaction tube and the vessel was evacuated and purged with Ar, then THF (2.8 mL) was added. The mixture was cooled to -78 °C, then phenyllithium (441 µL, 1.90 molar, 1.5 equiv, 837 µmol) was slowly added via syringe. Stirring was continued for 5 min at -78 °C before freshly distilled ethyl formate (4.51 mL, 100 equiv, 55.8 mmol) was added over ~ 30 s. The cooling bath was then removed and

after stirring additional 2 hour at room temperature the reaction was quenched by addition of aqueous NH_4Cl. The mixture was extracted with EtOAc and the combined organic layers were dried with Na_2SO_4 and concentrated under reduced pressure. The residue was purified by flash chromatography (25 g, 0-25% EtOAc/hex) to afford the title compound (70 mg, 0.12 mmol, 21 %) as a yellow amorphous solid. ^1H NMR (400 MHz, DMSO-d_6) δ 10.17 (s, 1H), 6.97 (s, 1H), 5.37 (s, 2H), 5.35 (s, 2H), 4.82 (s, 4H), 3.44 (s, 3H), 3.36 (s, 3H), 3.36 (s, 6H), 2.95 (d, J = 6.3 Hz, 2H), 2.50 (s, 3H), 1.43 (d, J = 1.4 Hz, 6H). ^{13}C NMR (201 MHz, DMSO-d_6) δ 191.4, 187.7, 160.4, 159.8, 151.6, 144.2, 140.3, 130.9, 126.7, 125.3, 121.8, 120.6, 117.4, 101.0, 100.3, 95.0, 94.0, 57.3, 56.3, 56.1, 25.2, 25.1, 19.0, 17.4. HRMS (APCI+): calcd for $C_{28}H_{35}O_{10}{}^{35}Cl_2$ $[M+H]^+$ 601.1602, found 601.1600. Structure confirmed by x-ray crystallography. See x-Ray data at bottom of document.

***tert*-butyl((2,6-dibromo-3,5-bis(methoxymethoxy)benzyl)oxy)dimethylsilane (25):**

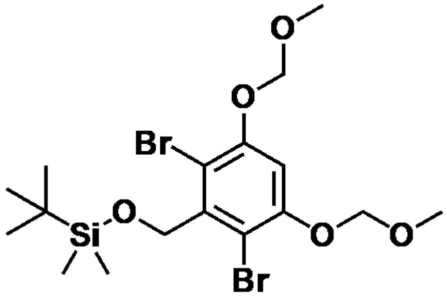

Imidazole (700 mg, 1.5 equiv, 10.3 mmol) was added to a solution of **12** (2.645 g, 1 equiv, 6.85 mmol) in THF (17 mL). TBSCl (1.240 g, 1.2 equiv, 8.22 mmol) was added, then the mixture was stirred for 1 h at 40 °C. Water was added and the aqueous layer was extracted with EtOAc. The combined organic layers were washed with brine, dried over Na_2SO_4, and concentrated. The residue was purified by flash column chromatography (50 g, 0-25% EtOAc/hex) to afford the title compound (3.184 g, 6.364 mmol, 93 %) as a white solid. ^1H NMR (400 MHz, DMSO-d_6) δ 7.08 (s, 1H), 5.30 (s, 4H), 4.97 (s, 2H), 3.42 (s, 6H), 0.89 (s, 9H), 0.12 (s, 6H). ^{13}C NMR (101 MHz, DMSO-d_6) δ 153.7, 139.7, 108.8, 104.4, 95.5, 66.2, 56.7, 26.3, 18.7, -4.8. HRMS (APCI–): calcd for $C_{17}H_{27}O_5{}^{79}Br_2{}^{28}Si$ $[M-H]^-$ 496.9995, found 497.0010.

((2-bromo-3,5-bis(methoxymethoxy)-6-(3-methylbut-2-en-1-yl)benzyl)oxy)(*tert*-butyl)dimethylsilane (26): Freshly purified and dried CuI • 2 LiCl (50 mg, 260 μmol) was added to a pre-weighed vial containing LiCl (22 mg, 0.530 μmol) and having been stored in the oven. The vial was flame dried under vacuum and purged with Ar, then THF (1.3 mL) was added and the mixture was stirred

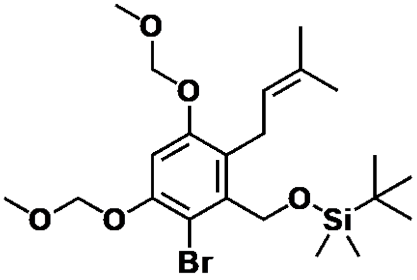

vigorously until the solids were dissolved and a pale yellow solution formed. [*Note:* The solution of CuI • 2 LiCl should be colorless to pale yellow. Dark yellow, green or brown solutions are indicative of impure CuI and were found to give poor yields.]

A solution of **25** (267 mg, 1 equiv, 400 µmol) in anhydrous THF (4.0 mL) was cooled to -78 °C and phenyllithium (360 µL, 1.9 molar in *i*Bu_2O, 1.7 equiv, 680 µmol) was added dropwise. After 20 min at -78 °C, CuI • 2 LiCl (1.0 mL, 0.2 molar, 0.5 equiv, 200 µmol) in THF and prenyl bromide (196 µL, 95% Wt, 4 equiv, 1.60 mmol) were sequentially added. The mixture was allowed to stir for 50 min at room temperature before the reaction was quenched by addition of a mixture of brine and aqueous NH_3 (1:1). After extraction with EtOAc, the combined organic layers were dried with Na_2SO_4, concentrated, and purified by flash chromatography (0-15% EtoAc/Hex) to afford the title compound (2.76 g, 5.67 mmol, 94 %). ^{1}H NMR (400 MHz, $CDCl_3$) δ 6.93 (s, 1H), 5.20 (s, 2H), 5.16 (s, 2H), 5.08 (tq, *J* = 5.2, 1.4 Hz, 1H), 4.87 (s, 2H), 3.52 (s, 3H), 3.51 – 3.47 (m, 2H), 3.46 (s, 3H), 1.77 (s, 3H), 1.66 (d, *J* = 1.4 Hz, 3H), 0.92 (s, 9H), 0.14 (s, 6H). ^{13}C NMR (101 MHz, $CDCl_3$) δ 155.1, 152.5, 139.2, 131.2, 126.5, 123.6, 109.0, 103.6, 95.7, 94.8, 62.8, 56.6, 56.2, 26.1, 25.8, 25.6, 18.6, 18.1, -5.0. HRMS (APCI+): calcd for $C_{22}H_{38}O_5{}^{79}Br^{28}Si$ $[M+H]^+$ 489.1666, found 489.1664.

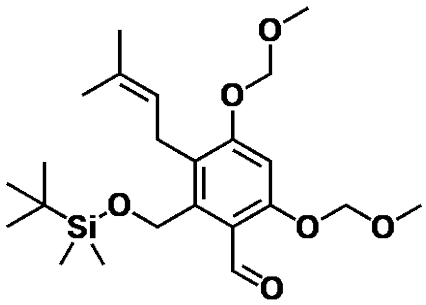

2-(((*tert*-butyldimethylsilyl)oxy)methyl)-4,6-bis(methoxymethoxy)-3-(3-methylbut-2-en-1-yl)benzaldehyde (27): Compound **26** (960 mg, 1 equiv, 1.96 mmol) and activated powdered 4 Å MS were added to a reaction tube and the vessel was evacuated and purged with Ar, then THF (9.81 mL) was added. The mixture was cooled to -78 °C, then phenyllithium (1.42 mL, 1.8 molar, 1.3 equiv, 2.55 mmol) was slowly added via syringe. Stirring was continued for 10 min at -78 °C before freshly distilled ethyl formate (3.96 mL, 25 equiv, 49.0 mmol) was added over ~ 30 s. The cooling bath was then removed and after stirring additional 1 h at room temperature the reaction was quenched by addition of sat. aq. NH_4Cl. The mixture was extracted with EtOAc, and the combined organic layers were dried over Na_2SO_4 and concentrated. The residue was purified by flash chromatography (25 g, 0-50% EtOAc/hex) to afford the title compound (633 mg, 1.44 mmol, 74 %) as a pale yellow solid. ^{1}H NMR (400 MHz, $CDCl_3$) δ 10.50 (s, 1H), 6.89 (s, 1H), 5.24 (s, 2H), 5.23 (s, 2H), 5.08 – 5.02 (m, 3H), 3.50 (s, 3H), 3.48 – 3.44 (m, 5H), 1.77 (d, *J* = 1.3 Hz, 3H), 1.66 (q, *J* = 1.4 Hz, 3H), 0.90 (s, 9H), 0.11 (s, 6H). ^{13}C NMR (101 MHz, $CDCl_3$) δ 191.3, 160.1, 159.8, 141.7, 131.1, 125.6, 123.4, 119.0, 100.7, 95.2, 94.1, 57.7, 56.5, 56.3, 26.0, 25.7, 24.4, 18.5, 18.0, -5.2. HRMS (APCI+): calcd for $C_{23}H_{39}O_6{}^{28}Si$ $[M+H]^+$ 439.2510, found 439.2509.

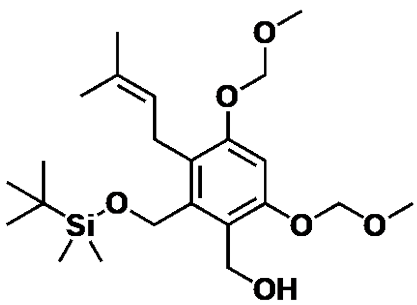

(2-(((*tert*-butyldimethylsilyl)oxy)methyl)-4,6-bis(methoxymethoxy)-3-(3-methylbut-2-en-1-yl)phenyl)methanol (28). A solution of **27** (192 mg, 1 equiv, 440 μmol) in MeOH (2.2 mL) was cooled to 0 °C. $NaBH_4$ (50 mg, 3 equiv, 1.3 mmol) was added and gas evolution was observed. The reaction was allowed to proceed at 0 °C for 10 min, then quenched with water. The mixture was extracted with EtOAc and the crude product was purified by flash chromatography (0-30% EtOAc/hex) to afford the title compound (165 mg, 370 μmol, 86 %) as a colorless oil. ^{1}H NMR (400 MHz, $CDCl_3$) δ 6.90 (s, 1H), 5.20 (s, 2H), 5.18 (s, 2H), 5.01 (tq, *J* = 5.0, 1.4 Hz, 1H), 4.79 (s, 2H), 4.75 (d, *J* = 6.6 Hz, 2H), 3.51 (s, 3H), 3.46 (s, 3H), 3.42 (d, *J* = 6.6 Hz, 2H), 2.98 (t, *J* = 6.5 Hz, 1H), 1.77 (d, *J* = 1.4 Hz, 3H), 1.65 (d, *J* = 1.4 Hz, 3H), 0.92 (s, 9H), 0.17 (s, 6H). ^{13}C NMR (101 MHz, $CDCl_3$) δ 155.3, 154.7, 139.1, 131.1, 124.5, 124.1, 123.9, 102.6, 95.6, 94.7, 59.8, 56.6, 56.5, 56.2, 26.1, 25.8, 25.0, 18.4, 18.2, -5.1. HRMS (APCI–): calcd for $C_{23}H_{39}O_6{}^{28}Si$ $[M-H]^-$ 439.2521, found 439.2527.

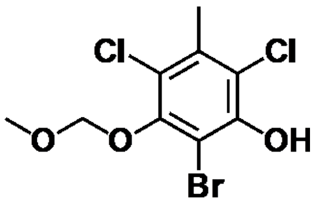

2-bromo-4,6-dichloro-3-(methoxymethoxy)-5-methylphenol (29): MOMCl was prepared *in situ*: A 3-neck round bottom flask was fitted with an addition funnel and charged with zinc(II) bromide (1.68 mg, 0.1 mol%, 7.45 μmol), then flame dried under vacuum and placed under Ar atmosphere. dimethoxymethane (659 μL, 1 equiv, 7.45 mmol) and DCM (3.7 mL) were added, then oxalyl chloride (326 μL, 0.5 equiv, 3.73 mmol) was added over a 5 min period. When the addition was complete, the reaction began to warm, then cool to room temperature over a period of ~ 1 h.

To the solution of MOMCl (1.5 equiv with respect to starting material, 7.45 mmol), **16** (1.35 g, 1 equiv, 4.96 mmol) was added and the mixture was cooled to 0 °C. DIPEA (834 mg, 1.12 mL, 1.3 equiv, 6.45 mmol) was added over a 30 min period, then the reaction was stirred for an additional 3 h. The reaction was quenched with sat. aq. NH_4Cl and the aqueous layer was extracted with DCM. The combined organic layers were washed with brine, dried over Na_2SO_4, and concentrated. The residue was purified by flash column chromatography (100 g, 0-10% EtOAc/hex) to afford, in order of elution, **17** (795 mg, 2.21 mmol, 45 %), title compound **29** (450 mg, 1.42 mmol, 29 %), and recovered **16** (319 mg, 1.17 mmol, 24 %) as white solids. ^{1}H NMR (400 MHz, DMSO-d_6) δ 10.25 (s, 1H), 5.10 (s, 2H), 3.58 (s, 3H), 2.40 (s, 3H). ^{13}C NMR (101 MHz, DMSO-d_6) δ 149.8, 149.4, 134.0, 119.6, 118.7, 106.2, 99.2, 57.8, 18.3. HRMS (APCI–): calcd for $C_9H_8O_3{}^{79}Br^{35}Cl_2$ $[M-H]^-$ 312.9039, found 312.9048.

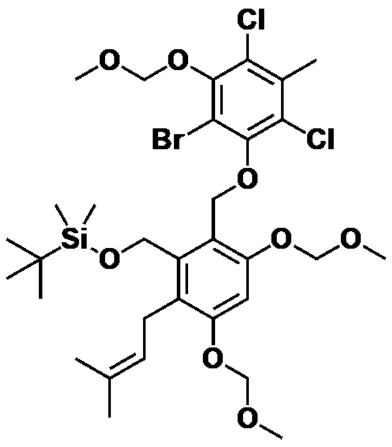

((2-((2-bromo-4,6-dichloro-3-(methoxymethoxy)-5-methylphenoxy)methyl)-3,5-bis(methoxymethoxy)-6-(3-methylbut-2-en-1-yl)benzyl)oxy)(*tert*-butyl)dimethylsilane (30): A solution of **28** (212 mg, 1 equiv, 481 µmol), **29** (304 mg, 2.0 equiv, 960 µmol), and triphenylphosphine (202 mg, 1.6 equiv, 770 µmol) in THF (2.4 mL) was treated dropwise with DIAD (107 mg, 386 µL, 1.37 molar, 1.1 equiv, 529 µmol). The reaction mixture was stirred for 30 min at room temperature, then sat. aq. $NaHCO_3$ was added, the mixture was diluted with DI water, and the aqueous layer was extracted with EtOAc. The combined organic layers were washed with brine, dried over Na_2SO_4, and concentrated. A mixture of **32** and **31** was obtained from the crude by flash chromatography (25 g, 0-10% acetone/hex), then chromatographed again (25 g, 1:1 hex/DCM, then 0-25% EtOAc in 1:1 hex/DCM) to afford a pure sample of the title compound (151 mg, 200 µmol, 43 %) as a colorless oil. ^1H NMR (800 MHz, $CDCl_3$) δ 6.87 (s, 1H), 5.30 (s, 2H), 5.18 (s, 2H), 5.11 (s, 2H), 5.08 – 5.05 (m, 3H), 4.89 (s, 2H), 3.70 (d, J = 0.8 Hz, 3H), 3.44 (s, 3H), 3.43 (d, J = 6.8 Hz, 2H), 3.39 (s, 3H), 2.47 (s, 3H), 1.77 (d, J = 1.4 Hz, 3H), 1.65 (s, 3H), 0.89 (s, 9H), 0.11 (d, J = 0.7 Hz, 6H). ^{13}C NMR (201 MHz, $CDCl_3$) δ 171.5, 156.4, 155.7, 152.4, 149.7, 140.6, 135.4, 130.8, 126.5, 125.4, 124.1, 124.1, 124.0, 117.7, 113.0, 100.8, 99.6, 94.9, 94.2, 67.2, 59.2, 58.6, 56.2, 56.2, 26.1, 25.9, 25.0, 18.7, 18.5, 18.2, -5.0. HRMS (APCI–): calcd for $C_{32}H_{46}O_8{}^{79}Br^{35}Cl_2{}^{28}Si$ [M–H]$^-$ 735.1532, found 735.1517.

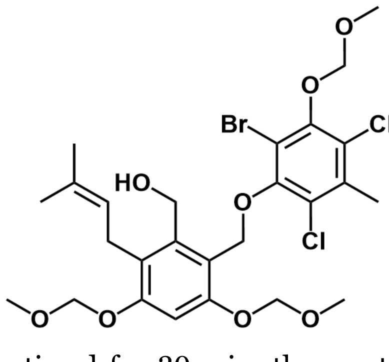

(2-((2-bromo-4,6-dichloro-3-(methoxymethoxy)-5-methylphenoxy)methyl)-3,5-bis(methoxymethoxy)-6-(3-methylbut-2-en-1-yl)phenyl)methanol (31): A solution of **30** (142 mg, 1 equiv, 190 µmol) in THF (1.9 mL) was treated with TBAF (960 µL, 1 M in THF, 5 equiv, 960 µmol) at rt. The mixture was stirred for 30 min, then water was added and the aqueous layer was extracted with EtOAc. The combined organic layers were washed with brine, dried over Na_2SO_4, and concentrated to afford the title compound (109 mg, 175 µmol, 91 %) as a colorless oil. ^1H NMR (800 MHz, $CDCl_3$) δ 6.96 (s, 1H), 5.28 (s, 2H), 5.23 (s, 2H), 5.15 (s, 2H), 5.13 – 5.10 (m, 3H), 4.93 (d, J = 6.9 Hz, 2H), 3.72 (s, 3H), 3.54 – 3.51 (m, 2H), 3.48 (s, 3H), 3.42 (s, 3H), 2.49 (s, 3H), 2.47 (t, J = 6.9 Hz, 1H), 1.82 (d, J = 1.3 Hz, 3H), 1.67

(q, J = 1.4 Hz, 3H). ^{13}C NMR (201 MHz, $CDCl_3$) δ 156.5, 155.2, 151.9, 150.0, 141.4, 135.7, 131.5, 126.4, 126.1, 124.4, 123.8, 117.6, 112.8, 101.1, 99.7, 95.2, 94.4, 67.4, 59.2, 58.7, 56.3, 56.3, 26.0, 24.8, 18.8, 18.1. HRMS (APCI–): calcd for $C_{26}H_{33}O_8{}^{79}Br^{35}Cl_3$ $[M+Cl]^-$ 657.0430, found 657.0433.

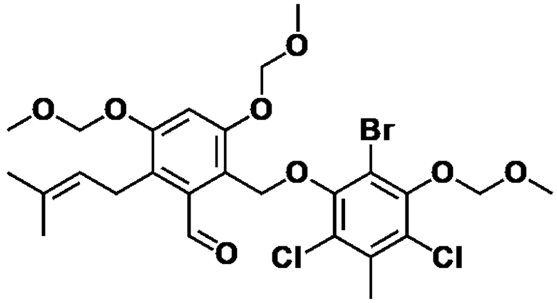

2-((2-bromo-4,6-dichloro-3-(methoxymethoxy)-5-methylphenoxy)methyl)-3,5-bis(methoxymethoxy)-6-(3-methylbut-2-en-1-yl)benzaldehyde (32): A solution of **31** (103 mg, 1 equiv, 165 μmol) in DCM (1.0 mL) was treated sequentially with DMSO-d_6 (230 μL, 20 equiv, 3.3 mmol), DIPEA (140 μL, 5 equiv, 830 μmol), and SO_3 • py (78.8 mg, 3 equiv, 495 μmol) at rt. Upon addition of SO_3 • py the colorless solution turned yellow and there was a noticeable exotherm. The reaction was complete within 15 min, at which point water was added and the aqueous layer was extracted with DCM. The combined organic layers were washed with brine, dried over Na_2SO_4, and concentrated. The residue was purified by flash column chromatography (25 g, 0-35% EtOAc/hex) to afford the title compound (91 mg, 0.15 mmol, 89 %) as a colorless oil. ^1H NMR (800 MHz, $CDCl_3$) δ 10.70 (d, J = 0.7 Hz, 1H), 7.13 (s, 1H), 5.40 (s, 2H), 5.23 (s, 2H), 5.14 (s, 2H), 5.13 (d, J = 0.6 Hz, 2H), 5.10 (ddt, J = 6.9, 5.4, 1.4 Hz, 1H), 3.70 (d, J = 0.6 Hz, 3H), 3.59 (d, J = 6.9 Hz, 2H), 3.47 (s, 3H), 3.43 (d, J = 0.6 Hz, 3H), 2.46 (d, J = 0.6 Hz, 3H), 1.75 (d, J = 1.3 Hz, 3H), 1.65 (d, J = 1.7 Hz, 3H^{13}C NMR (201 MHz, $CDCl_3$) δ 195.2, 156.5, 155.4, 152.0, 149.8, 136.6, 135.5, 132.0, 126.4, 125.8, 125.8, 123.0, 118.7, 112.9, 105.1, 99.6, 95.1, 94.5, 65.2, 58.6, 56.4, 56.4, 26.0, 24.3, 18.7, 18.1. HRMS (APCI–): calcd for $C_{26}H_{31}O_8{}^{79}Br^{35}Cl_3$ $[M+Cl]^-$ 655.0273, found 655.0284.

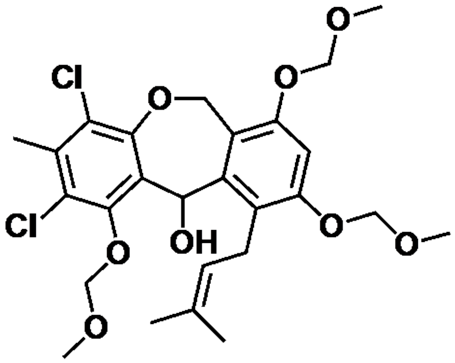

2,4-dichloro-1,7,9-tris(methoxymethoxy)-3-methyl-10-(3-methylbut-2-en-1-yl)-6,11-dihydrodibenzo[*b*,*e*]oxepin-11-ol (33): In a reaction tube under Ar, *n*-butyllithium (4.1 mg, 26 μL, 2.5 molar, 1 equiv, 64 μmol) was added dropwise to a solution of **32** (40 mg, 1 equiv, 64 μmol) in THF (2.6 mL) at -78 °C. The mixture turned yellow and the reaction was stirred at the same temperature for 2 h. Water was added and the aqueous layer was extracted with EtOAc. The combined organic layers were washed with brine, dried over Na_2SO_4, and concentrated. The crude was purified by column chromatography (20-

35% EtOAc/hex) to afford the title compound (26 mg, 48 μmol, 74 %) as a colorless oil. ^{1}H NMR (600 MHz, $CDCl_3$) δ 6.92 (s, 1H), 6.52 (d, *J* = 3.9 Hz, 1H), 5.99 – 5.93 (m, 1H), 5.60 (d, *J* = 12.5 Hz, 1H), 5.27 (d, *J* = 6.6 Hz, 1H), 5.22 – 5.19 (m, 2H), 5.17 – 5.13 (m, 2H), 5.08 – 5.04 (m, 1H), 4.97 (d, *J* = 6.6 Hz, 1H), 4.15 (d, *J* = 3.9 Hz, 1H), 3.68 (s, 3H), 3.51 (s, 3H), 3.55 – 3.44 (m, 2H), 3.43 (s, 3H), 2.46 (s, 3H), 1.78 (d, *J* = 1.3 Hz, 3H), 1.62 (q, *J* = 1.5 Hz, 3H). ^{13}C NMR (151 MHz, $CDCl_3$) δ 171.3, 155.9, 153.7, 153.1, 152.0, 139.6, 136.5, 131.8, 123.5, 123.2, 122.0, 121.8, 120.1, 119.2, 102.7, 100.5, 95.5, 94.8, 63.9, 63.3, 60.5, 58.0, 56.5, 56.2, 25.8, 24.5, 21.2, 18.6, 18.2, 14.4. HRMS (APCI–): calcd for $C_{26}H_{31}O_8{}^{35}Cl_2$ $[M-H]^-$ 541.1402, found 541.1407.

References

(1) Shen, J.; Leng, Y.; Jiang, H.; Chen, J. Compounds with 7-Member Cycle and the Pharmaceutical Use Thereof for Preventing and Treating Diabetes and Metabolism Syndrome, December 4, 2008. https://patentscope.wipo.int/search/en/WO2008144982 (accessed 2026-01-13).

(2) Slavov, N.; Cvengroš, J.; Neudörfl, J.; Schmalz, H. Total Synthesis of the Marine Antibiotic Pestalone and Its Surprisingly Facile Conversion into Pestalalactone and Pestalachloride A. *Angew Chem Int Ed* **2010**, *49* (41), 7588–7591. https://doi.org/10.1002/anie.201003755.

(3) Katoh, T.; Ohmori, O.; Iwasaki, K.; Inoue, M. Synthetic Studies on Sch 202596, an Antagonist of the Galanin Receptor Subtype GalR1: An Efficient Synthesis of (±)-Geodin, the Spirocoumaranone Part of Sch 202596. *Tetrahedron* **2002**, *58* (7), 1289–1299. https://doi.org/10.1016/S0040-4020(01)01250-9.

(4) Das, S.; Ihssen, J.; Wick, L.; Spitz, U.; Shabat, D. Chemiluminescent Carbapenem-Based Molecular Probe for Detection of Carbapenemase Activity in Live Bacteria. *Chemistry A European J* **2020**, *26* (16), 3647–3652. https://doi.org/10.1002/chem.202000217.

Spectral Data

^{1}H and ^{13}C NMR spectra begin on the following page.

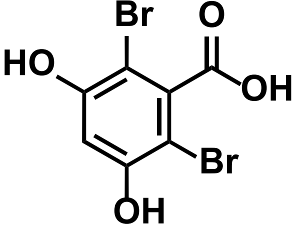

7

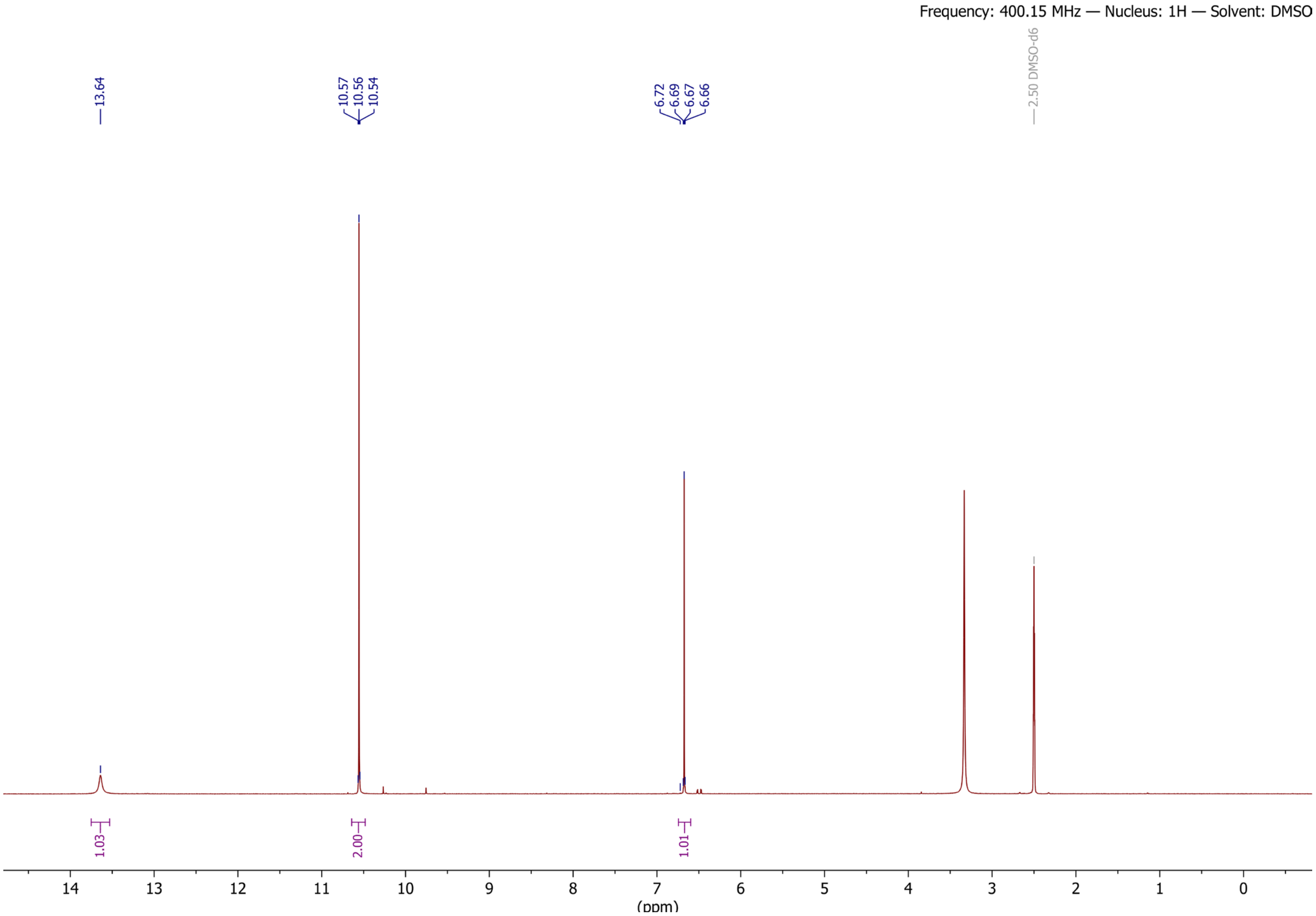

Frequency: 400.15 MHz — Nucleus: 1H — Solvent: DMSO
13.64
10.57
10.56
10.54
6.72
6.69
6.67
6.66
2.50 DMSO-d6
1.03
2.00
1.01
14
13
12
11
10
9
8
7
6
5
4
3
2
1
0
(ppm)

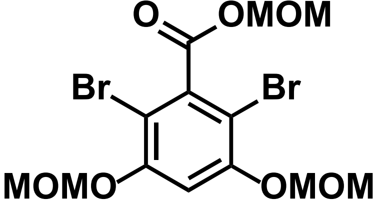

8

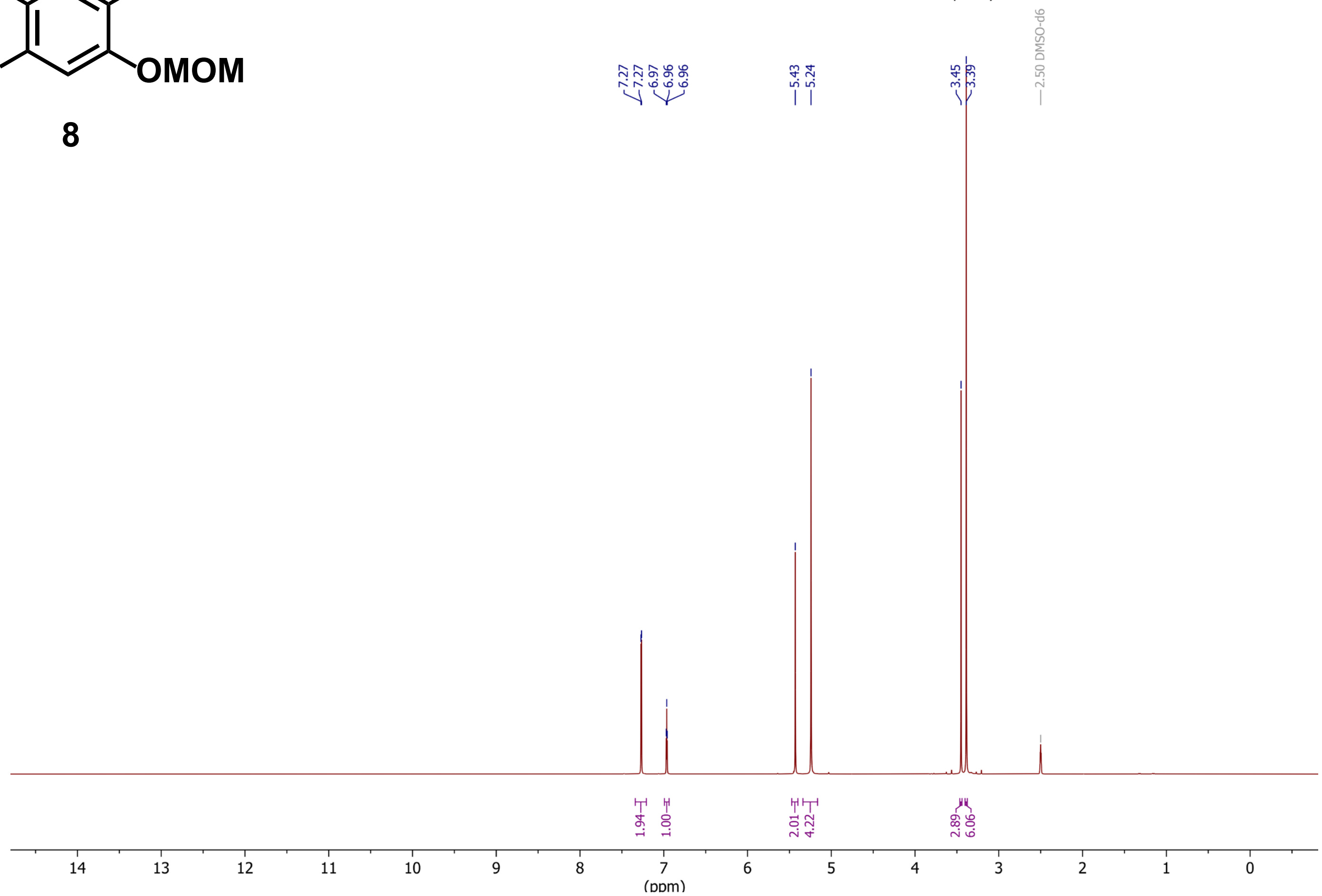
Frequency: 400.15 MHz — Nucleus: 1H — Solvent: DMSO
7.27
7.27
6.97
6.96
6.96
5.43
5.24
3.45
3.39
2.50 DMSO-d6
1.94
1.00
2.01
4.22
2.89
6.06
(ppm)

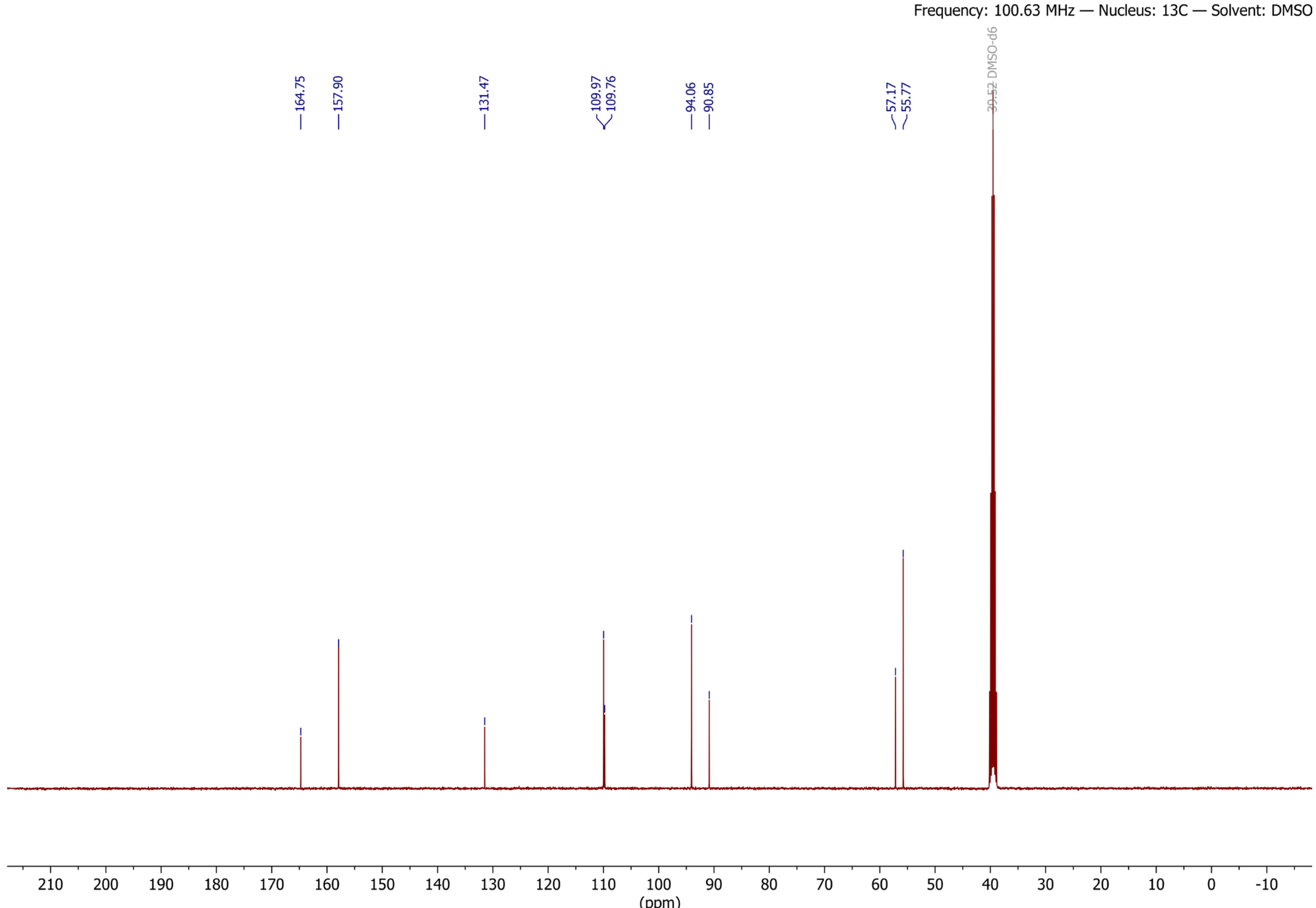
Frequency: 100.63 MHz — Nucleus: 13C — Solvent: DMSO
164.75
157.90
131.47
109.97
109.76
94.06
90.85
57.17
55.77
39.52 DMSO-d6
(ppm)

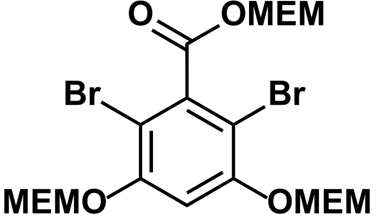
O
OMEM
Br
Br
MEMO
OMEM

9

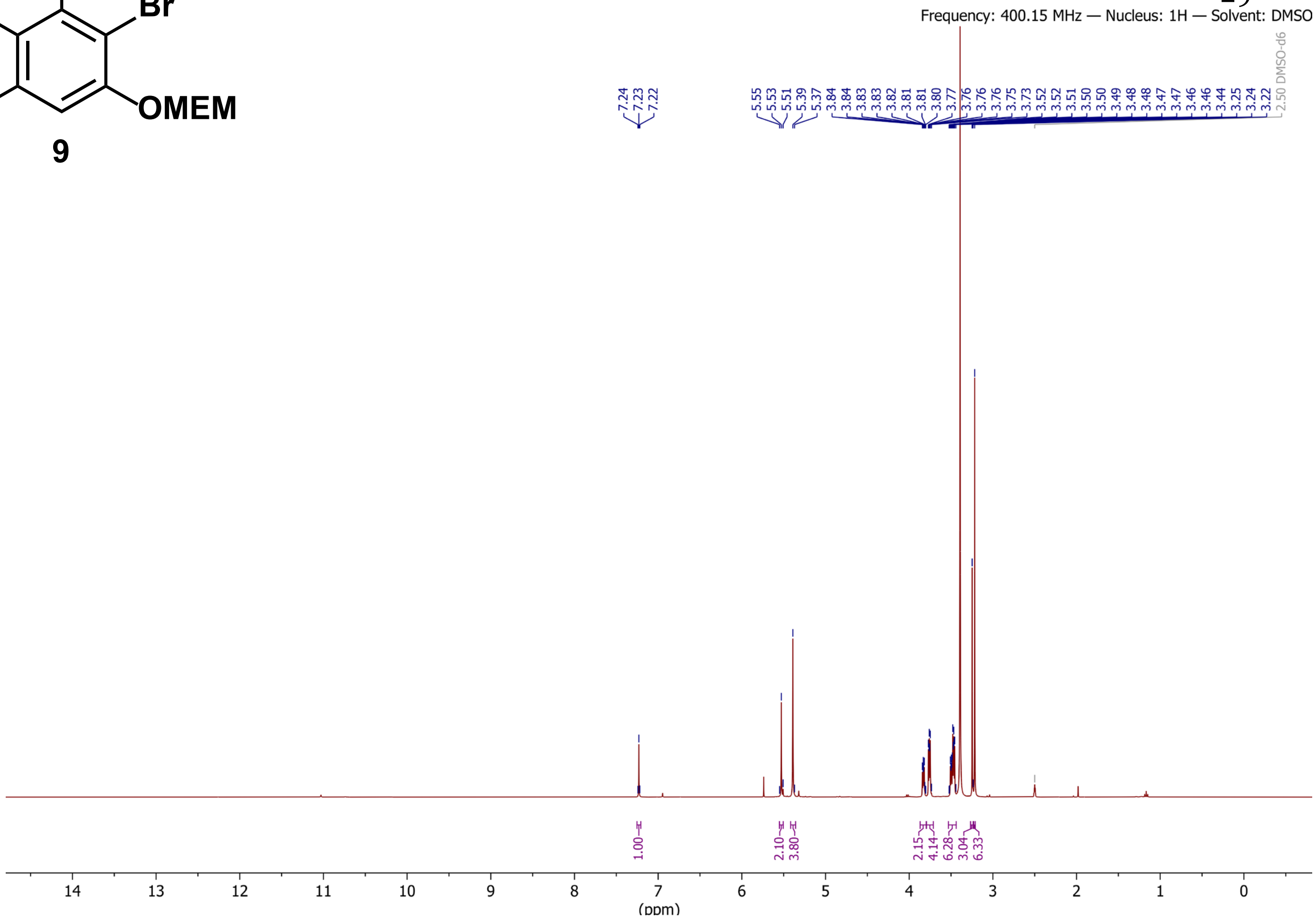
Frequency: 400.15 MHz — Nucleus: 1H — Solvent: DMSO
7.24
7.23
7.22
5.55
5.53
5.51
5.39
5.37
3.84
3.84
3.83
3.83
3.82
3.81
3.81
3.80
3.77
3.76
3.76
3.76
3.75
3.73
3.52
3.52
3.51
3.50
3.50
3.49
3.48
3.48
3.48
3.47
3.46
3.44
3.25
3.24
3.22
2.50 DMSO-d6
1.00
2.10
3.80
2.15
4.14
6.28
3.04
6.33
(ppm)

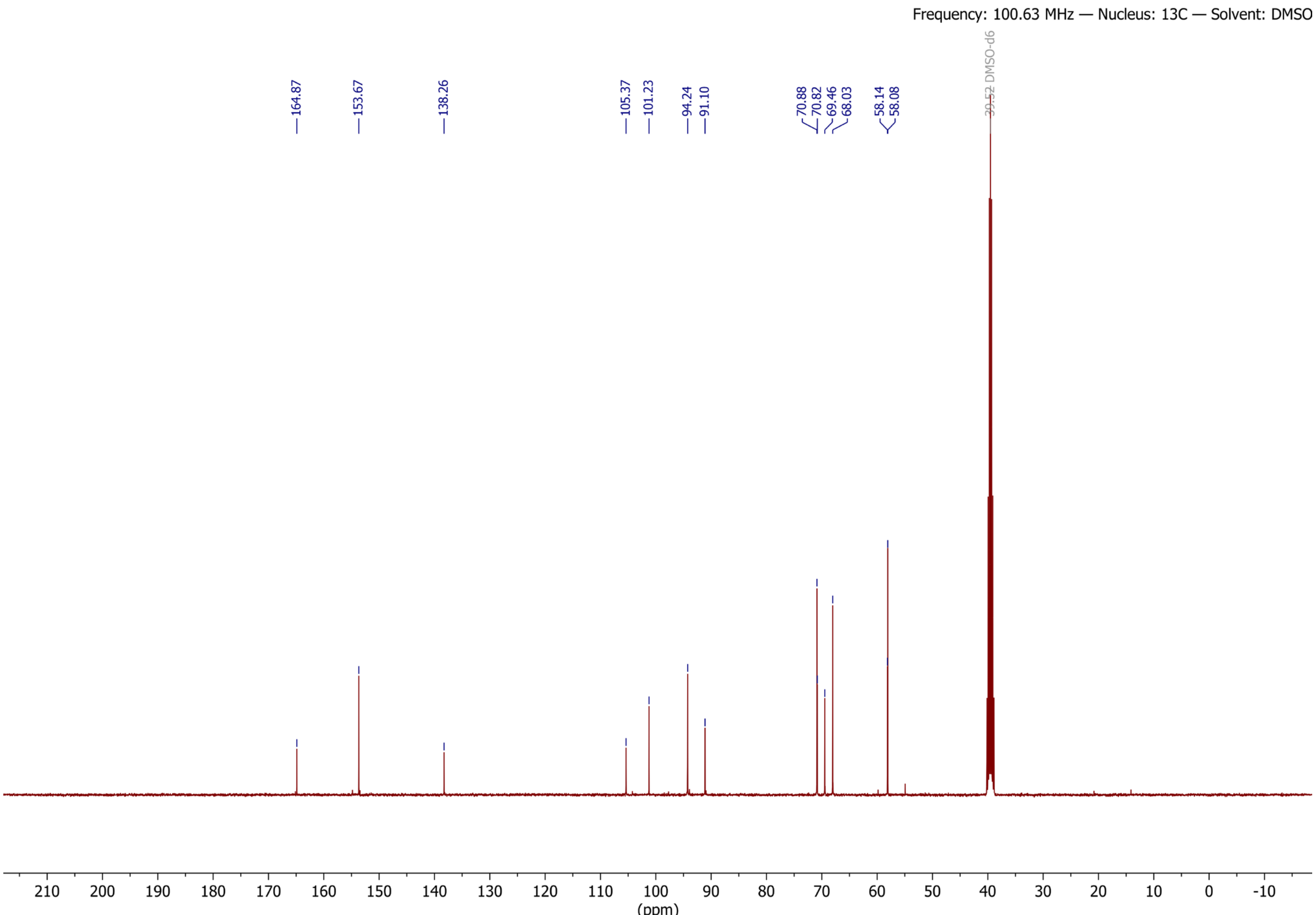
Frequency: 100.63 MHz — Nucleus: 13C — Solvent: DMSO
164.87
153.67
138.26
105.37
101.23
94.24
91.10
70.88
70.82
69.46
68.03
58.14
58.08
39.52 DMSO-d6
(ppm)

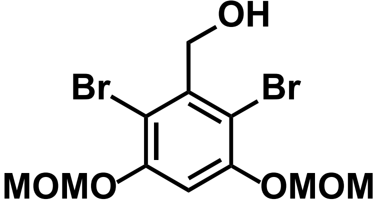

10

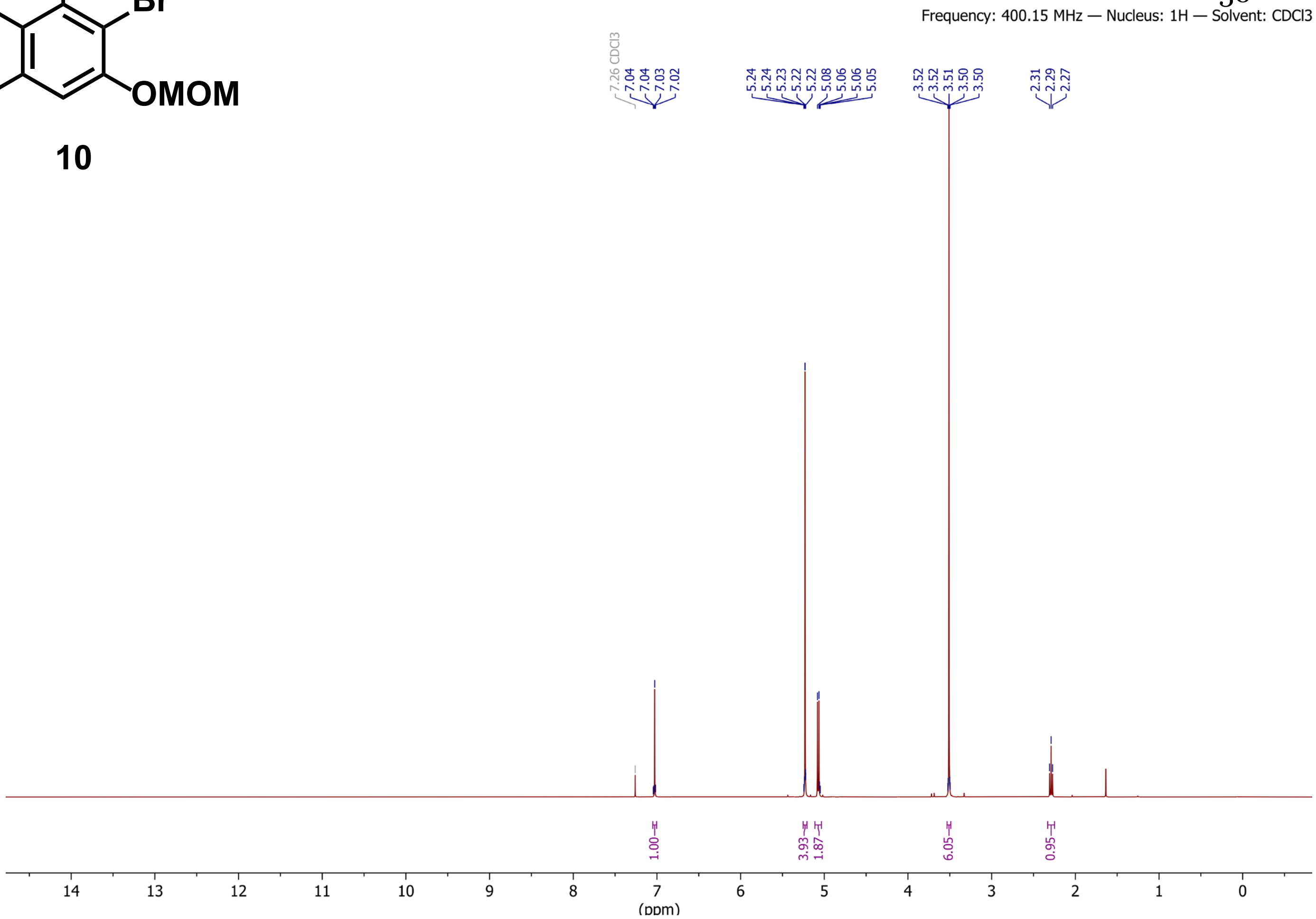
Frequency: 400.15 MHz — Nucleus: 1H — Solvent: CDCl3
7.26 CDCl3
7.04
7.04
7.03
7.02
5.24
5.24
5.23
5.22
5.22
5.08
5.06
5.06
5.05
3.52
3.52
3.51
3.50
3.50
2.31
2.29
2.27
1.00
3.93
1.87
6.05
0.95
(ppm)

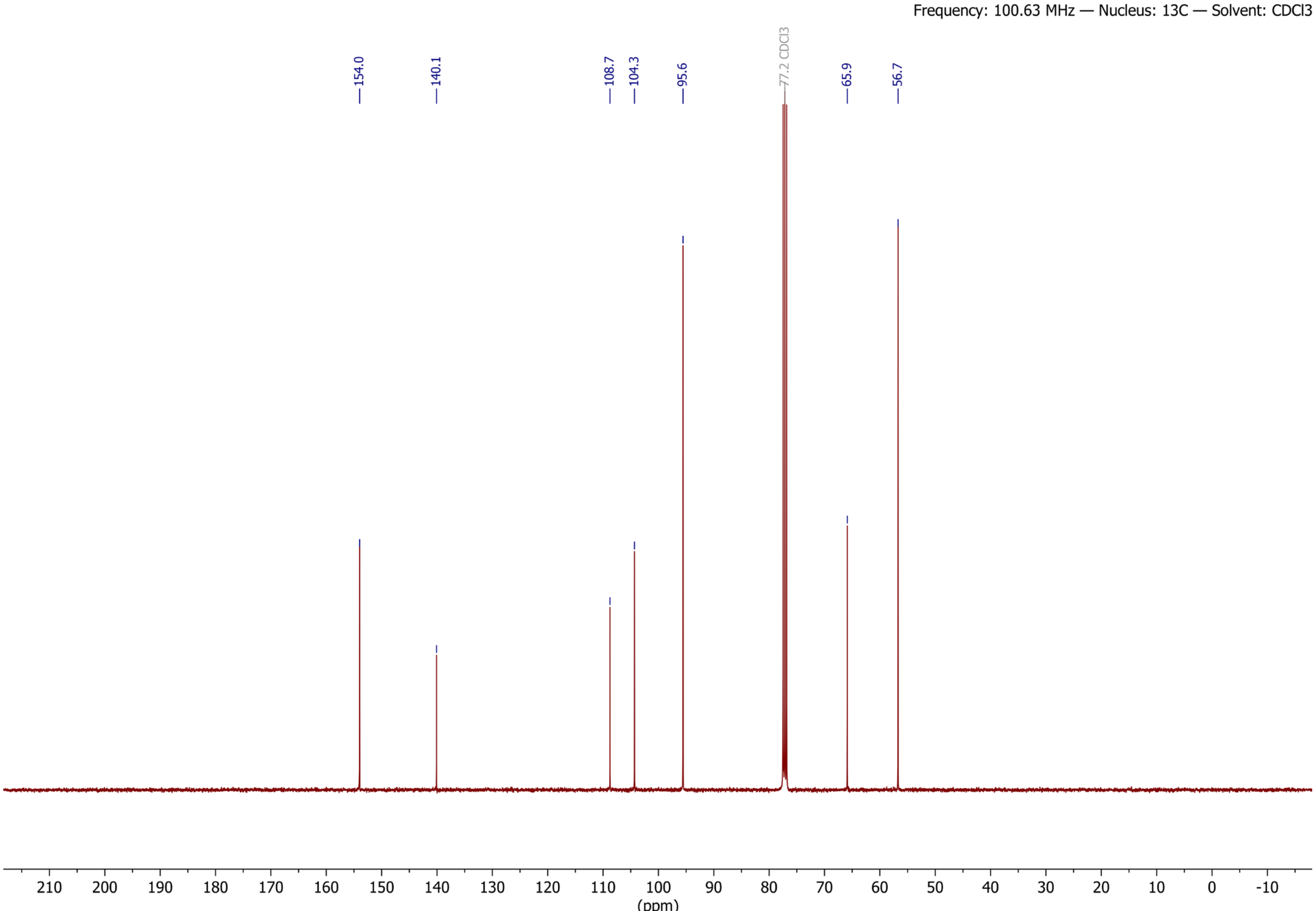
Frequency: 100.63 MHz — Nucleus: 13C — Solvent: CDCl3
154.0
140.1
108.7
104.3
95.6
77.2 CDCl3
65.9
56.7
(ppm)

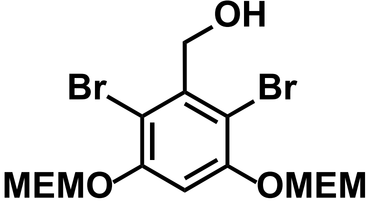

11

Frequency: 400.15 MHz — Nucleus: 1H — Solvent: CDCl3

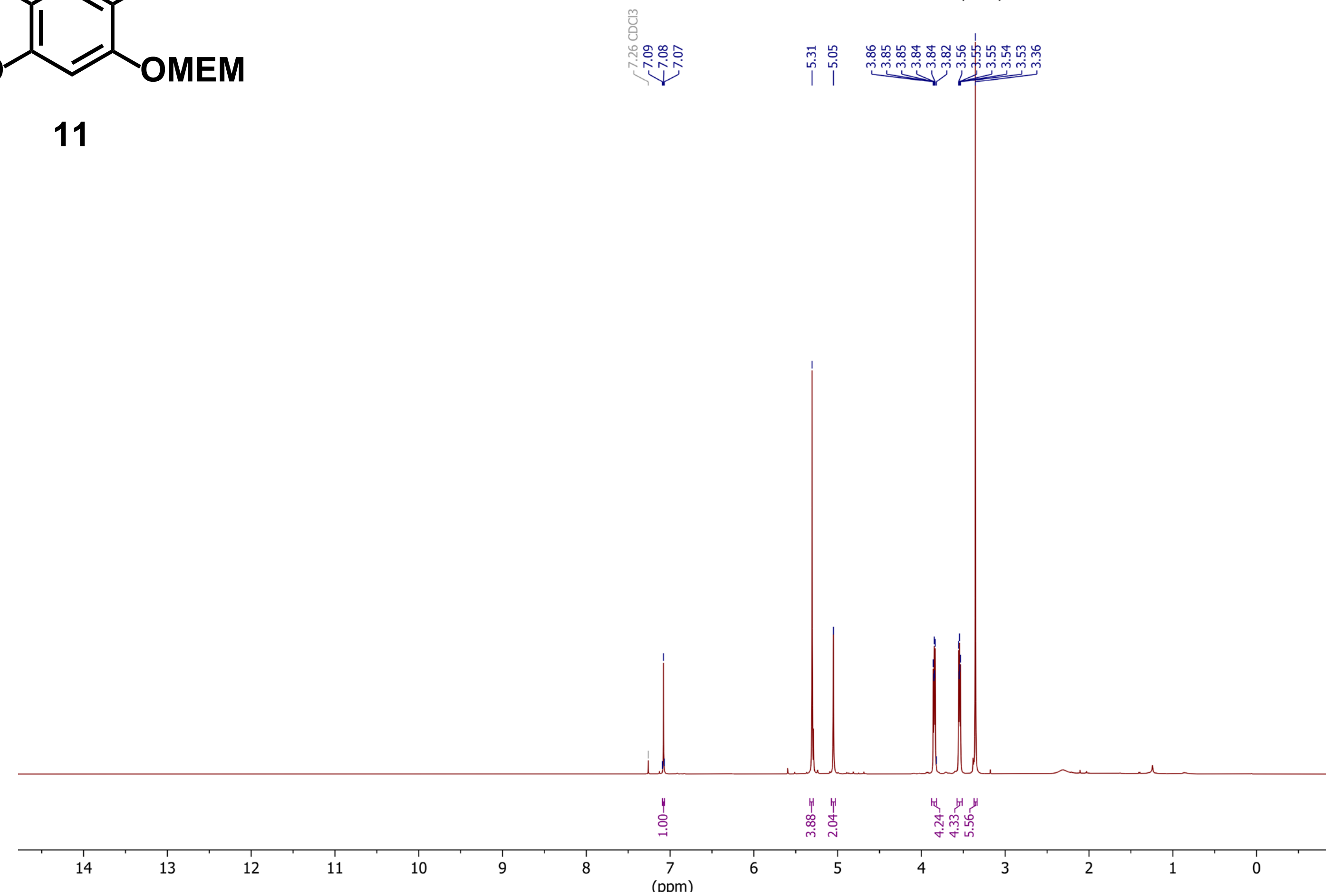

Frequency: 100.63 MHz — Nucleus: 13C — Solvent: CDCl3

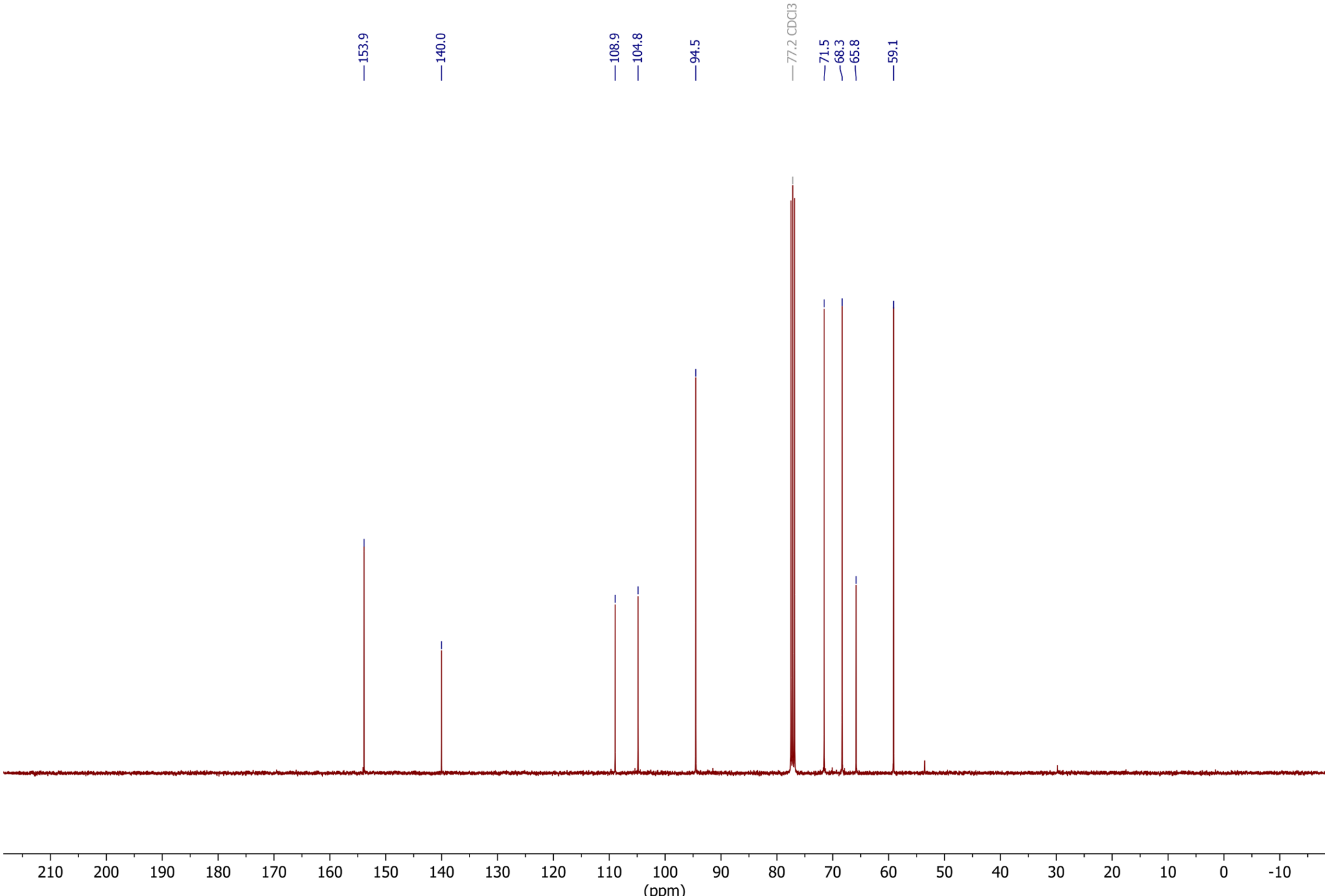

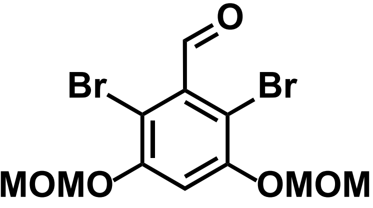

12

Frequency: 400.15 MHz — Nucleus: 1H — Solvent: DMSO

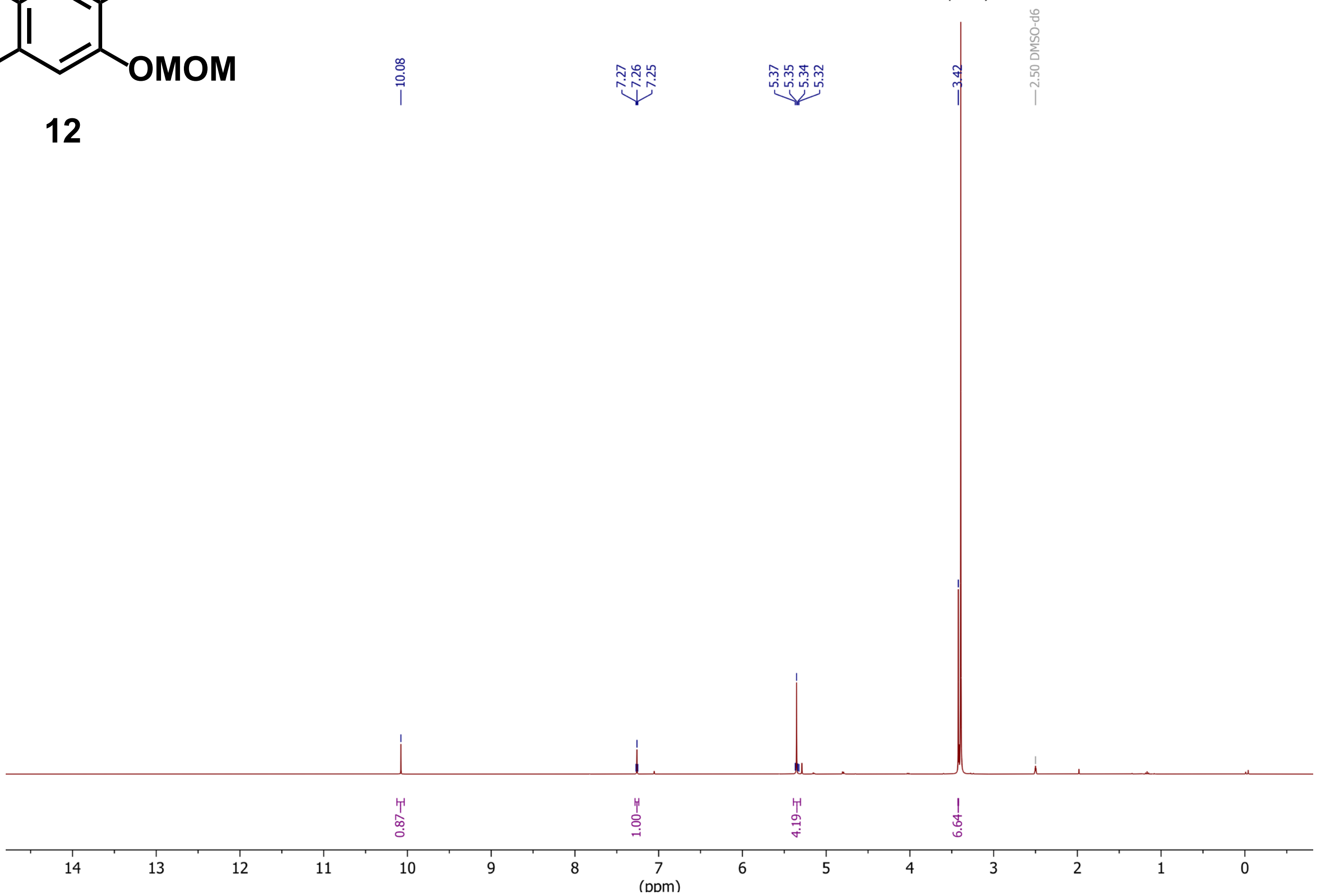

Frequency: 100.63 MHz — Nucleus: 13C — Solvent: DMSO

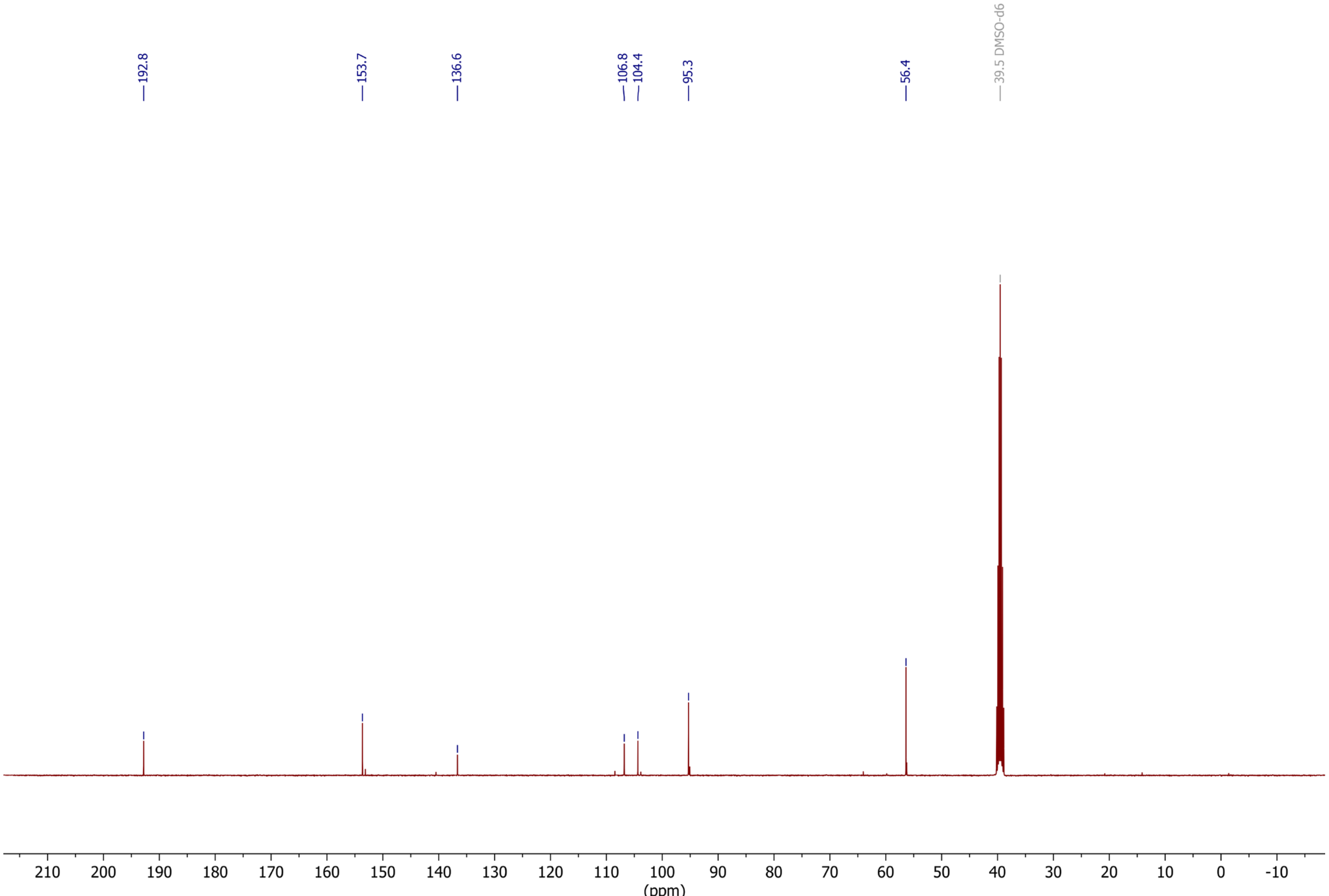

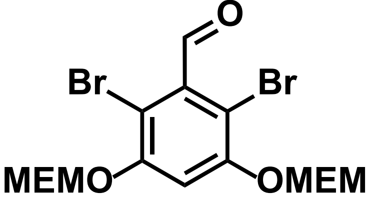

13

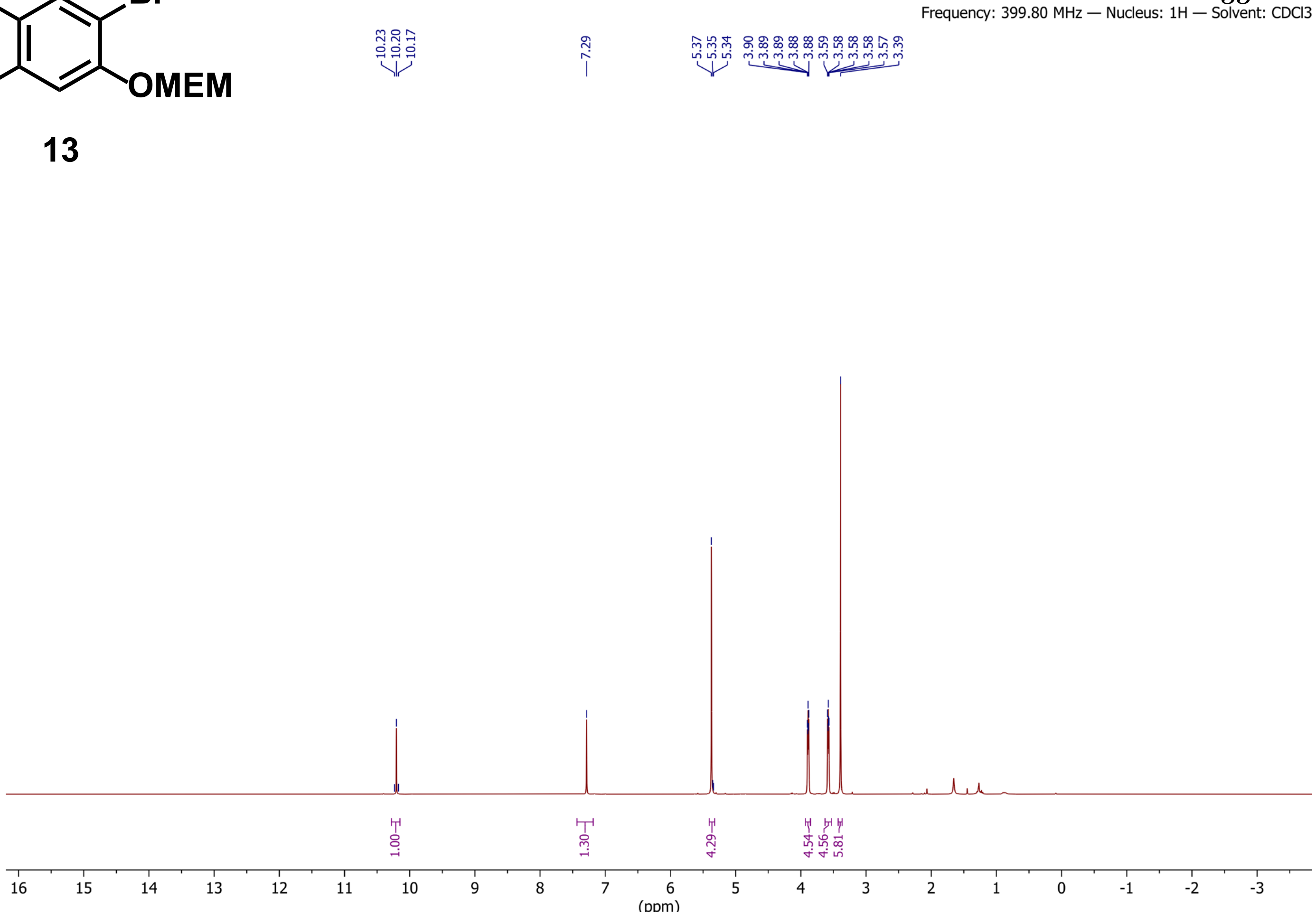
Frequency: 399.80 MHz — Nucleus: 1H — Solvent: CDCl3
10.23
10.20
10.17
7.29
5.37
5.35
5.34
3.90
3.89
3.89
3.88
3.88
3.59
3.58
3.58
3.58
3.57
3.39
1.00
1.30
4.29
4.54
4.56
5.81
(ppm)

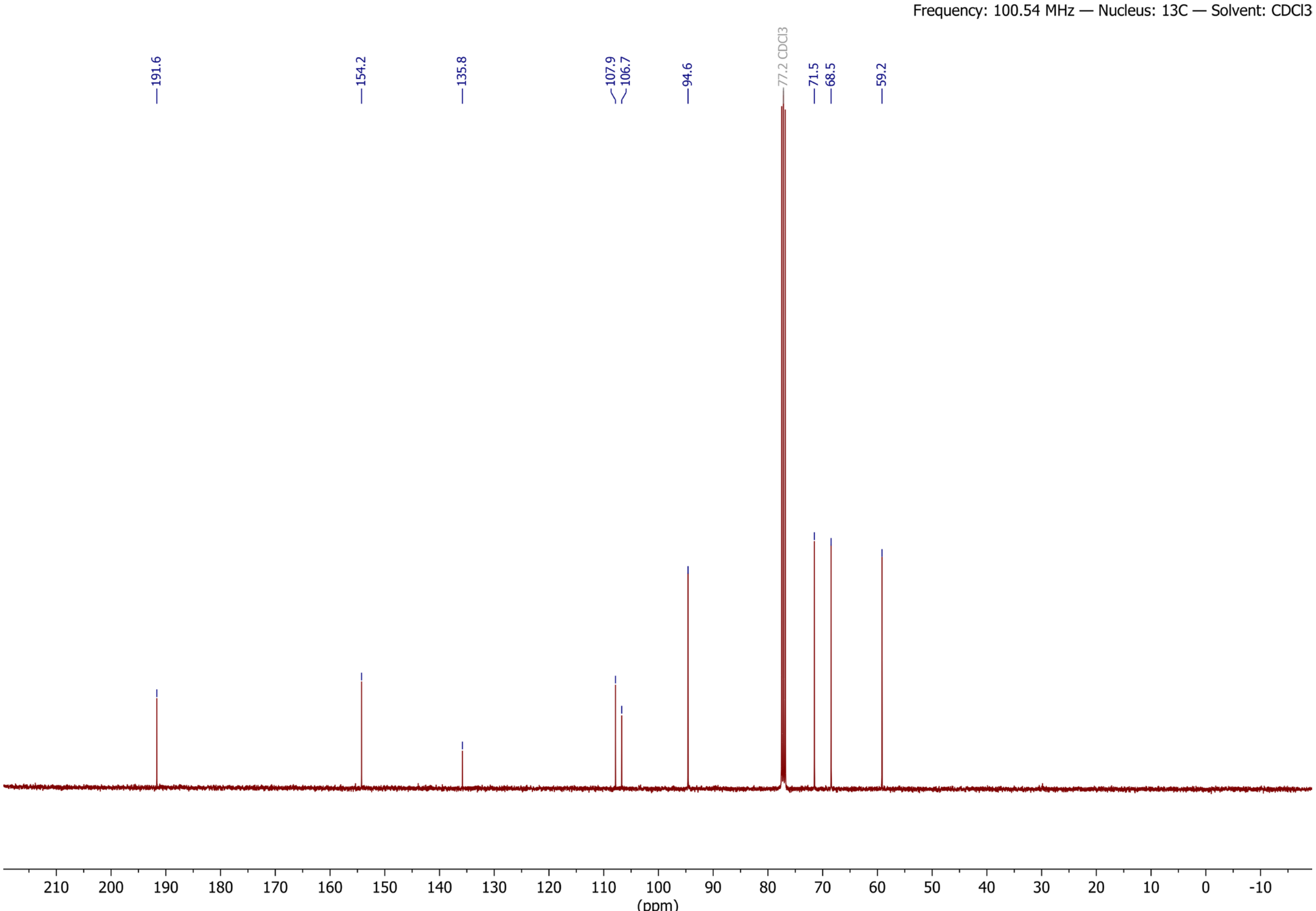
Frequency: 100.54 MHz — Nucleus: 13C — Solvent: CDCl3
191.6
154.2
135.8
107.9
106.7
94.6
77.2 CDCl3
71.5
68.5
59.2
(ppm)

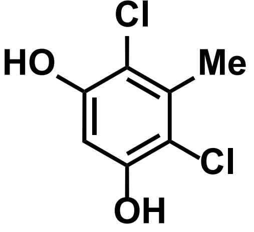

15

Frequency: 400.15 MHz — Nucleus: 1H — Solvent: DMSO

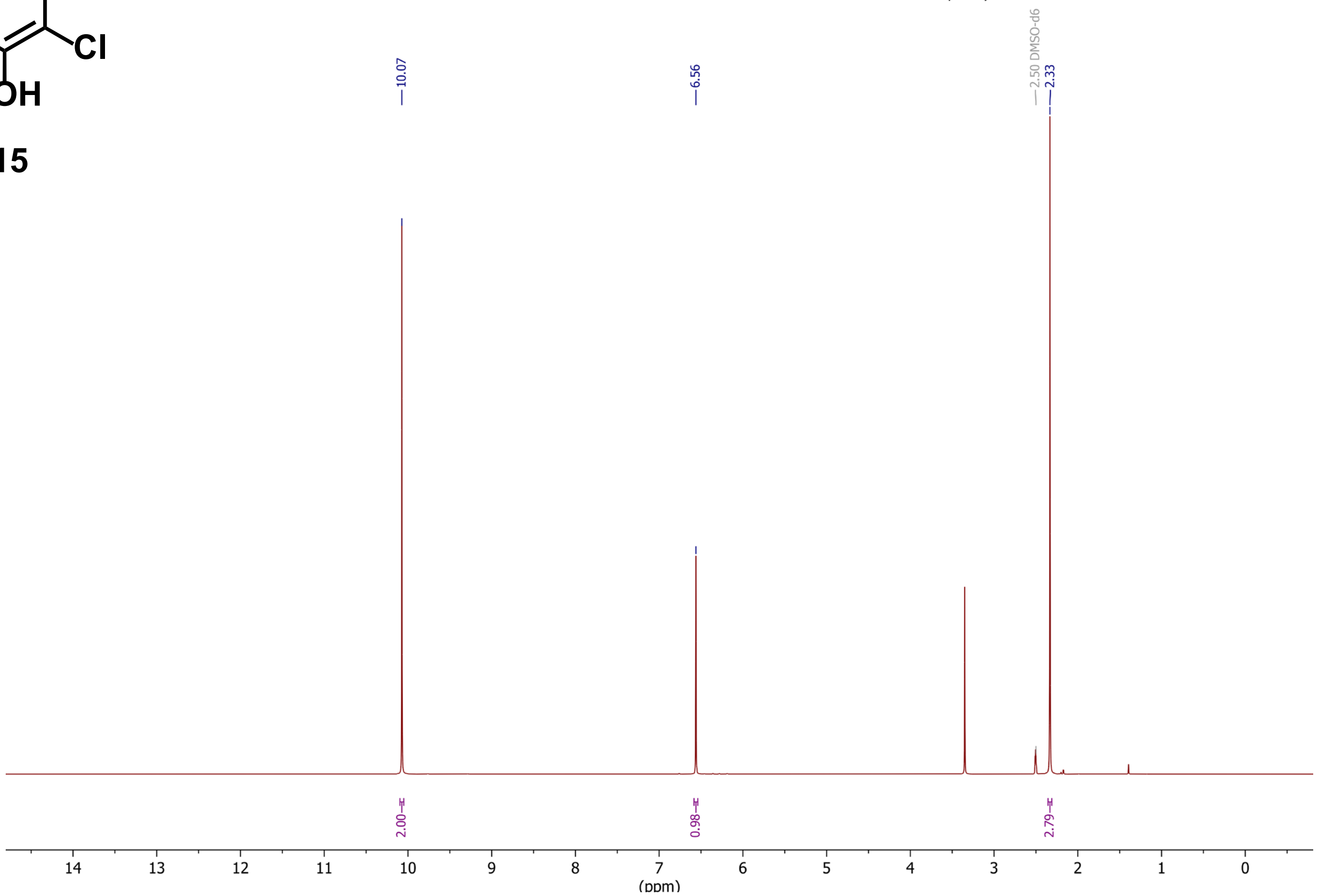

Frequency: 100.63 MHz — Nucleus: 13C — Solvent: DMSO

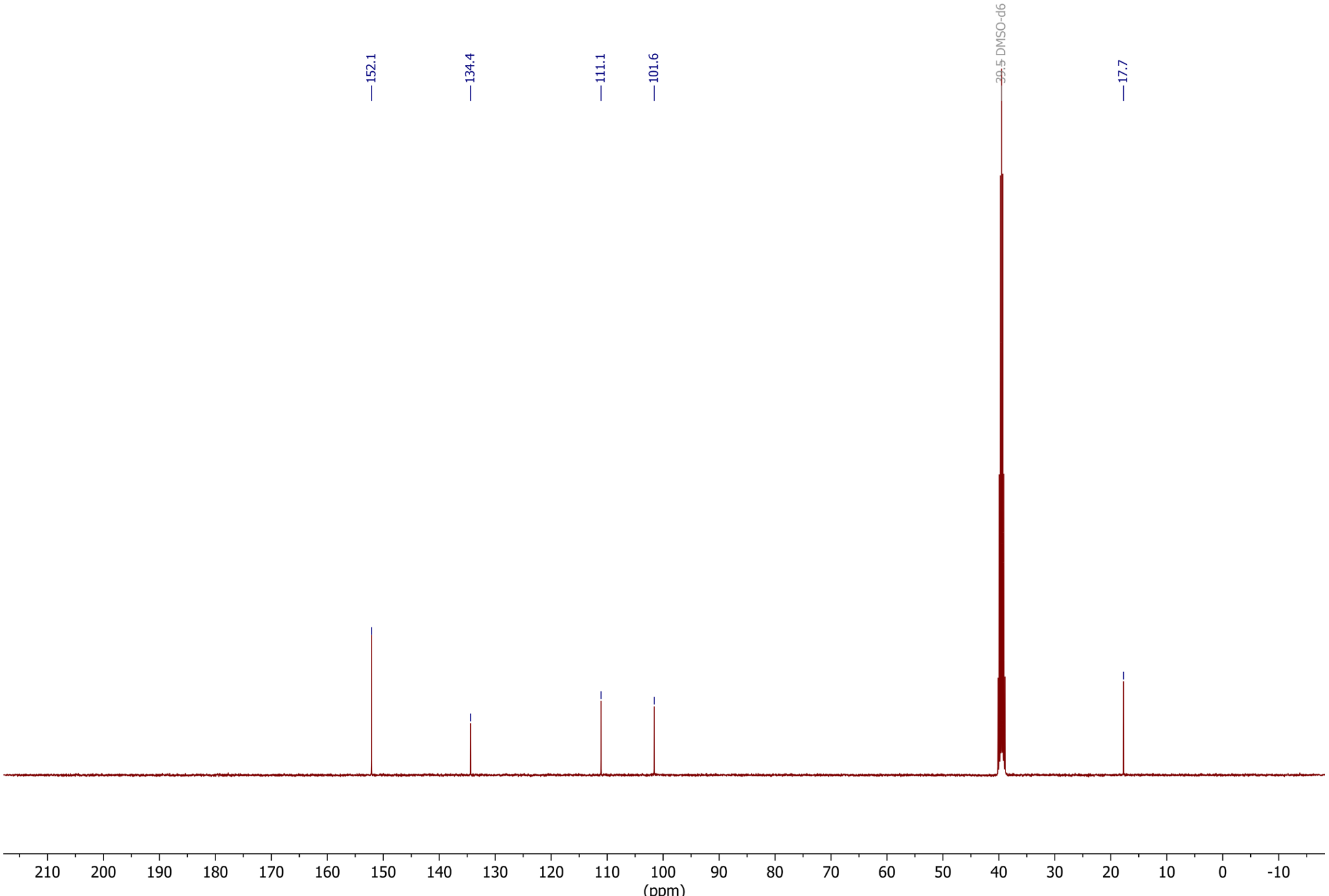

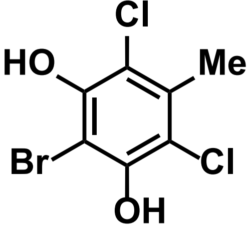

16

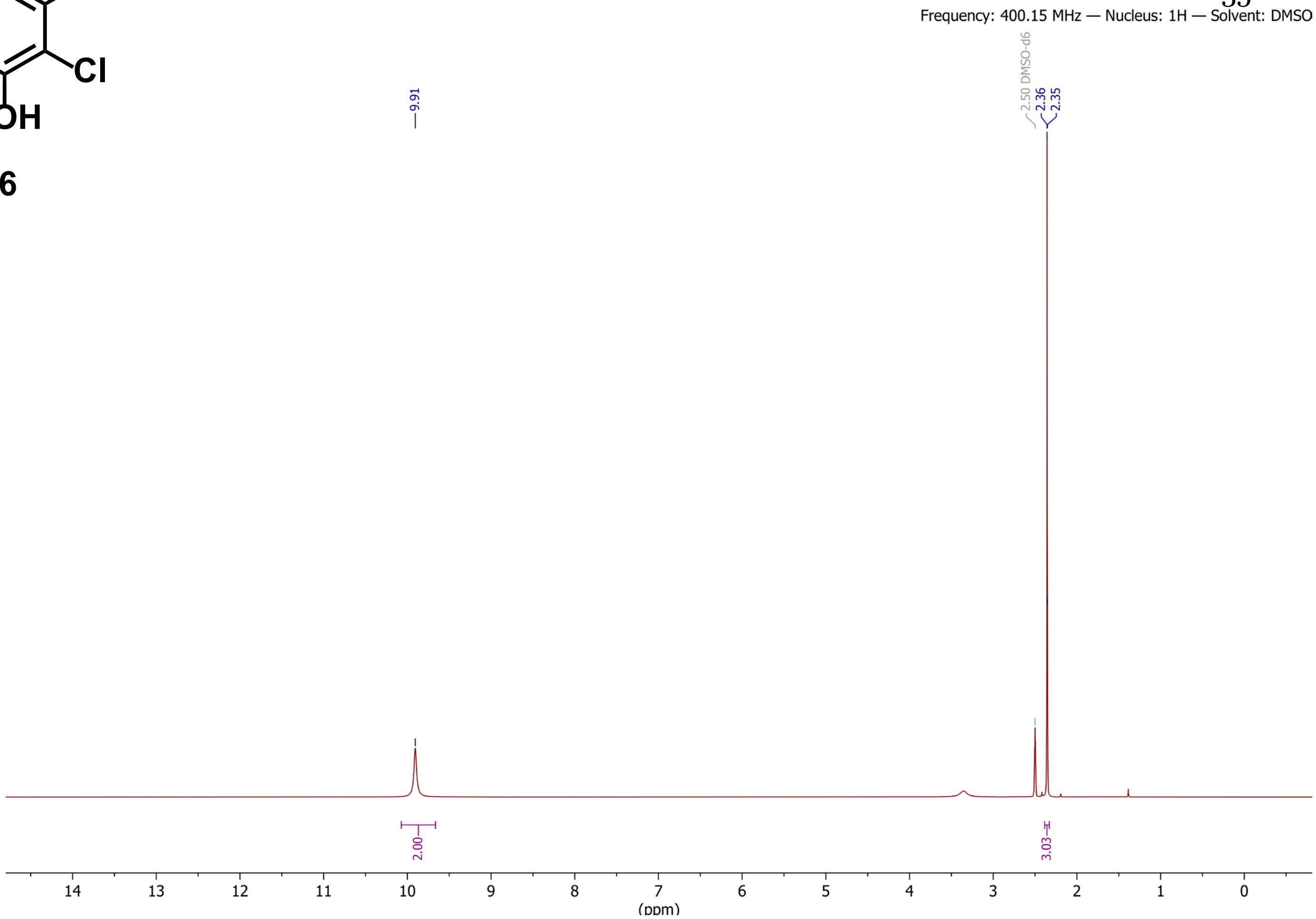
Frequency: 400.15 MHz — Nucleus: 1H — Solvent: DMSO
9.91
2.50 DMSO-d6
2.36
2.35
2.00
3.03
(ppm)

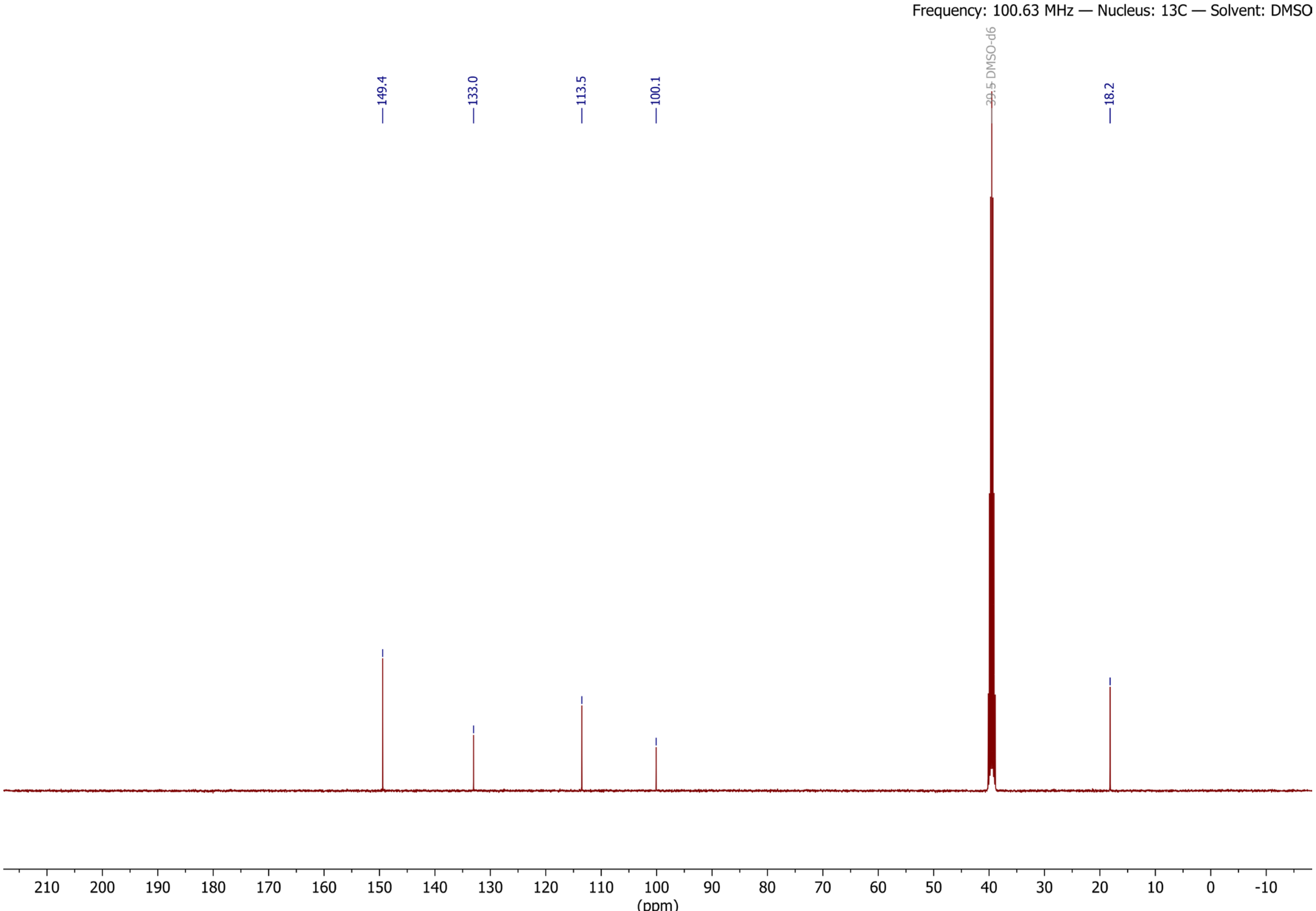
Frequency: 100.63 MHz — Nucleus: 13C — Solvent: DMSO
149.4
133.0
113.5
100.1
39.5 DMSO-d6
18.2
(ppm)

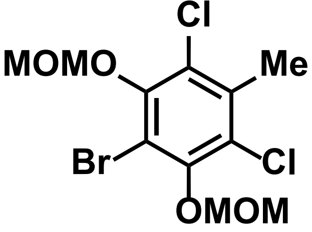
Cl
MOMO
Me
Br
Cl
OMOM

17

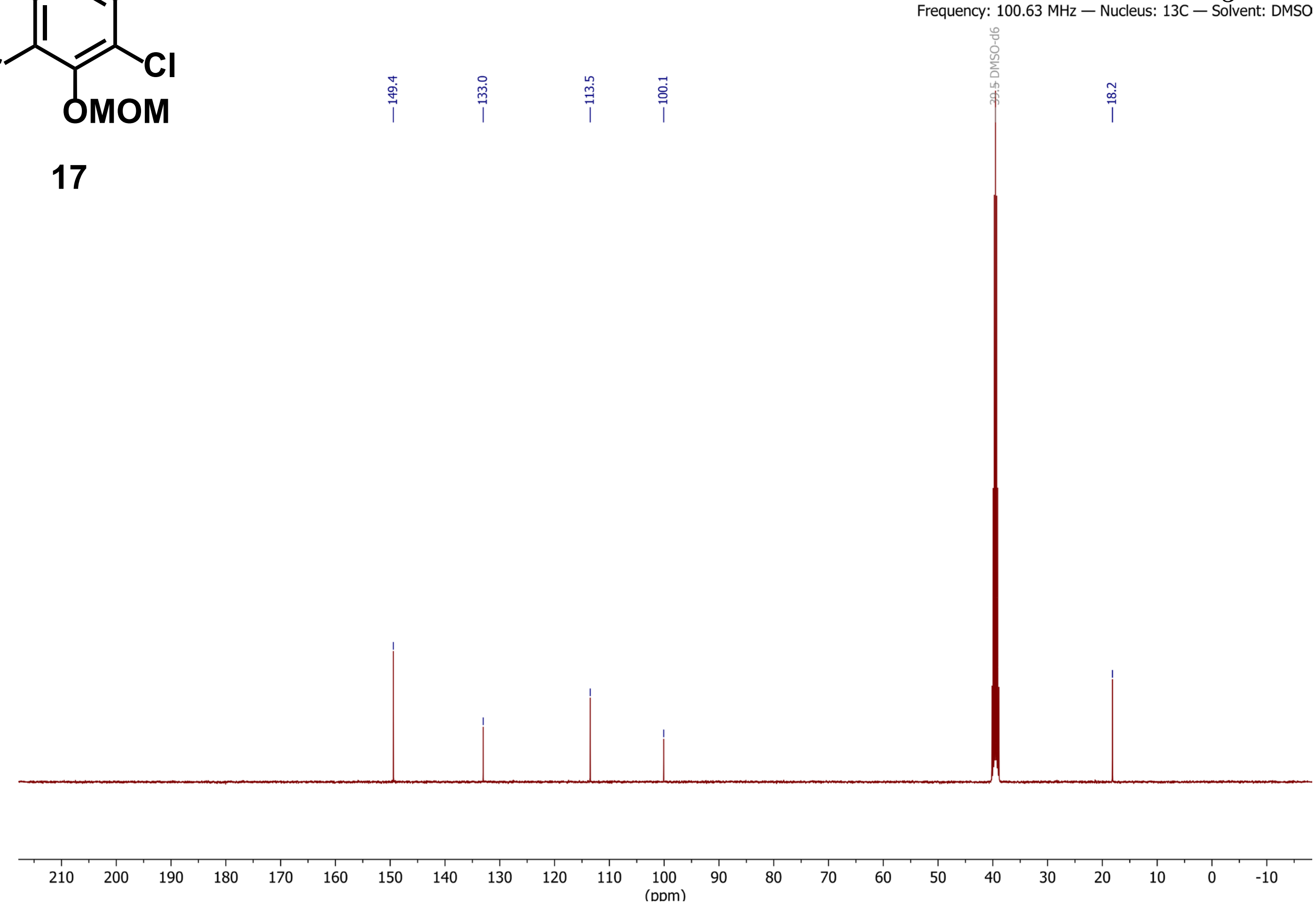
Frequency: 100.63 MHz — Nucleus: 13C — Solvent: DMSO
149.4
133.0
113.5
100.1
39.5 DMSO-d6
18.2
210 200 190 180 170 160 150 140 130 120 110 100 90 80 70 60 50 40 30 20 10 0 -10
(ppm)

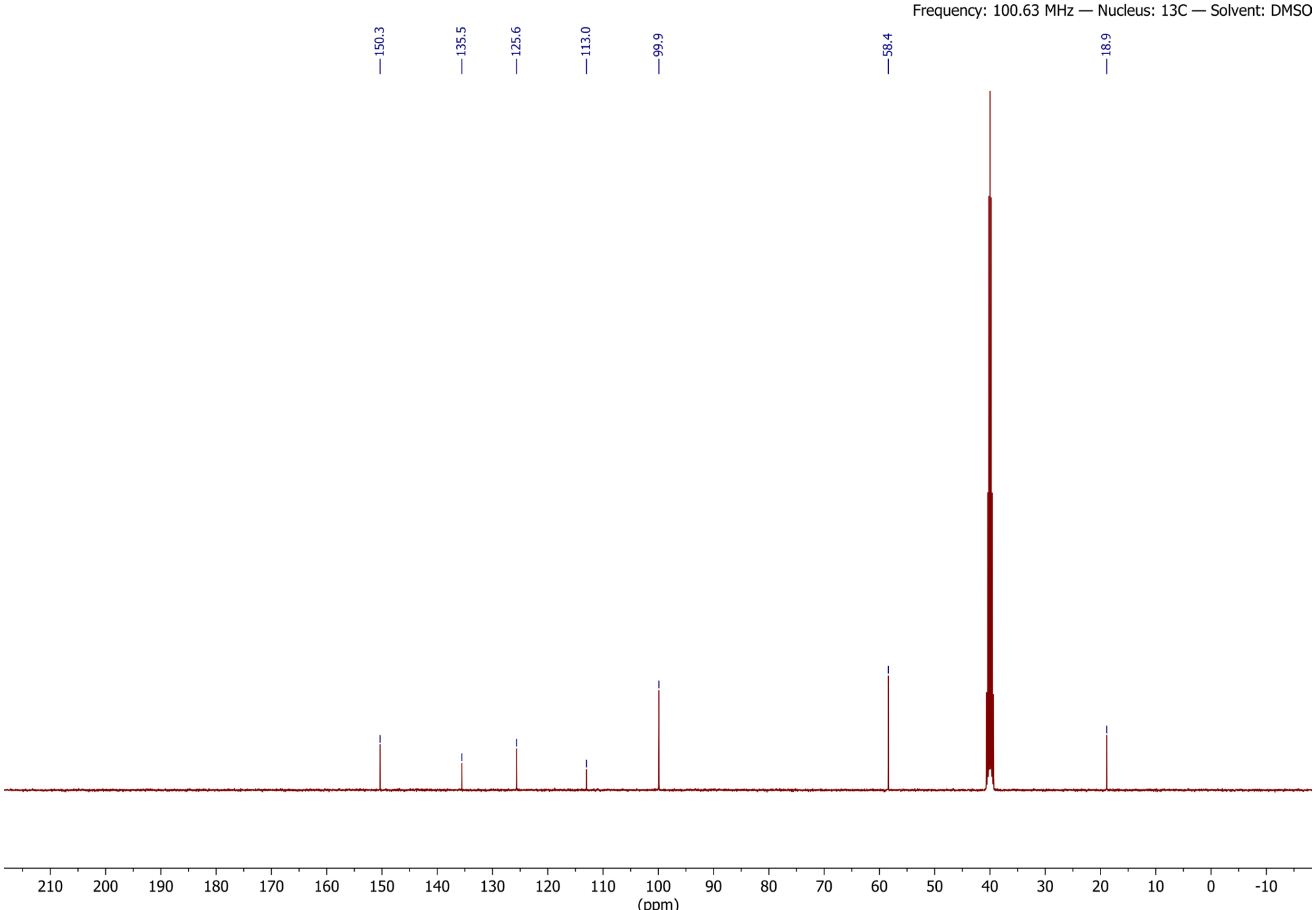
Frequency: 100.63 MHz — Nucleus: 13C — Solvent: DMSO
150.3
135.5
125.6
113.0
99.9
58.4
18.9
210 200 190 180 170 160 150 140 130 120 110 100 90 80 70 60 50 40 30 20 10 0 -10
(ppm)

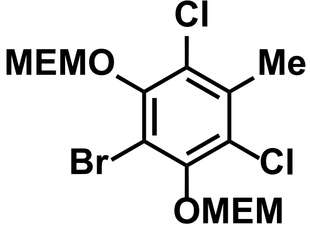
Cl
MEMO
Me
Br
Cl
OMEM

18

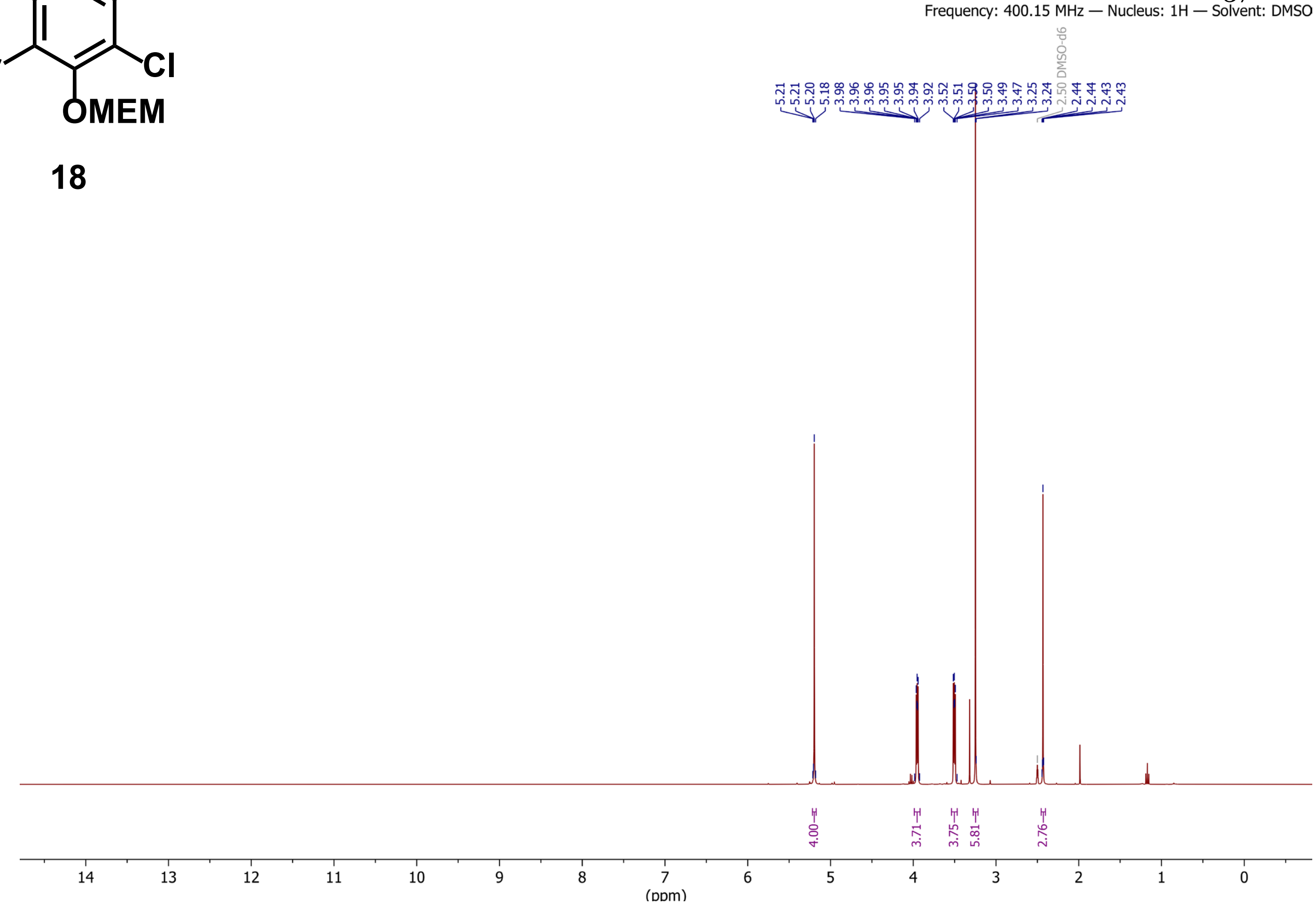
Frequency: 400.15 MHz — Nucleus: 1H — Solvent: DMSO
5.21
5.21
5.20
5.18
3.98
3.96
3.96
3.95
3.95
3.94
3.92
3.52
3.51
3.50
3.50
3.49
3.47
3.25
3.24
2.50 DMSO-d6
2.44
2.44
2.43
2.43
4.00
3.71
3.75
5.81
2.76
14
13
12
11
10
9
8
7
6
5
4
3
2
1
0
(ppm)

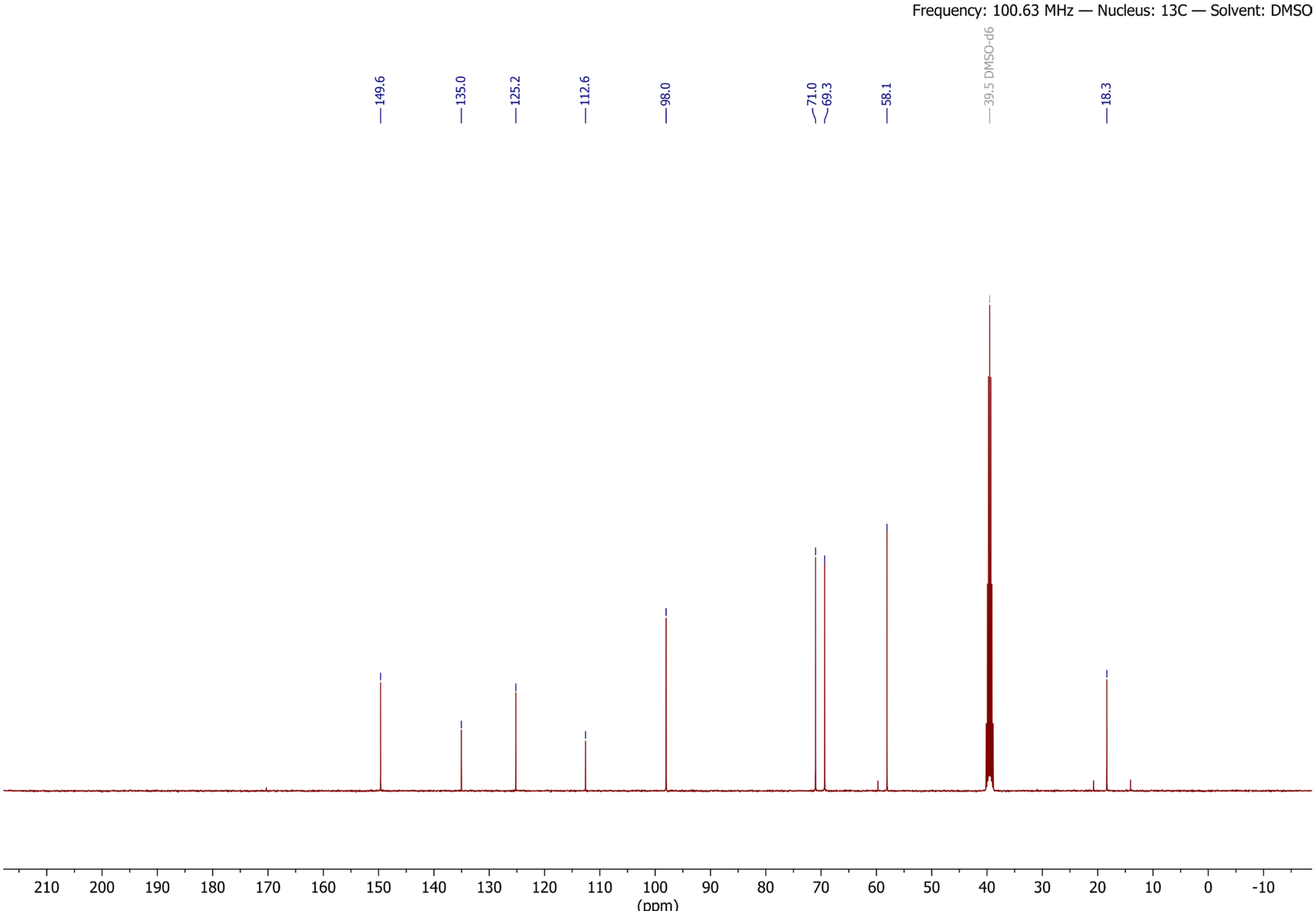
Frequency: 100.63 MHz — Nucleus: 13C — Solvent: DMSO
149.6
135.0
125.2
112.6
98.0
71.0
69.3
58.1
39.5 DMSO-d6
18.3
210
200
190
180
170
160
150
140
130
120
110
100
90
80
70
60
50
40
30
20
10
0
-10
(ppm)

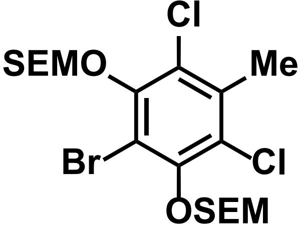

S6

Frequency: 400.15 MHz — Nucleus: 1H — Solvent: DMSO

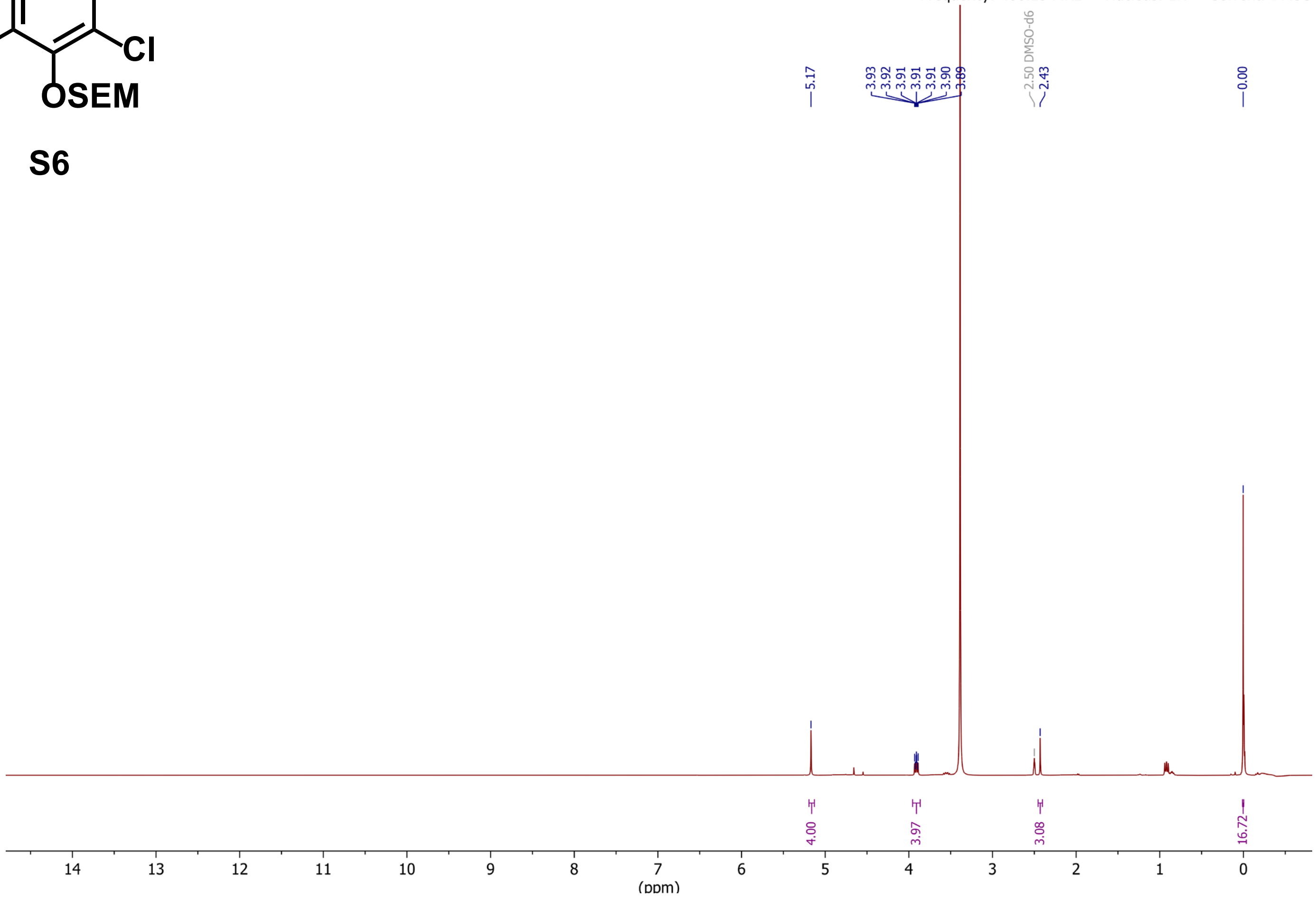

Frequency: 100.63 MHz — Nucleus: 13C — Solvent: DMSO

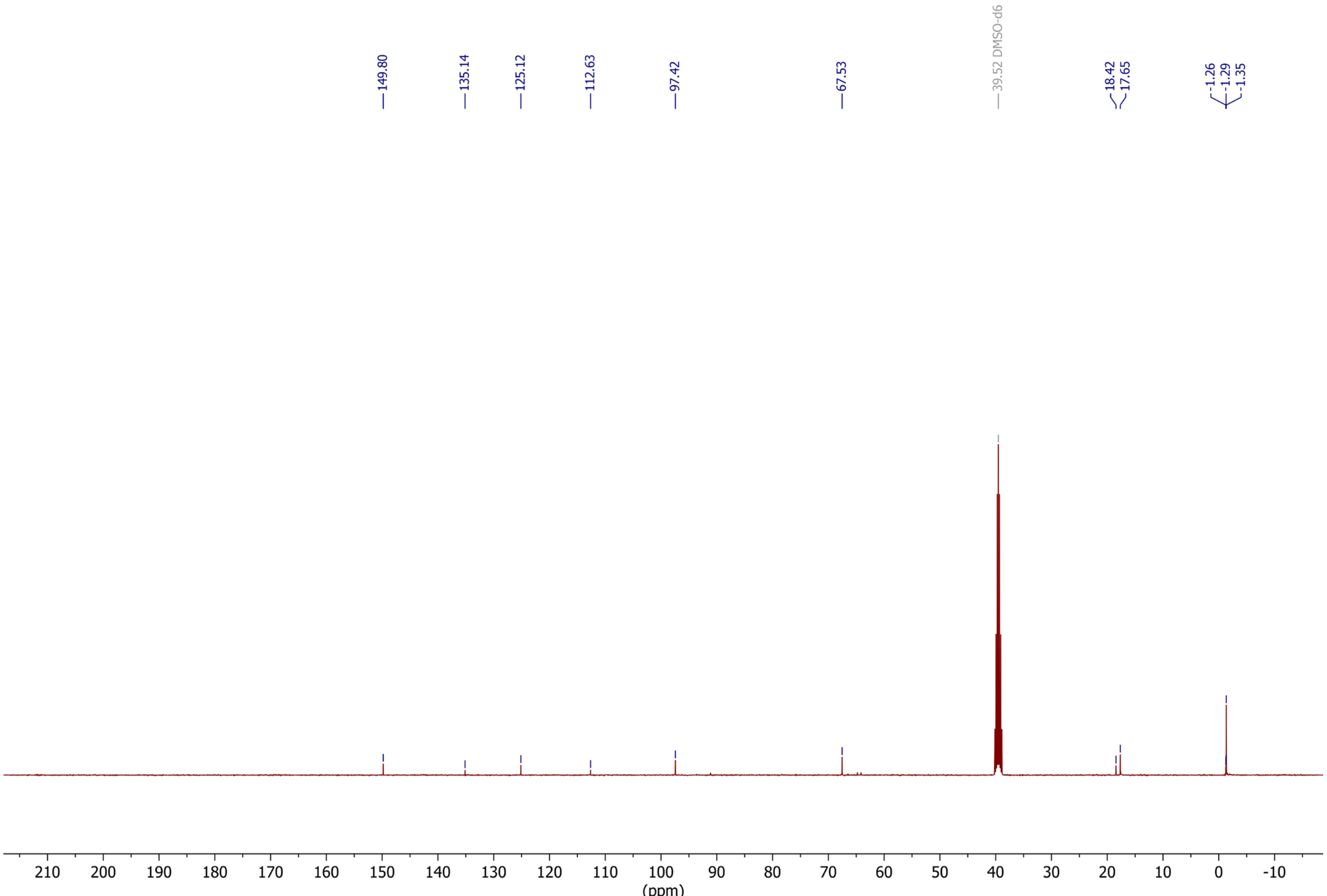

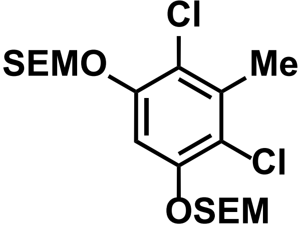
Cl
SEMO
Me
Cl
OSEM

S9

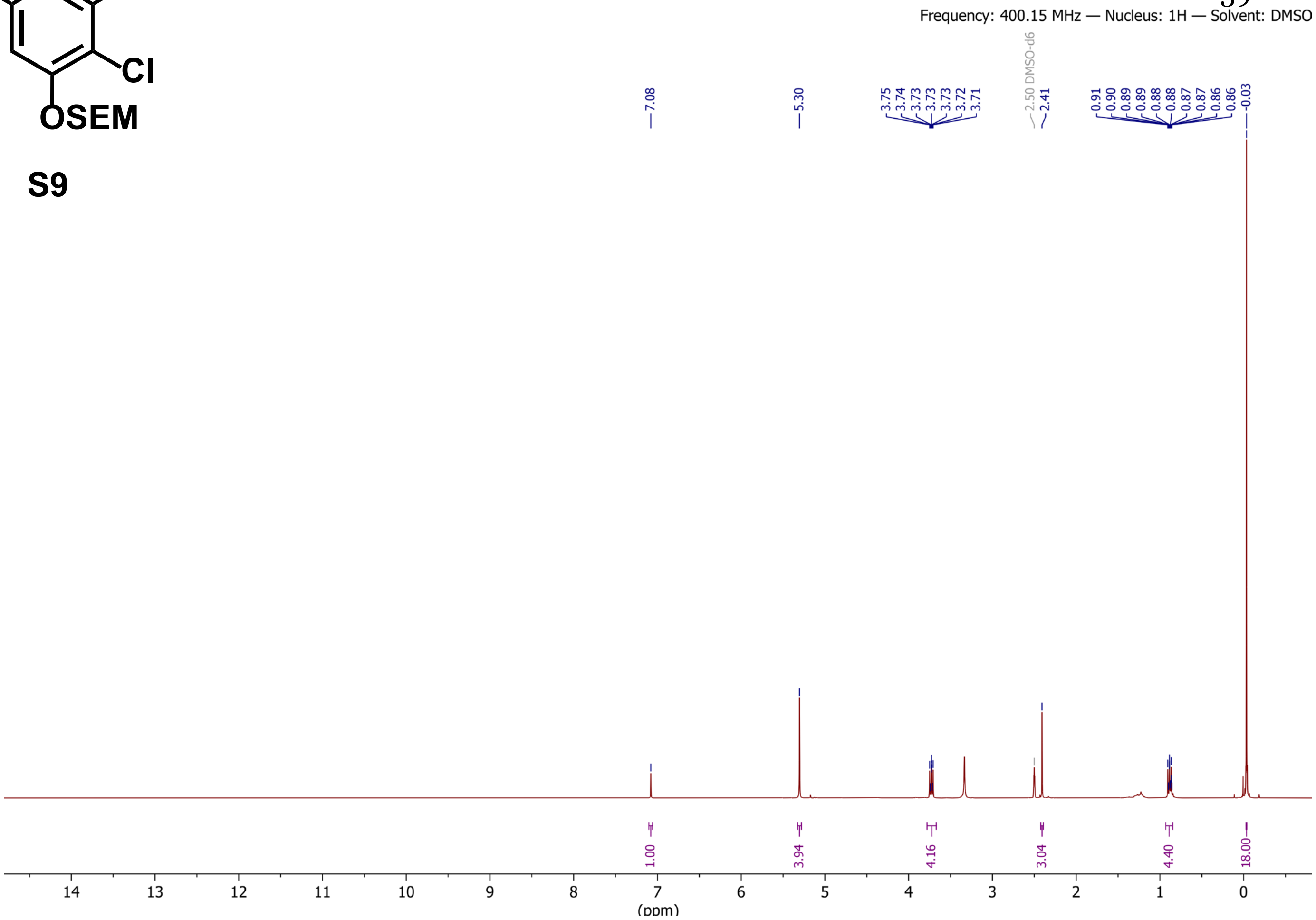
Frequency: 400.15 MHz — Nucleus: 1H — Solvent: DMSO
7.08
5.30
3.75
3.74
3.73
3.73
3.73
3.72
3.71
2.50 DMSO-d6
2.41
0.91
0.90
0.89
0.89
0.88
0.88
0.87
0.87
0.86
0.86
-0.03
1.00
3.94
4.16
3.04
4.40
18.00
(ppm)

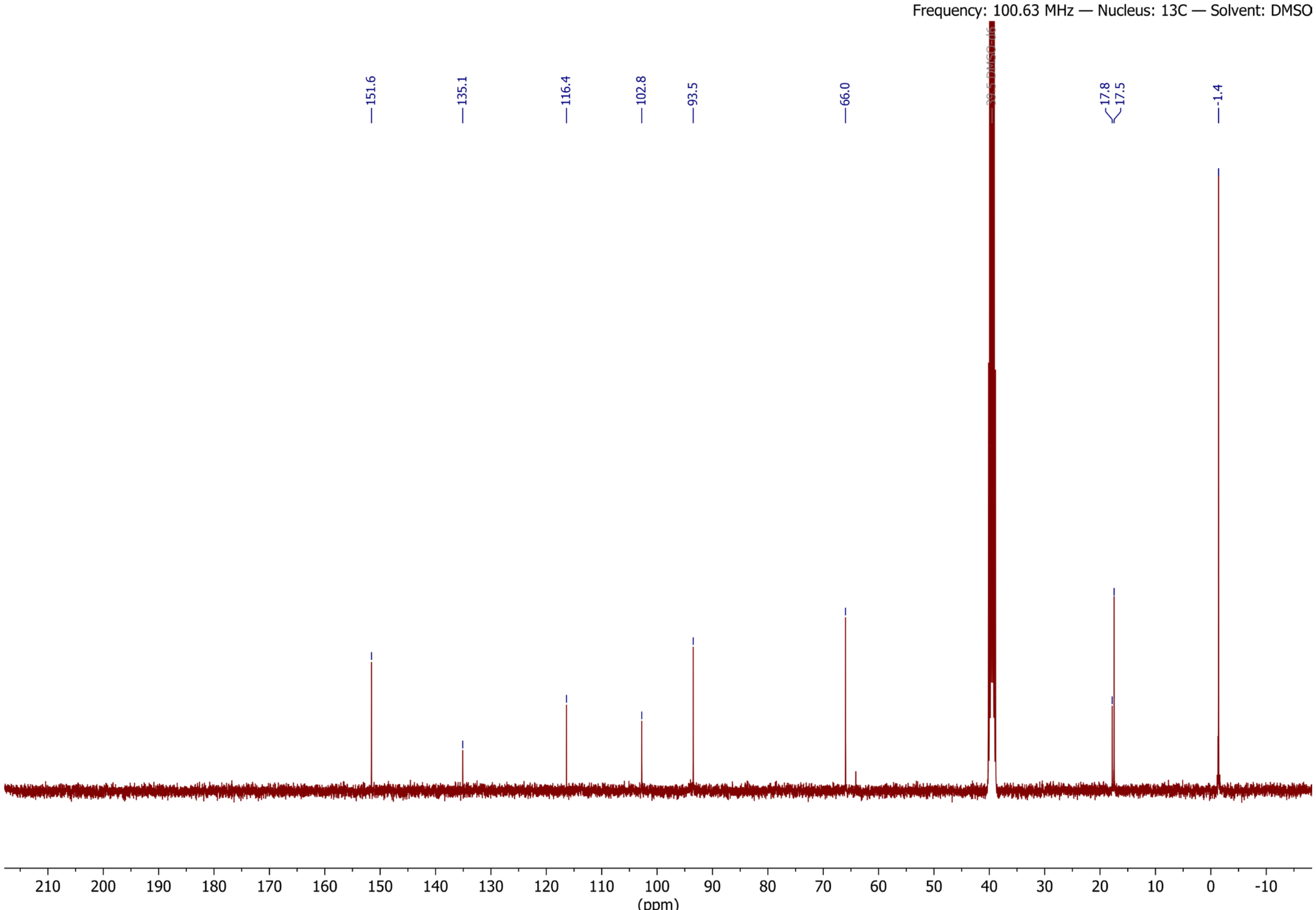
Frequency: 100.63 MHz — Nucleus: 13C — Solvent: DMSO
151.6
135.1
116.4
102.8
93.5
66.0
17.8
17.5
-1.4
(ppm)

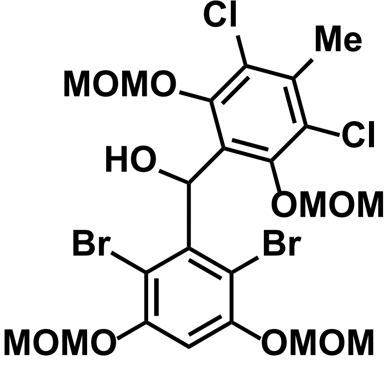

19

Frequency: 400.15 MHz — Nucleus: 1H — Solvent: DMSO

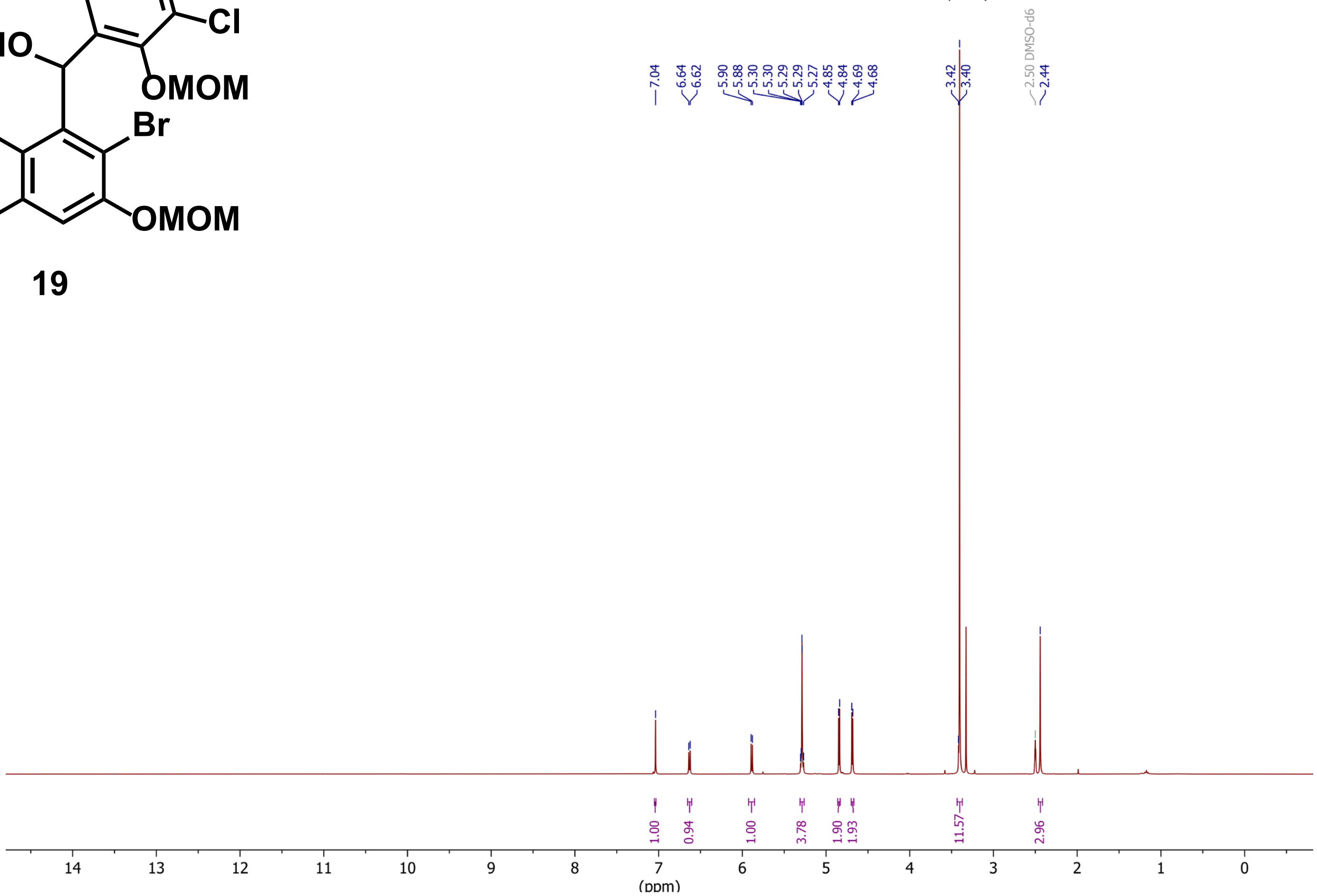

Frequency: 100.63 MHz — Nucleus: 13C — Solvent: DMSO

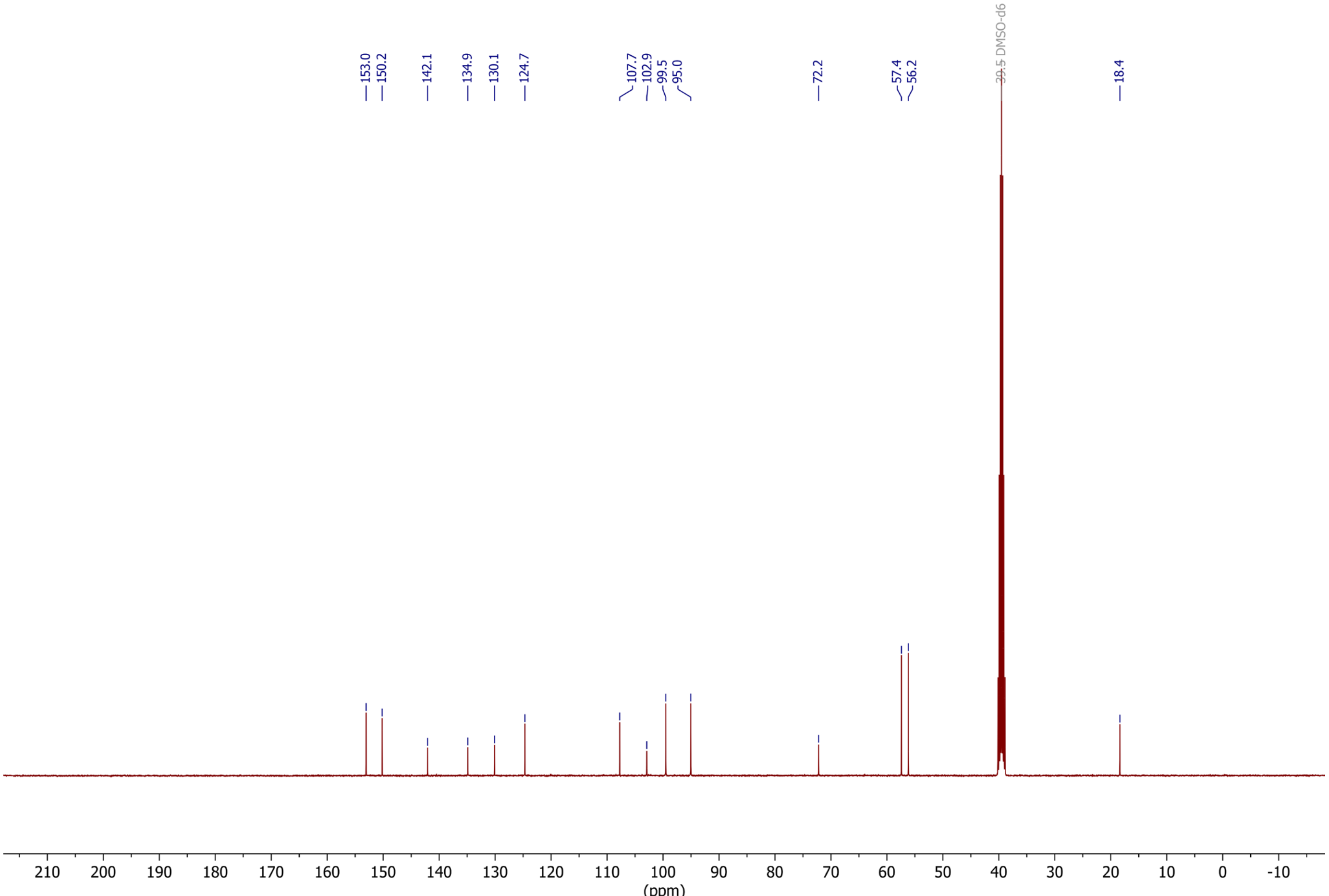

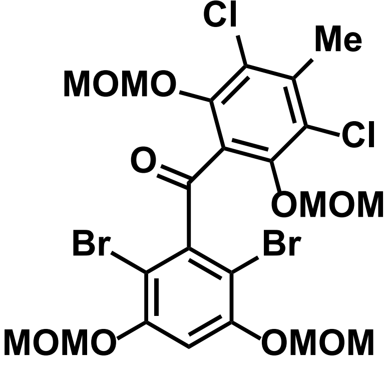

21

Frequency: 400.15 MHz — Nucleus: 1H — Solvent: DMSO

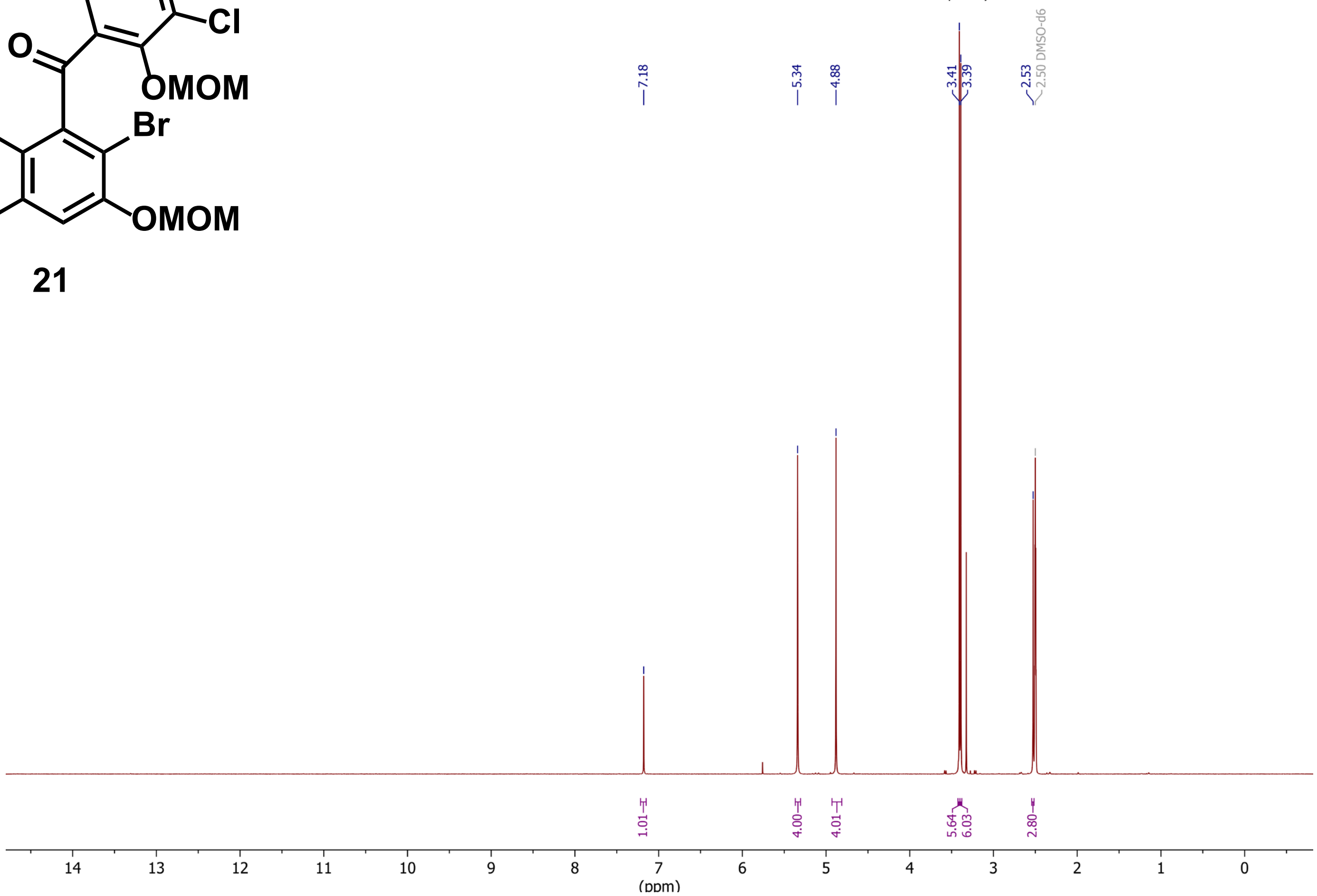

Frequency: 100.63 MHz — Nucleus: 13C — Solvent: DMSO

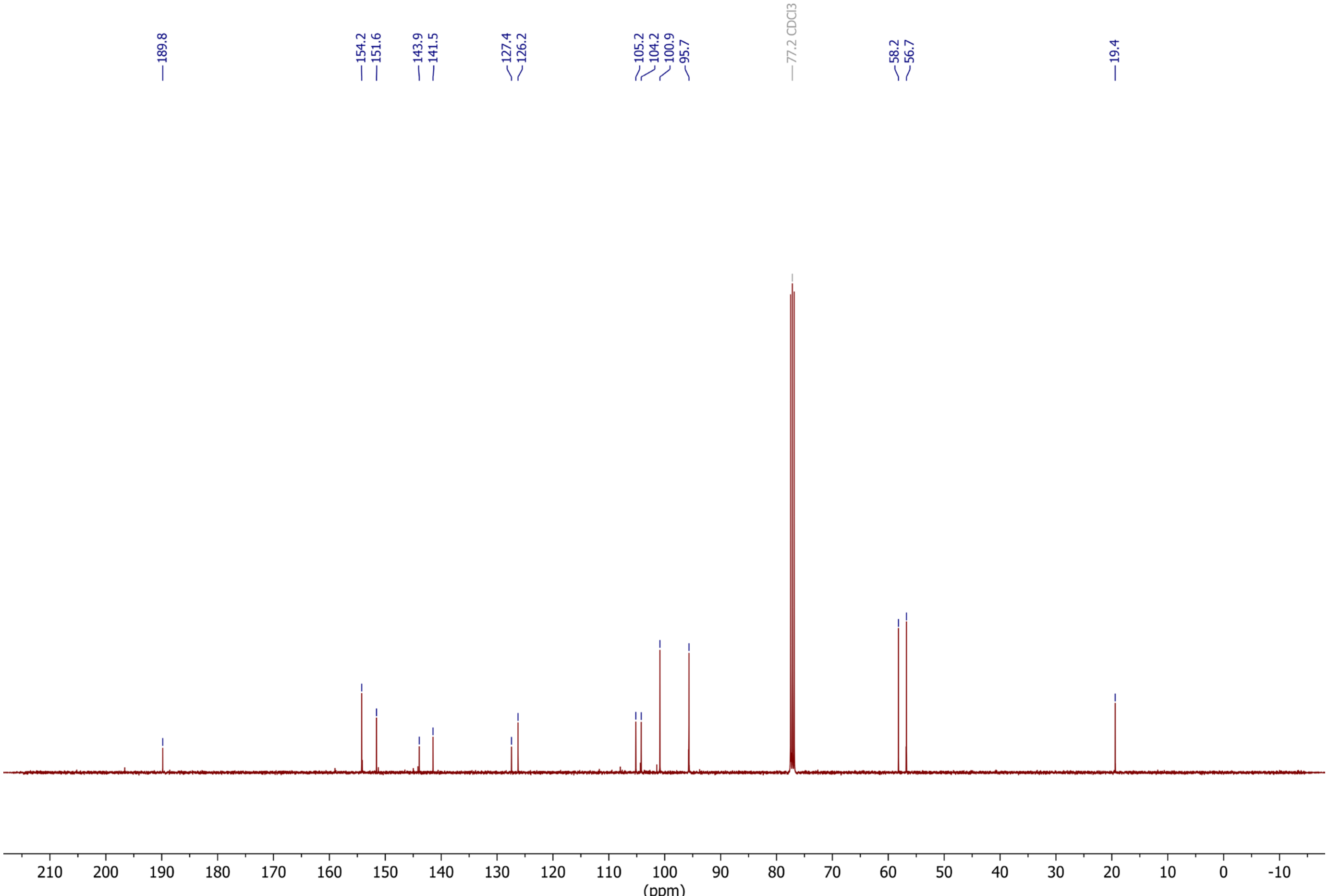

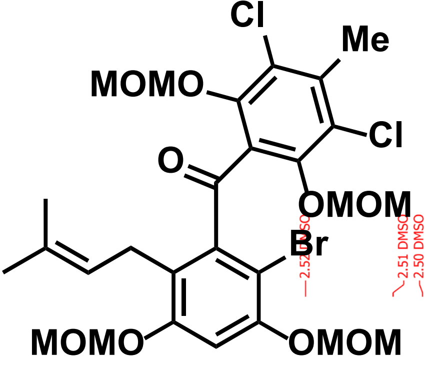

22

Frequency: 400.15 MHz — Nucleus: 1H — Solvent: DMSO

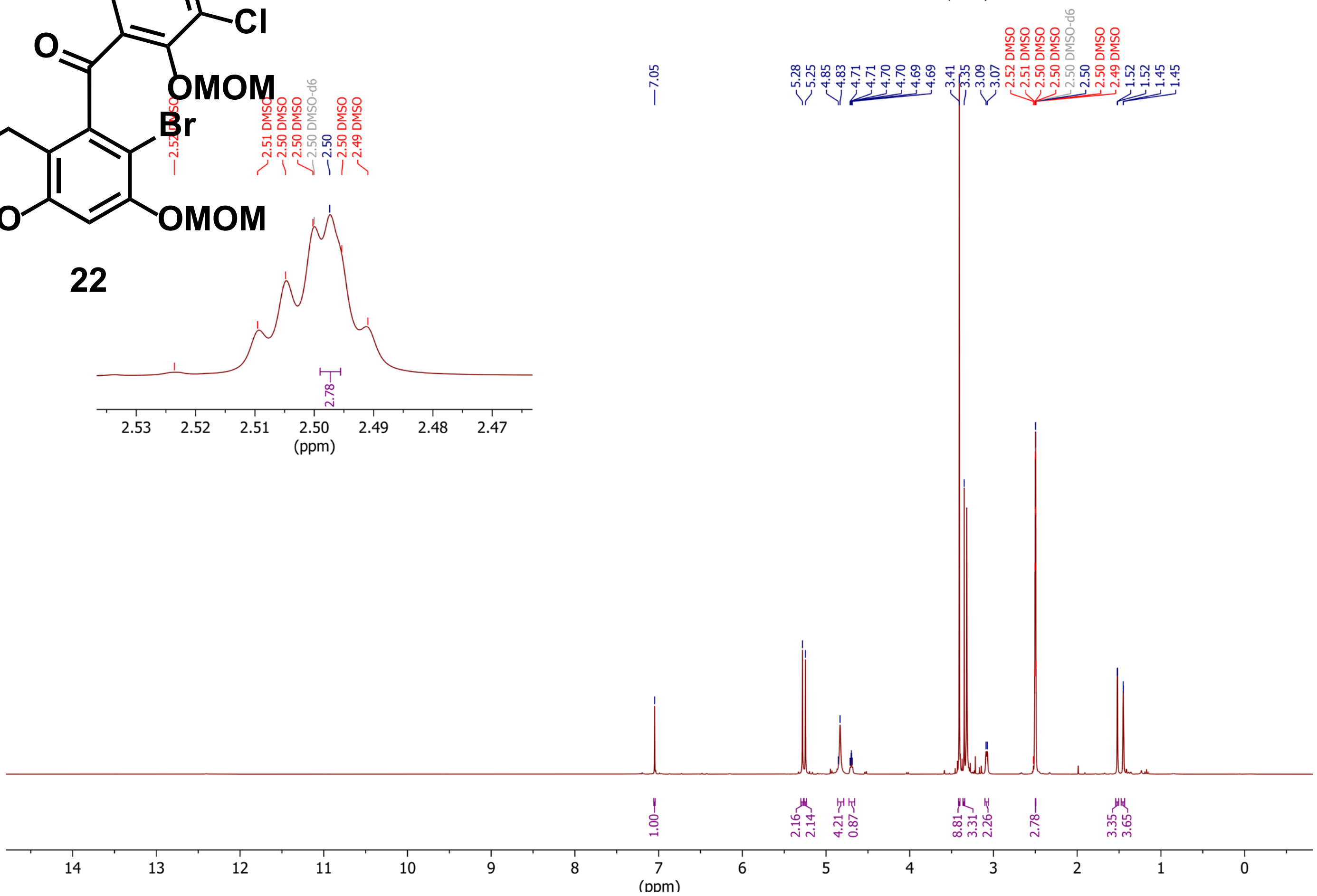

Frequency: 100.63 MHz — Nucleus: 13C — Solvent: DMSO

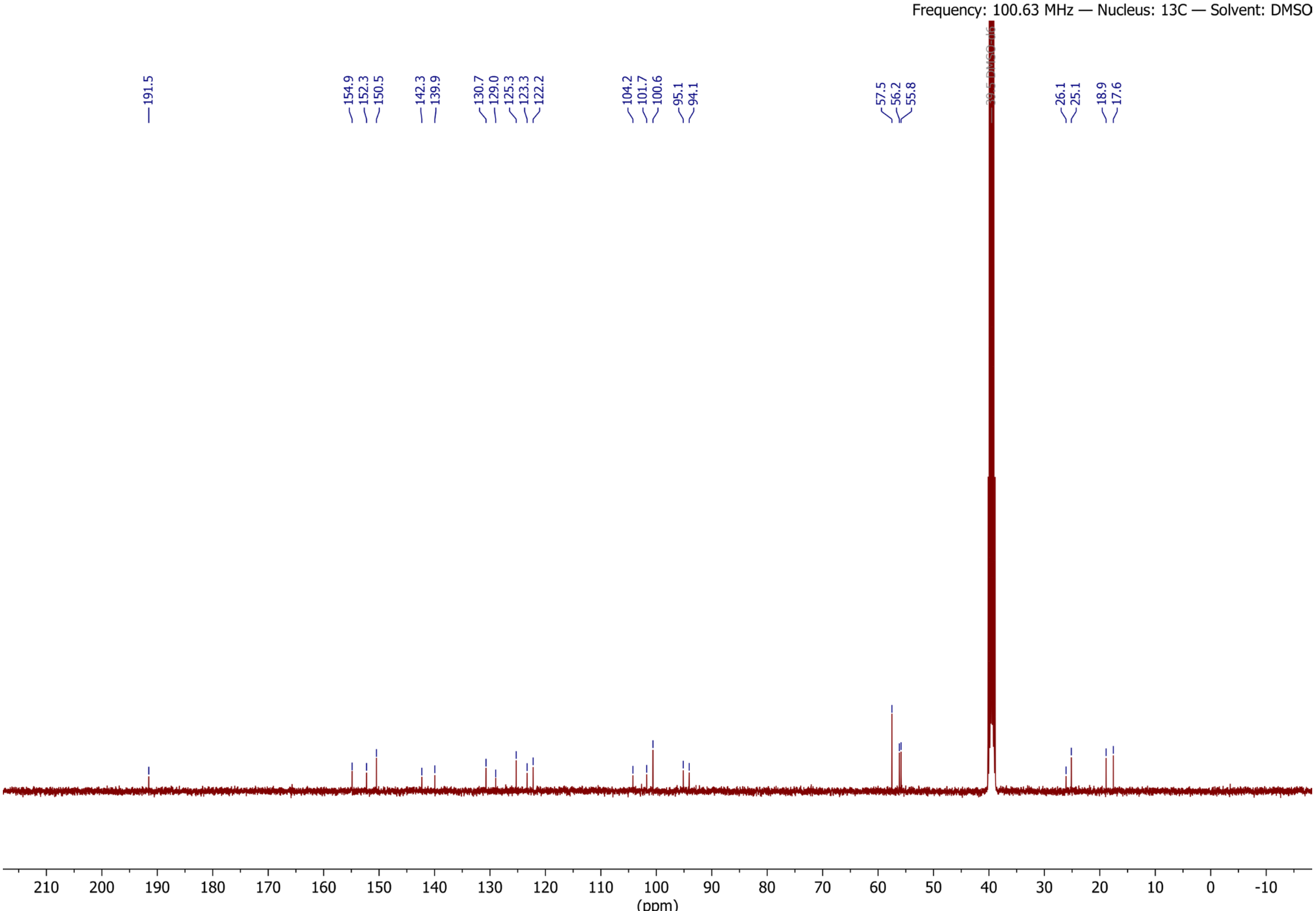

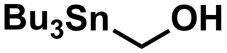
Bu3Sn
OH

S19

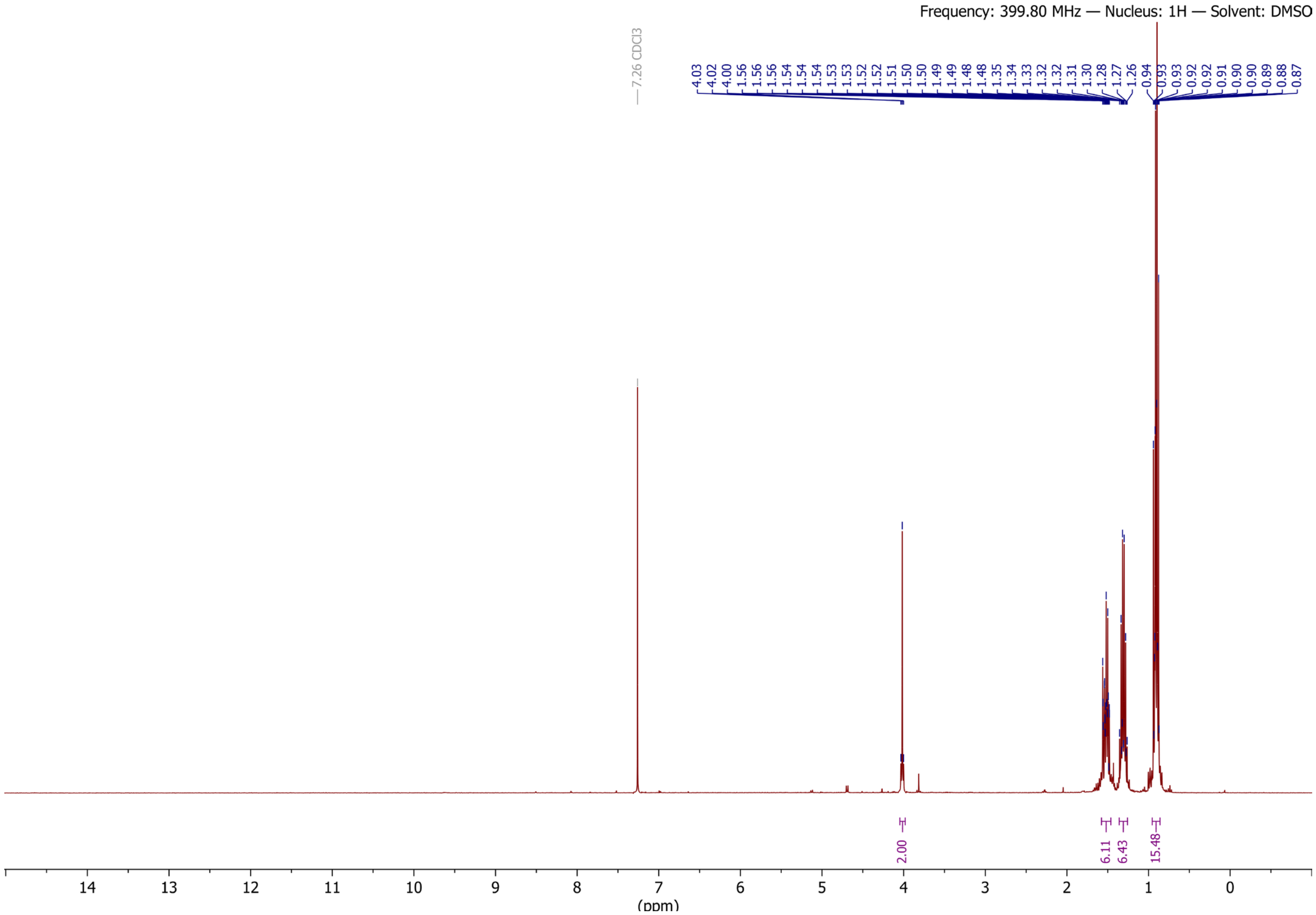
Frequency: 399.80 MHz — Nucleus: 1H — Solvent: DMSO
7.26 CDCl3
4.03
4.02
4.00
1.56
1.56
1.56
1.54
1.54
1.54
1.53
1.53
1.52
1.52
1.52
1.51
1.50
1.50
1.49
1.49
1.48
1.48
1.35
1.34
1.33
1.32
1.32
1.31
1.30
1.28
1.27
1.26
0.94
0.93
0.93
0.92
0.92
0.91
0.90
0.90
0.89
0.88
0.87
2.00
6.11
6.43
15.48
14
13
12
11
10
9
8
7
6
5
4
3
2
1
0
(ppm)

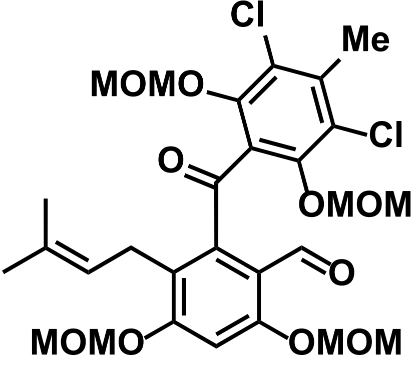

23

Frequency: 399.80 MHz — Nucleus: 1H — Solvent: DMSO

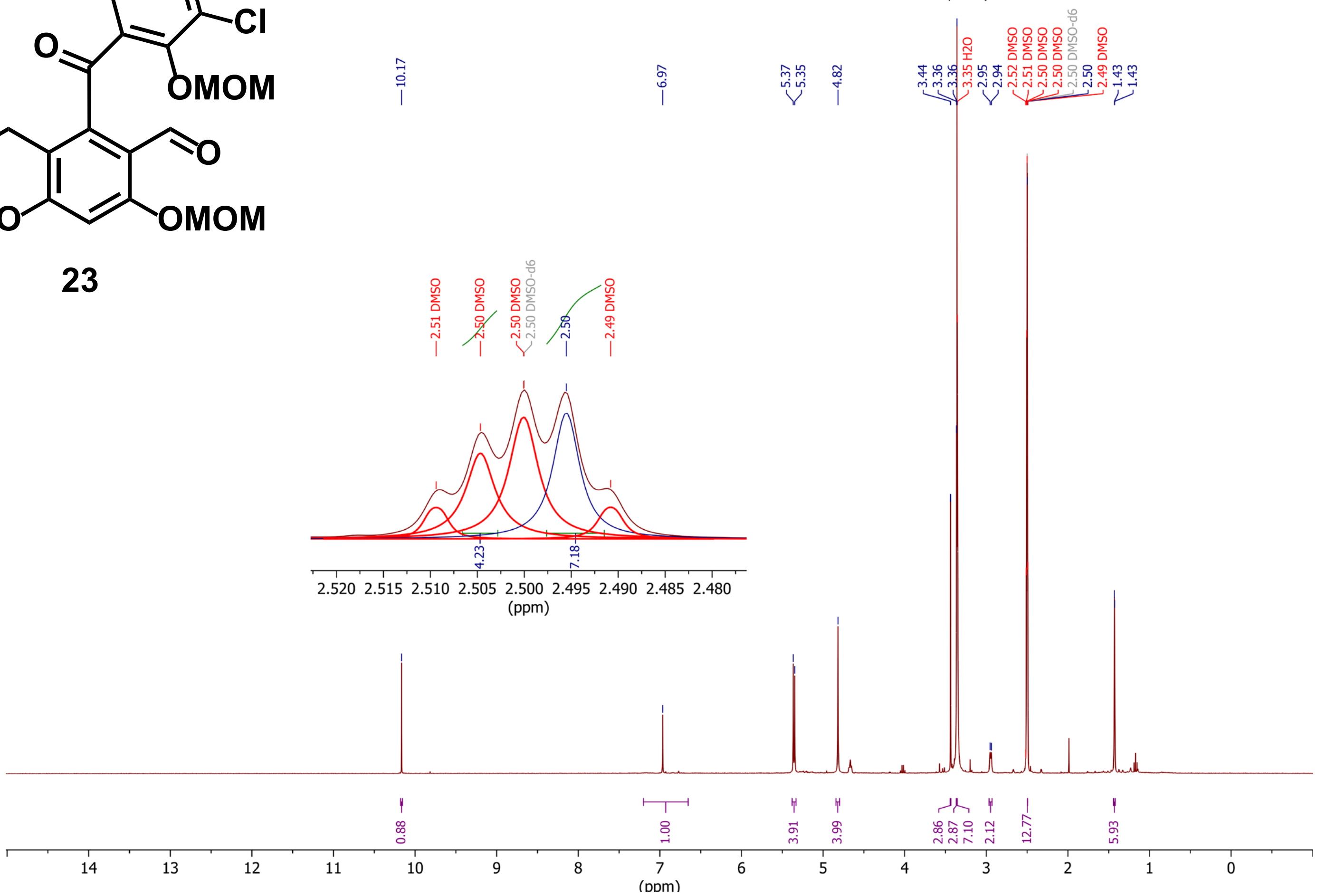

Frequency: 201.25 MHz — Nucleus: 13C — Solvent: DMSO

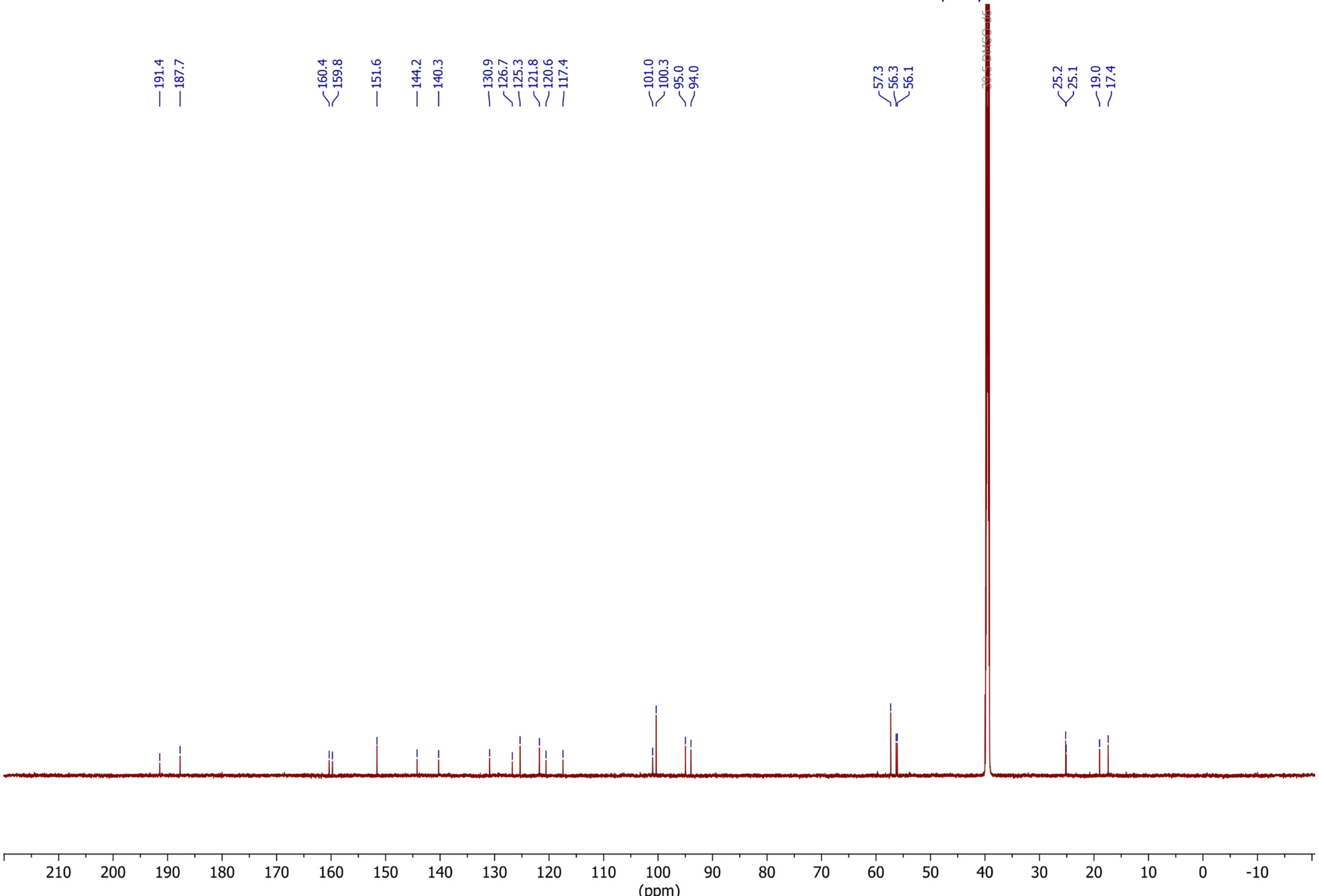

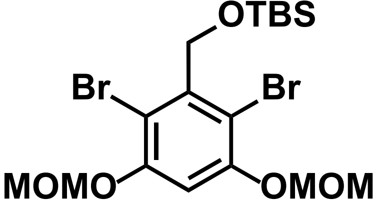

25

Frequency: 400.15 MHz — Nucleus: 1H — Solvent: DMSO

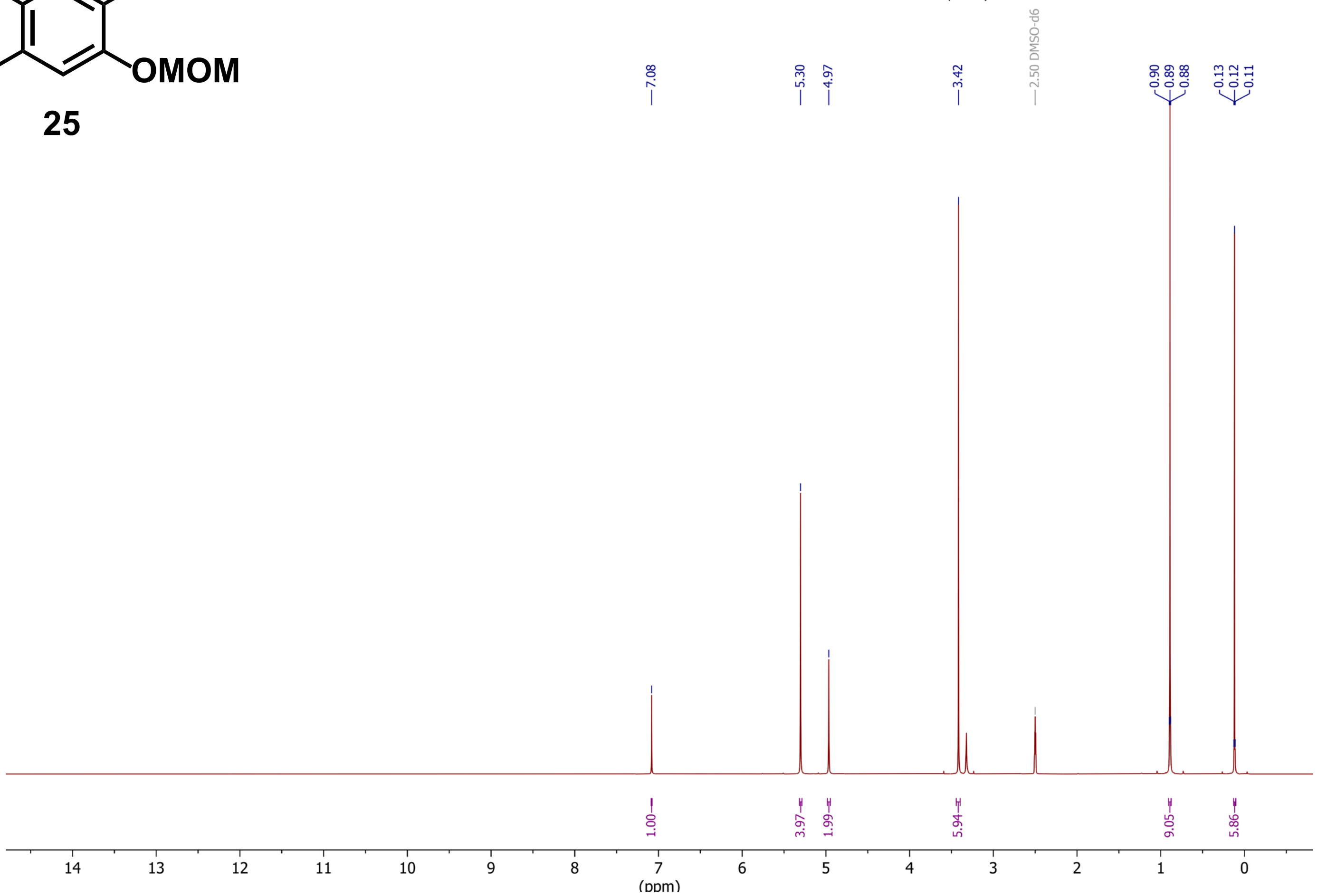

Frequency: 100.63 MHz — Nucleus: 13C — Solvent: DMSO

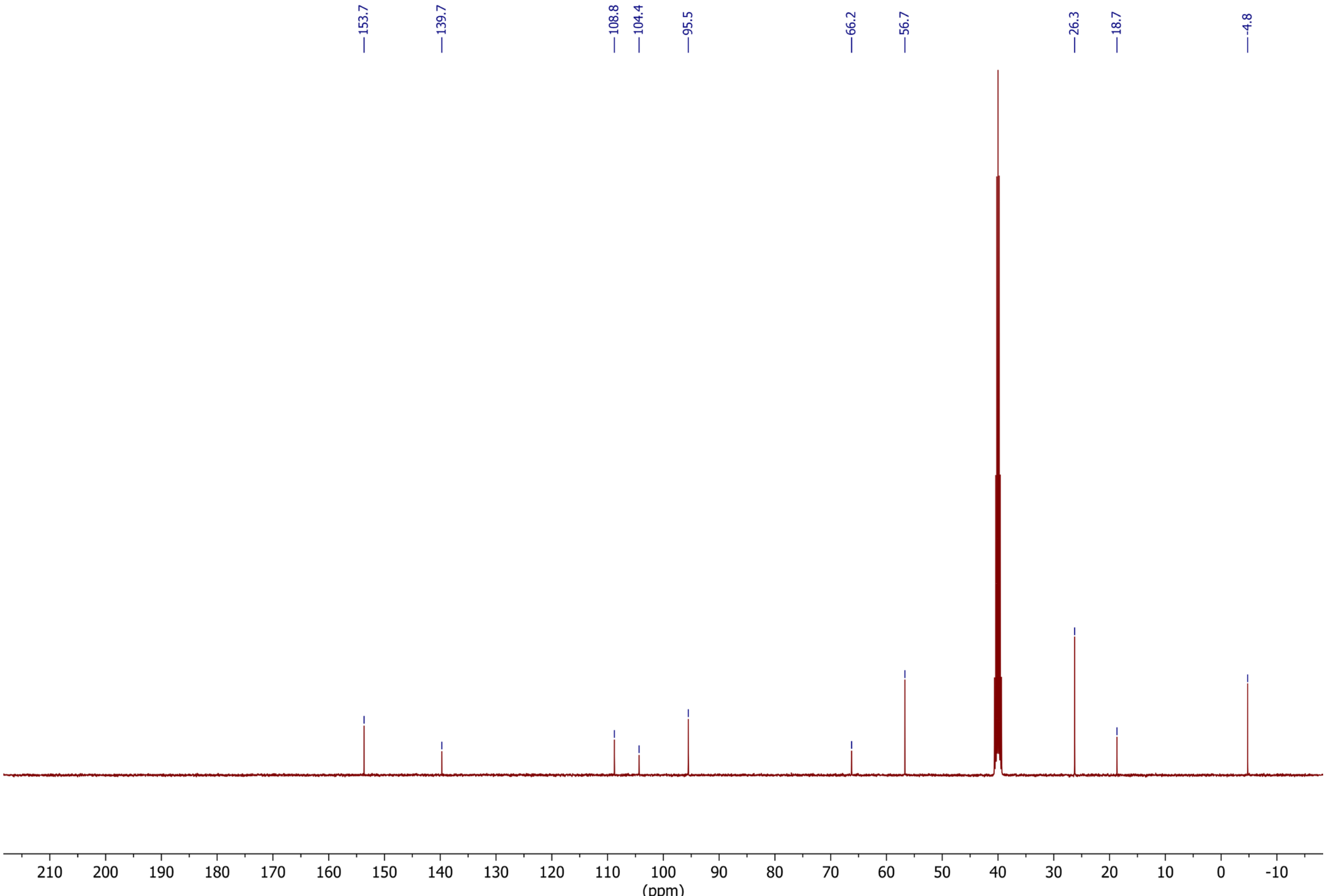

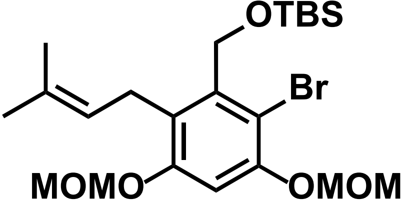
OTBS
Br
MOMO
OMOM

26

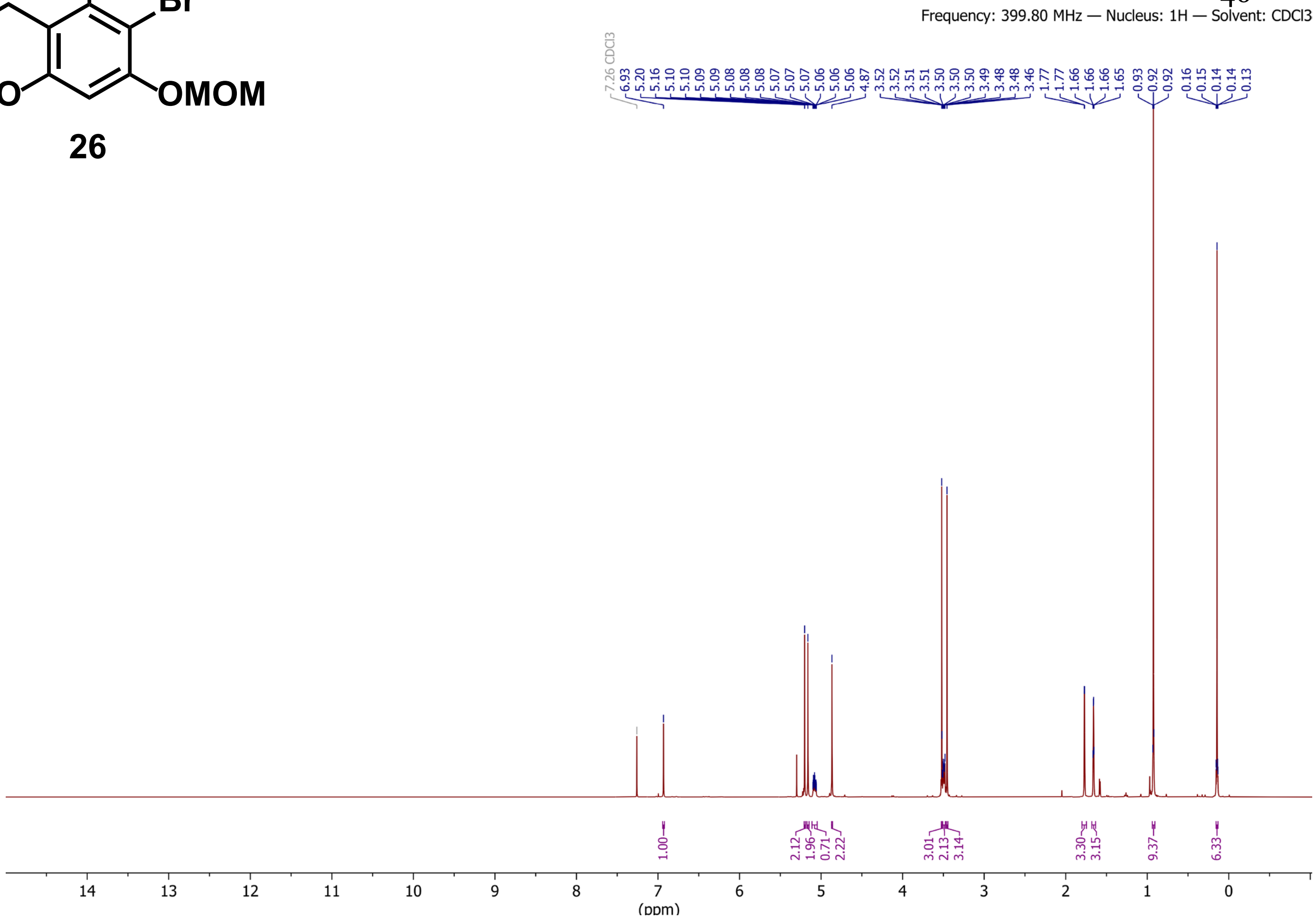
Frequency: 399.80 MHz — Nucleus: 1H — Solvent: CDCl3
7.26 CDCl3
6.93
5.20
5.16
5.10
5.09
5.09
5.08
5.08
5.08
5.07
5.07
5.07
5.06
5.06
5.06
4.87
3.52
3.52
3.51
3.51
3.50
3.50
3.50
3.49
3.48
3.48
3.46
1.77
1.77
1.66
1.66
1.66
1.65
0.93
0.92
0.92
0.16
0.15
0.14
0.14
0.13
1.00
2.12
1.96
0.71
2.22
3.01
2.13
3.14
3.30
3.15
9.37
6.33
(ppm)

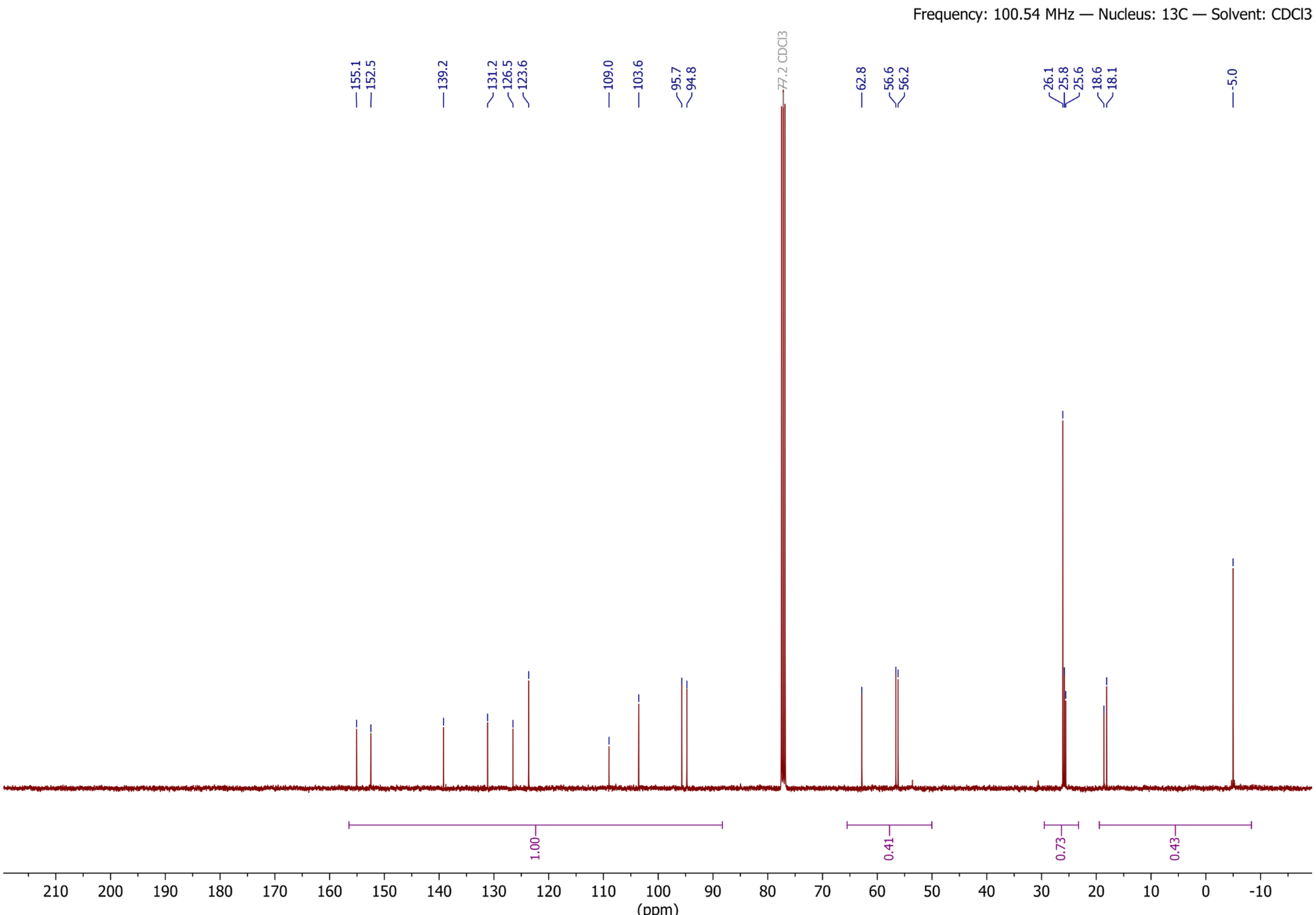
Frequency: 100.54 MHz — Nucleus: 13C — Solvent: CDCl3
155.1
152.5
139.2
131.2
126.5
123.6
109.0
103.6
95.7
94.8
77.2 CDCl3
62.8
56.6
56.2
26.1
25.8
25.6
18.6
18.1
-5.0
1.00
0.41
0.73
0.43
(ppm)

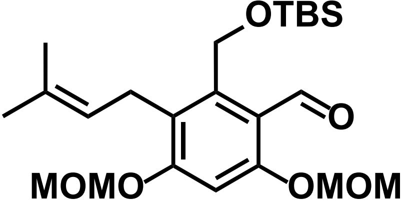

27

Frequency: 400.15 MHz — Nucleus: 1H — Solvent: CDCl3

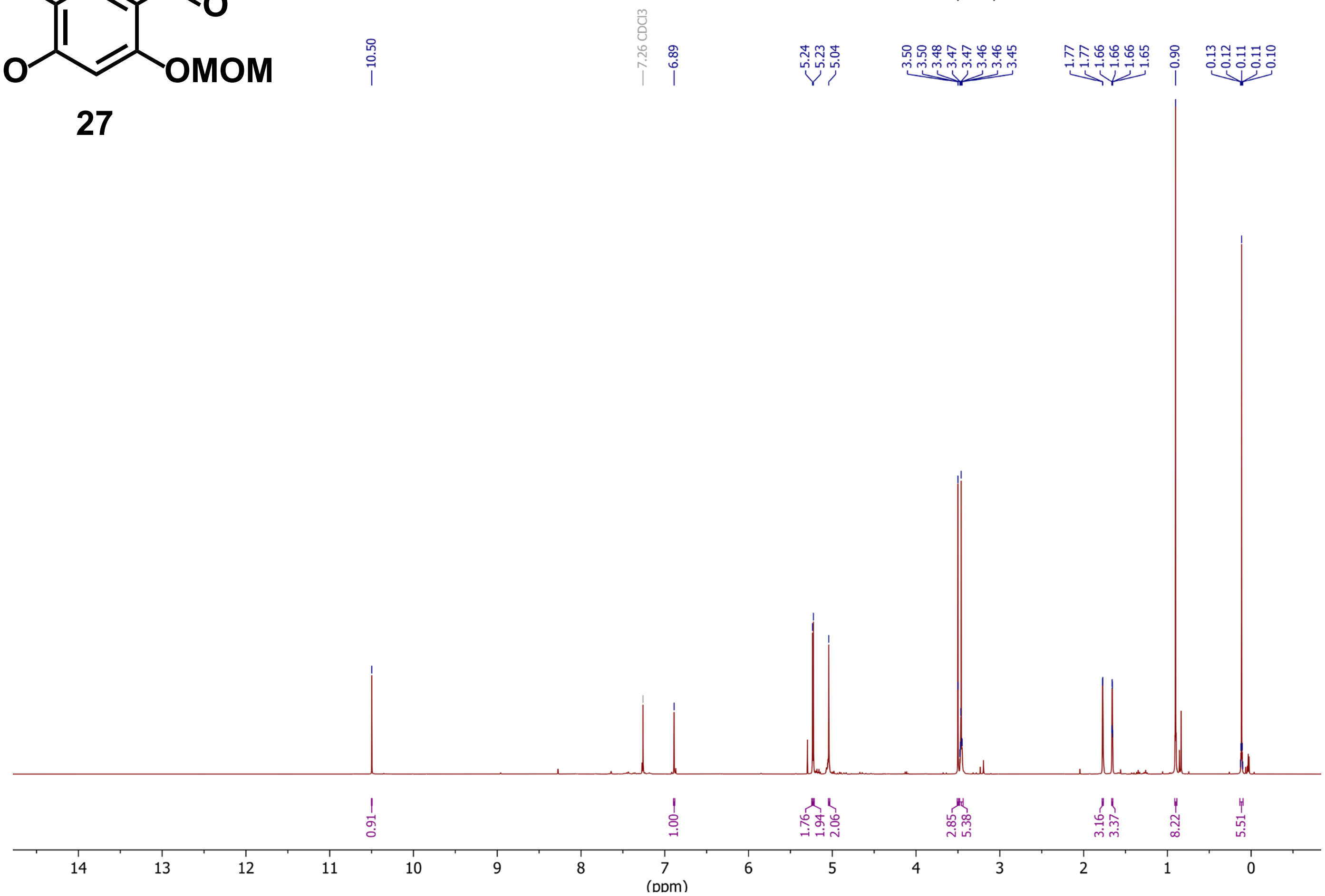

Frequency: 100.63 MHz — Nucleus: 13C — Solvent: CDCl3

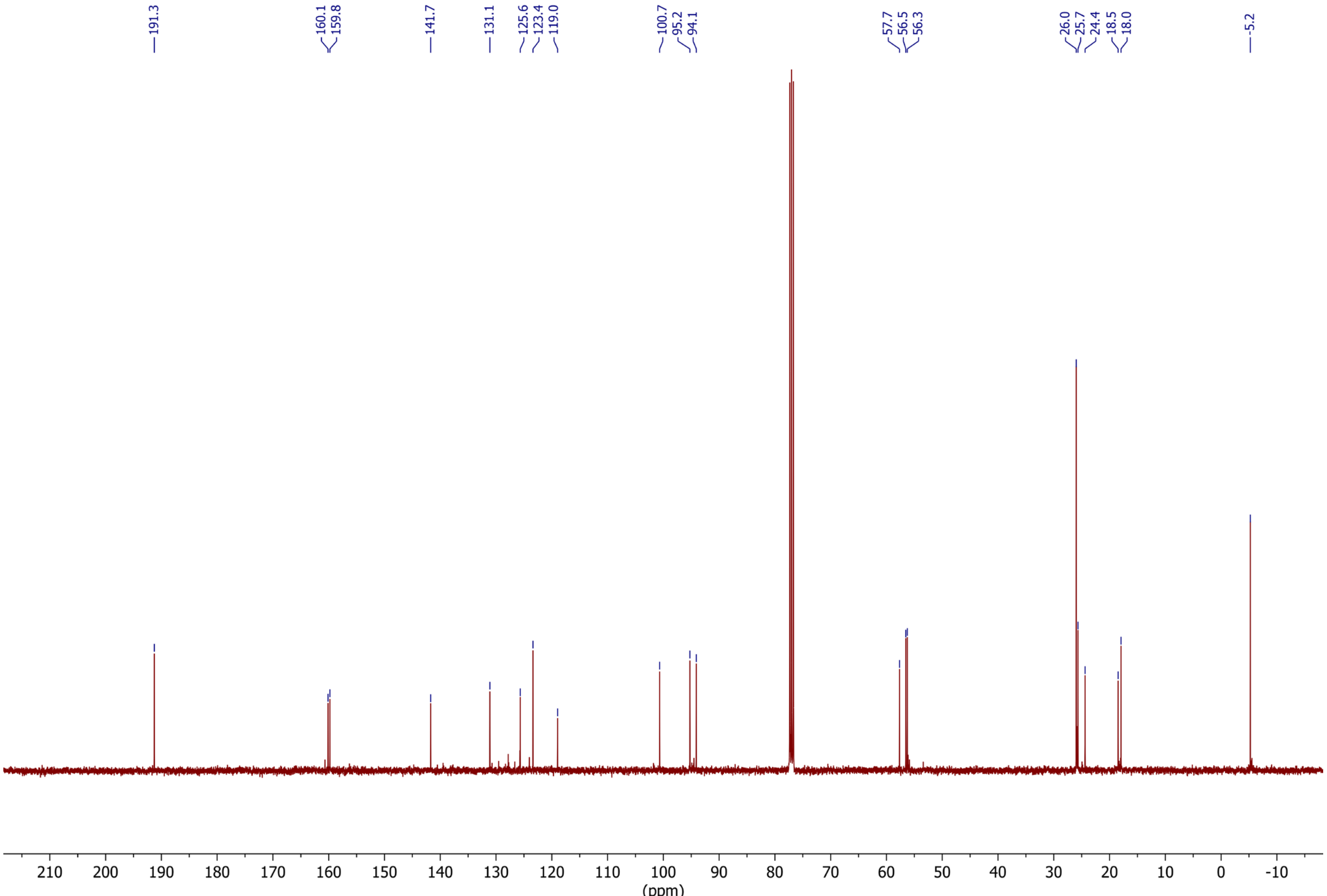

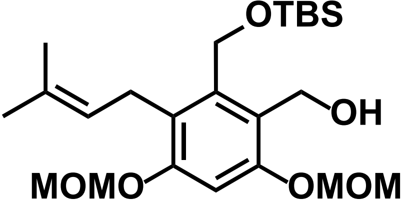

28

Frequency: 399.80 MHz — Nucleus: 1H — Solvent: CDCl3

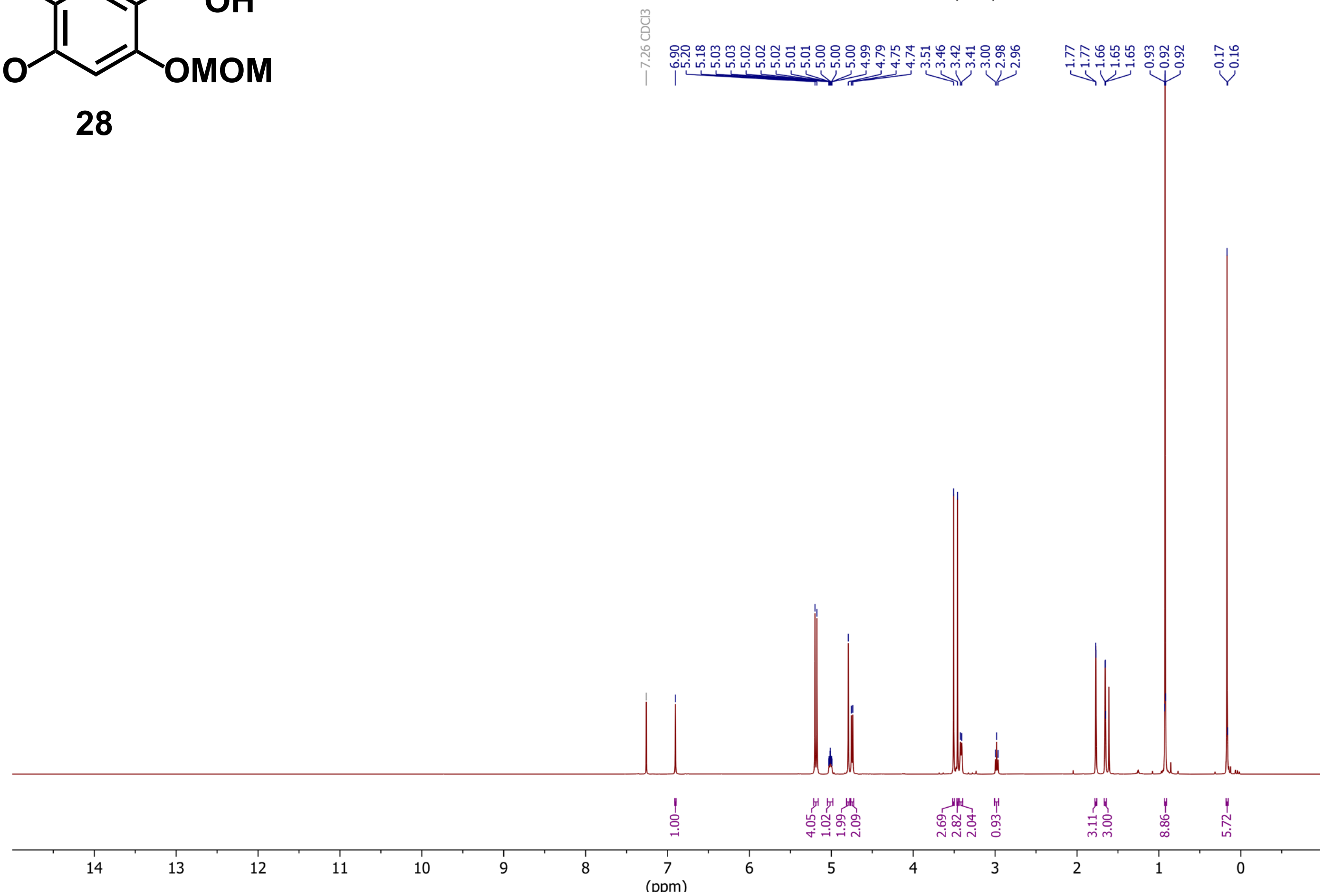

Frequency: 100.54 MHz — Nucleus: 13C — Solvent: CDCl3

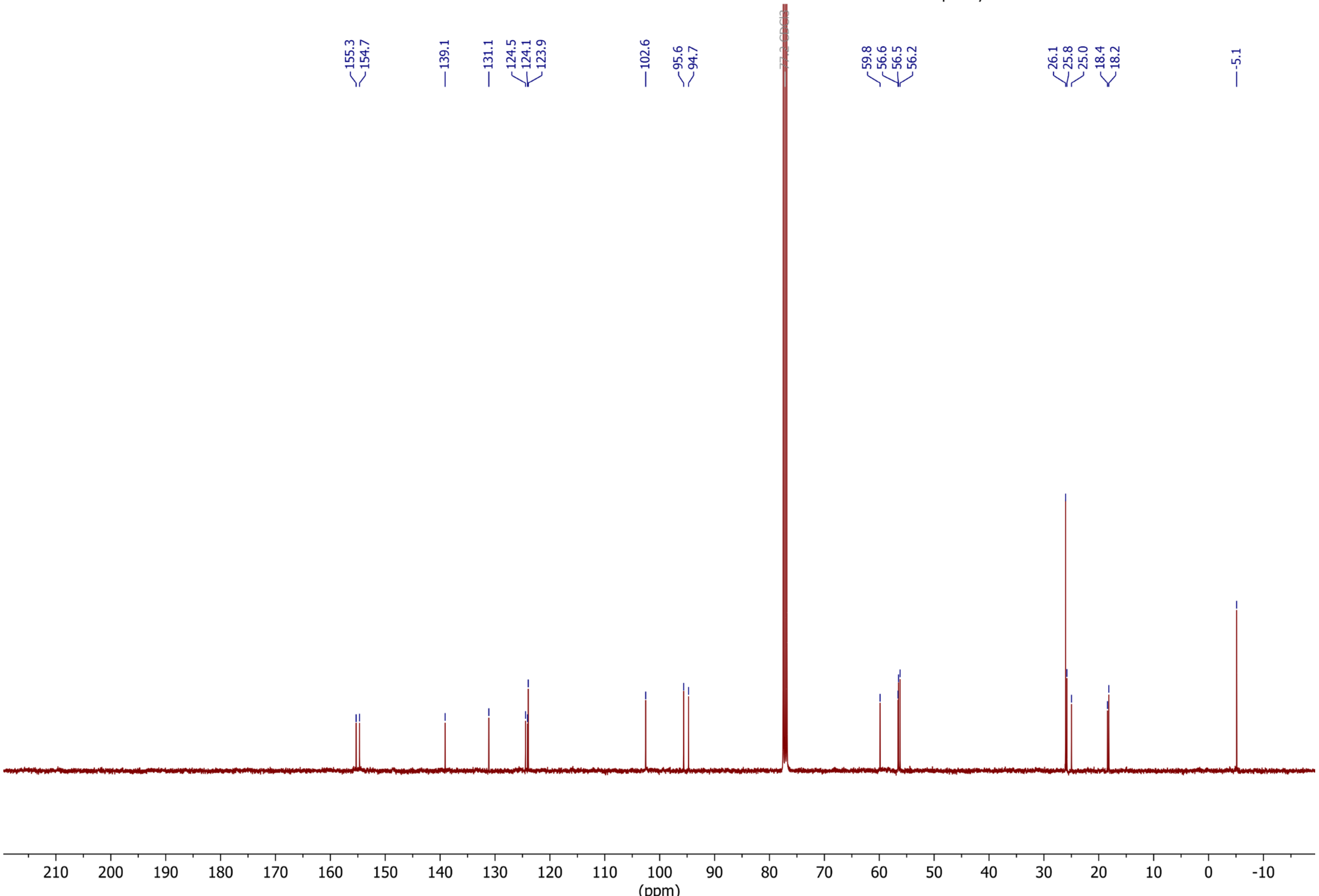

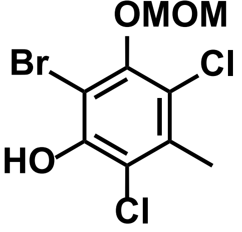

29

Frequency: 400.15 MHz — Nucleus: 1H — Solvent: DMSO

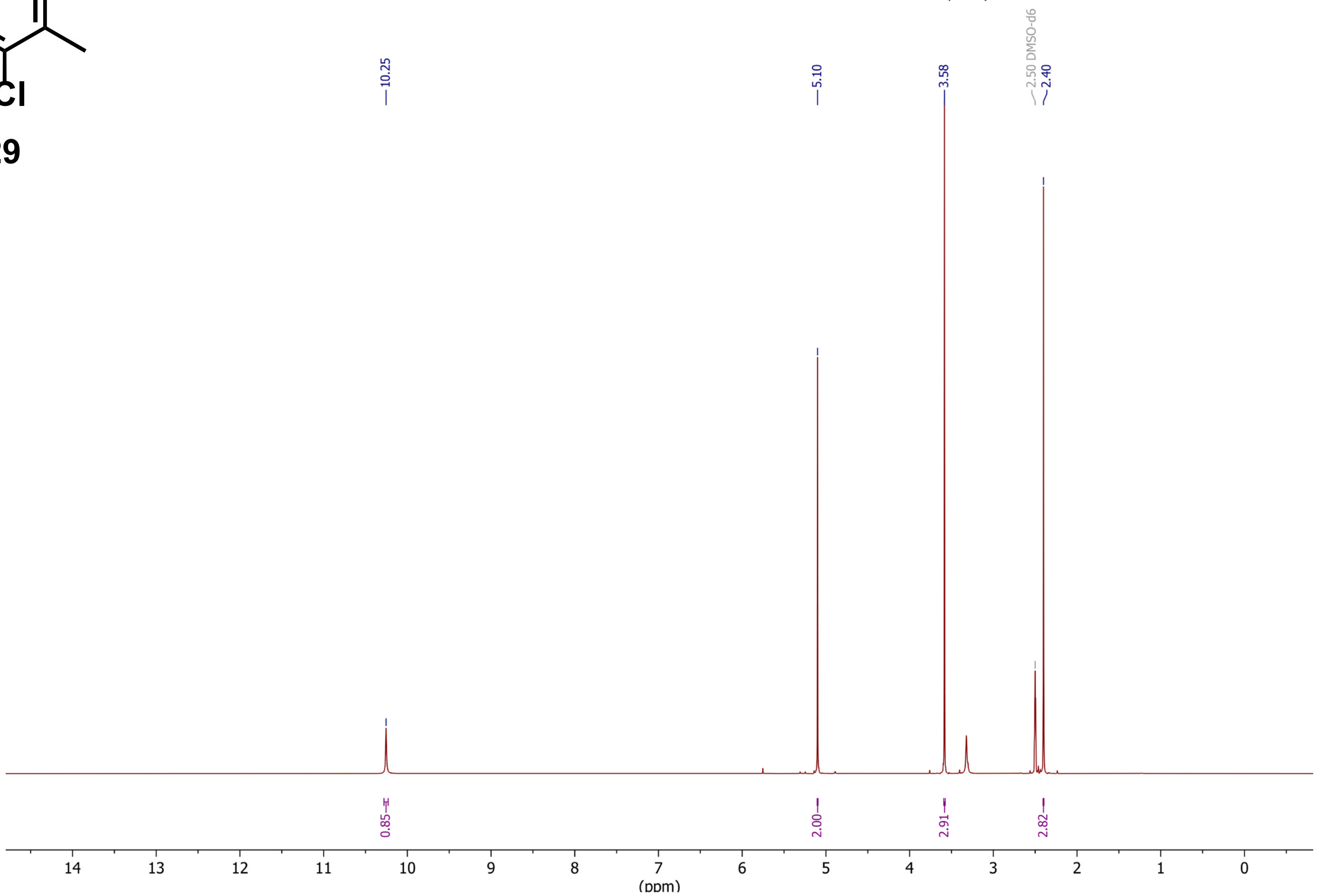

Frequency: 100.63 MHz — Nucleus: 13C — Solvent: DMSO

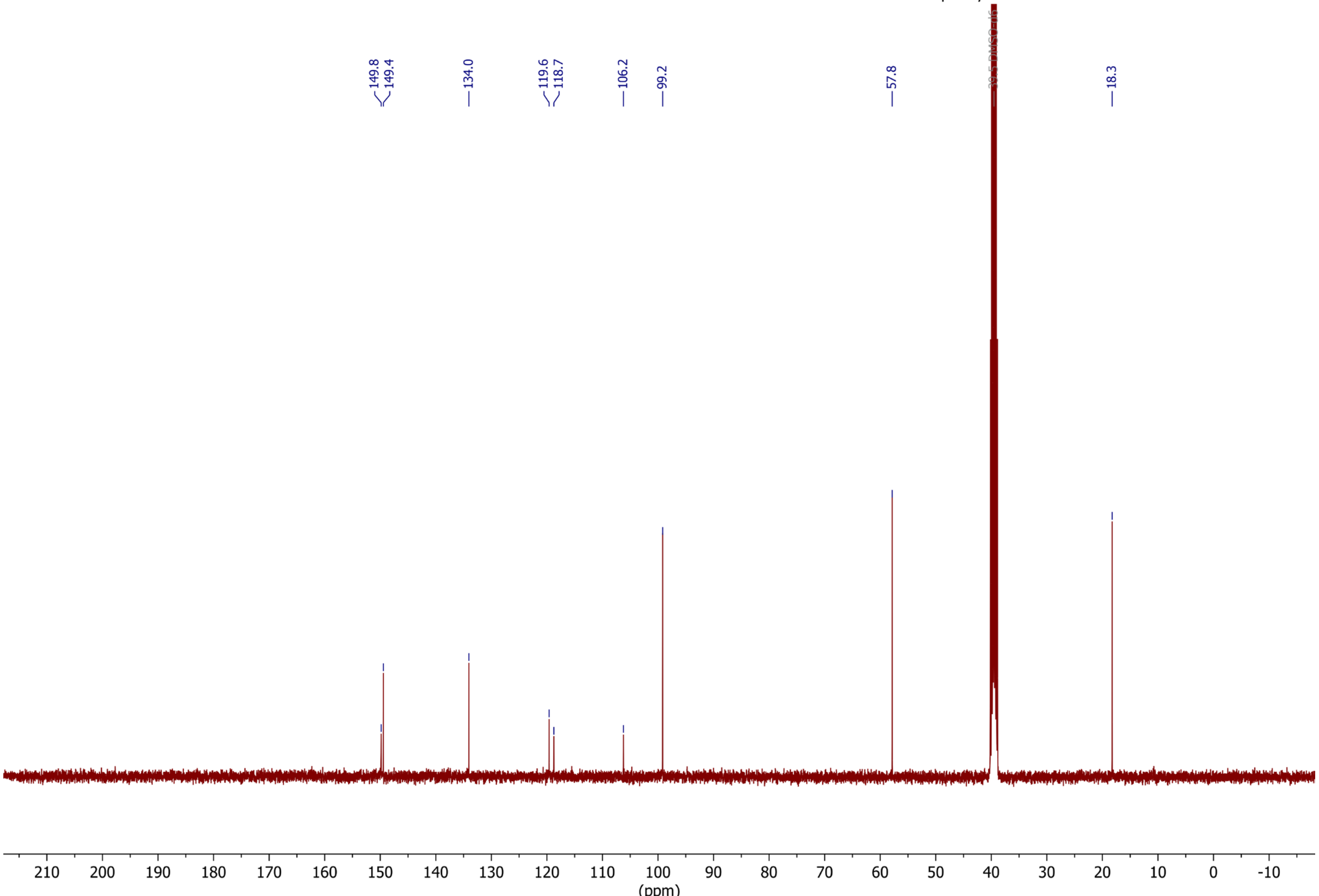

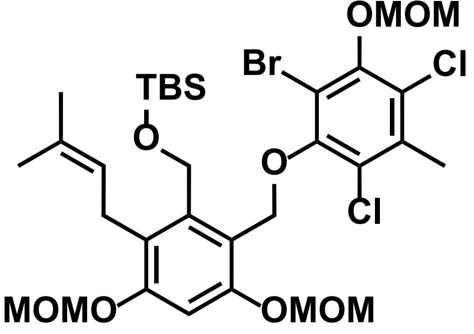

30

Frequency: 800.29 MHz — Nucleus: 1H — Solvent: CDCl3

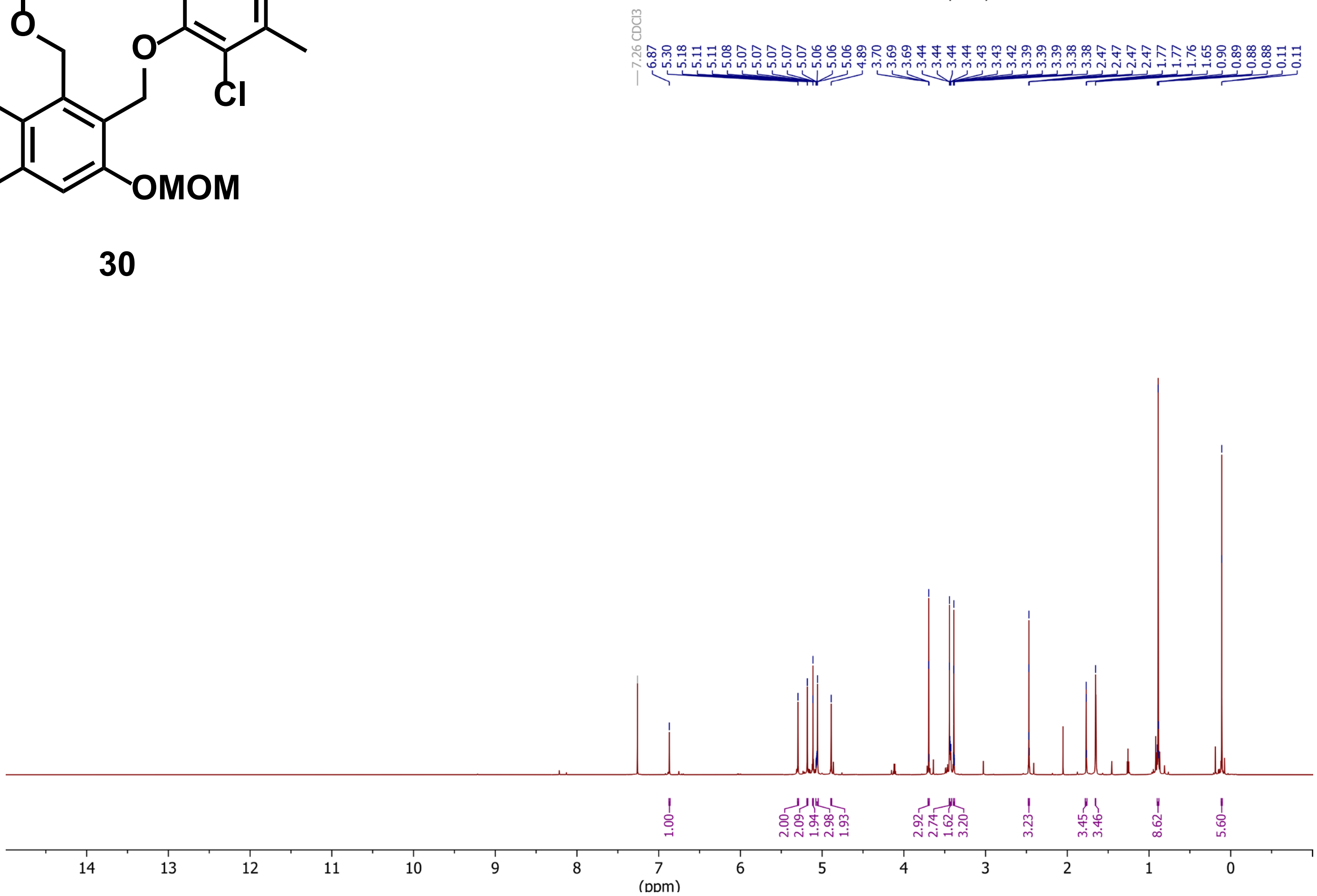

Frequency: 201.25 MHz — Nucleus: 13C — Solvent: CDCl3

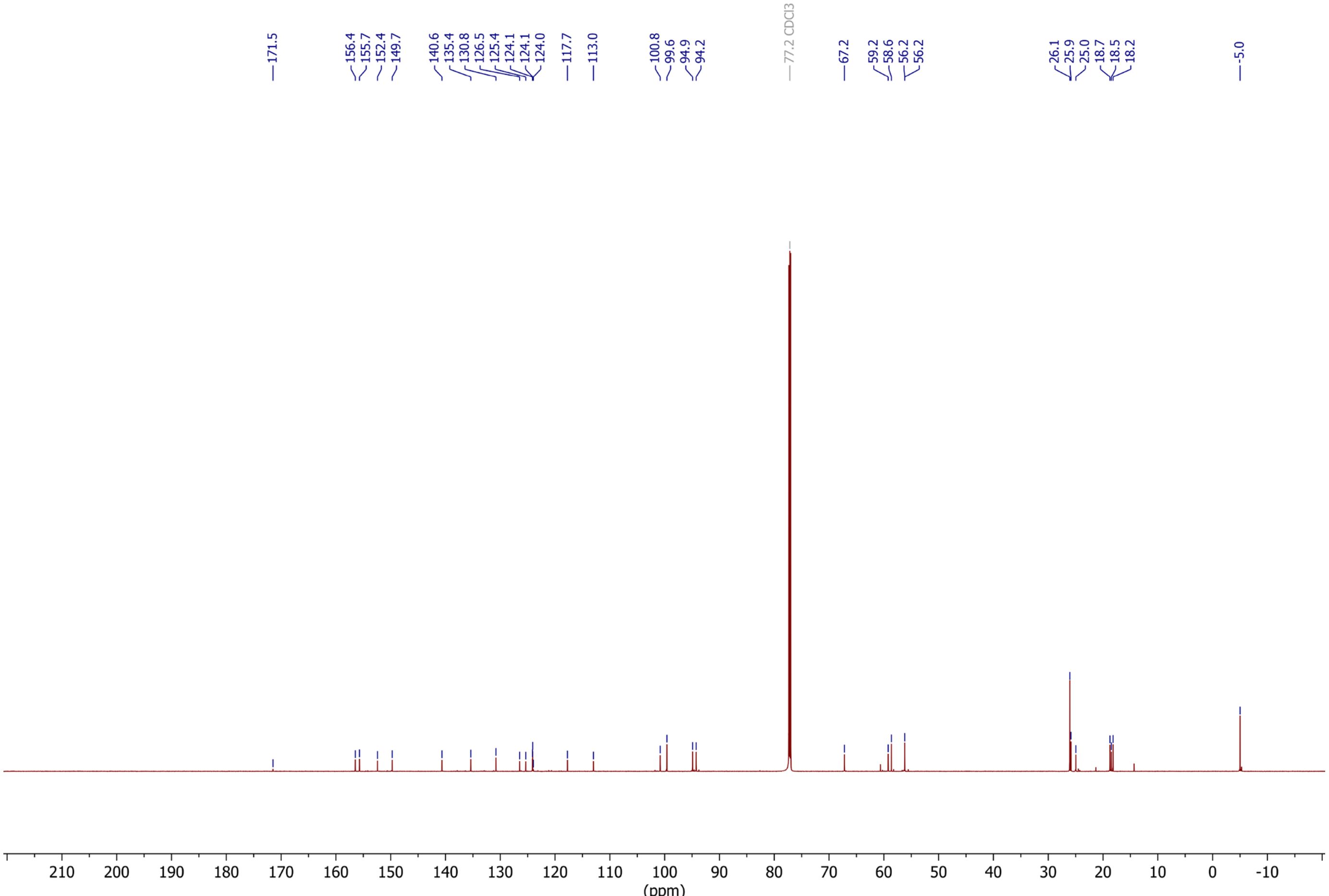

Experiment: HSQC — Frequency: 800.29 MHz — Nucleus: (1H, 13C) — Solvent: CDCl3

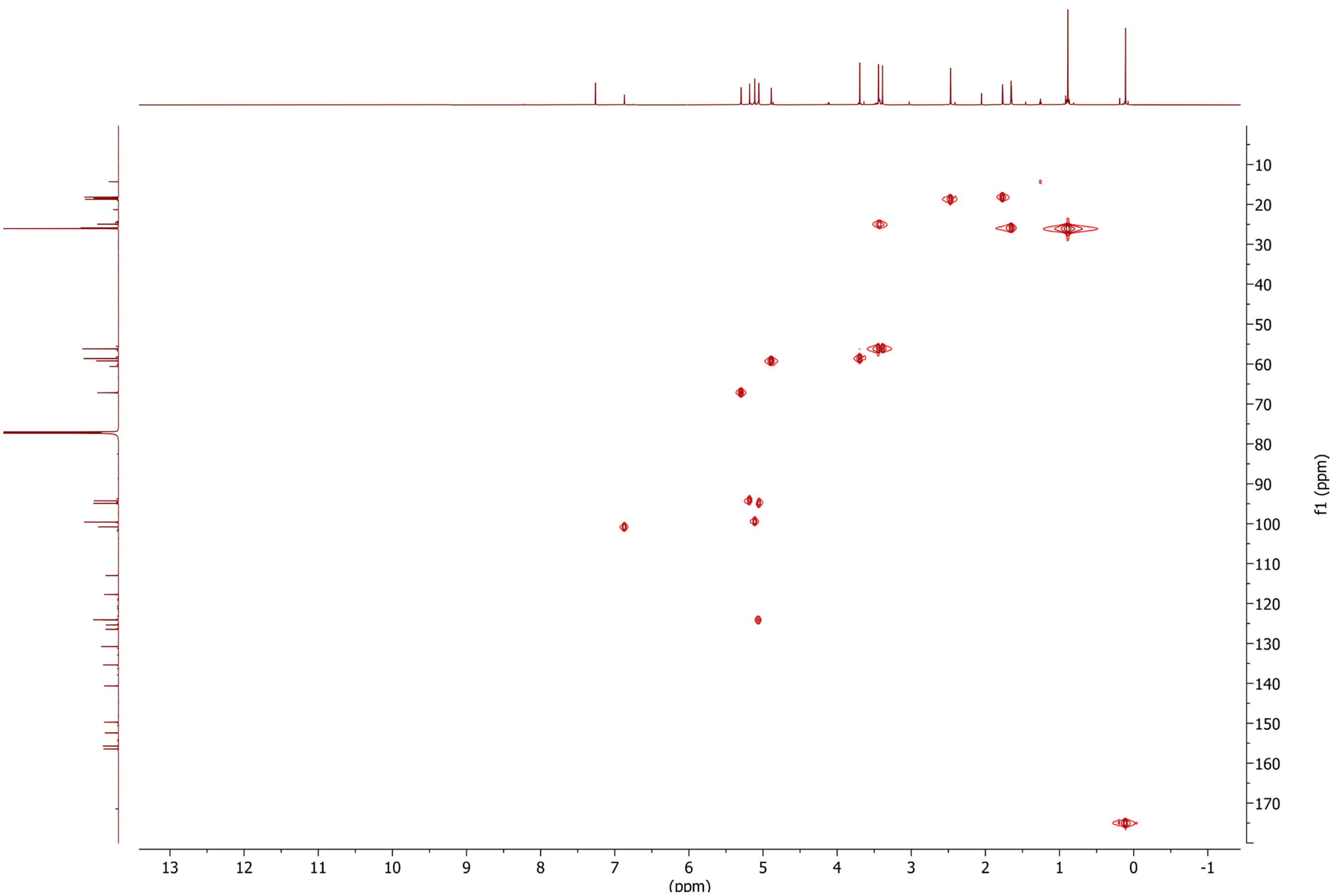

Experiment: HMBC — Frequency: 800.29 MHz — Nucleus: (1H, 13C) — Solvent: CDCl3

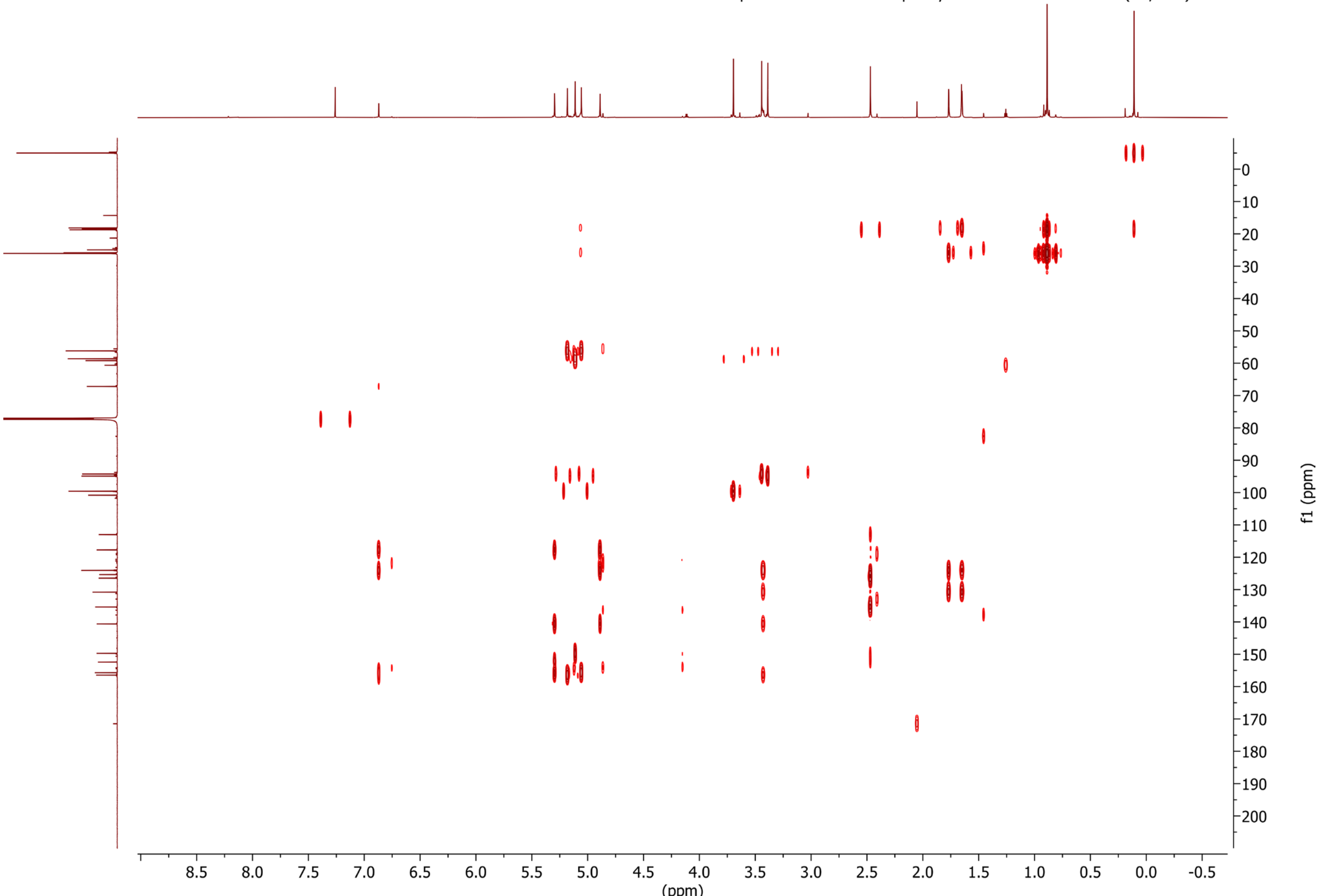

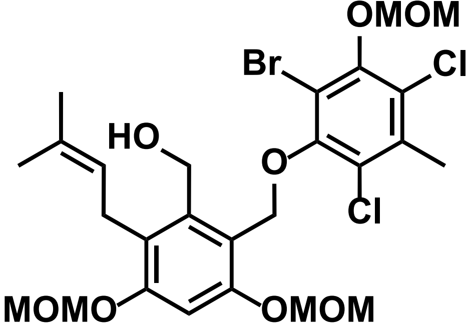
OMOM
Br
Cl
HO
O
Cl
MOMO
OMOM

31

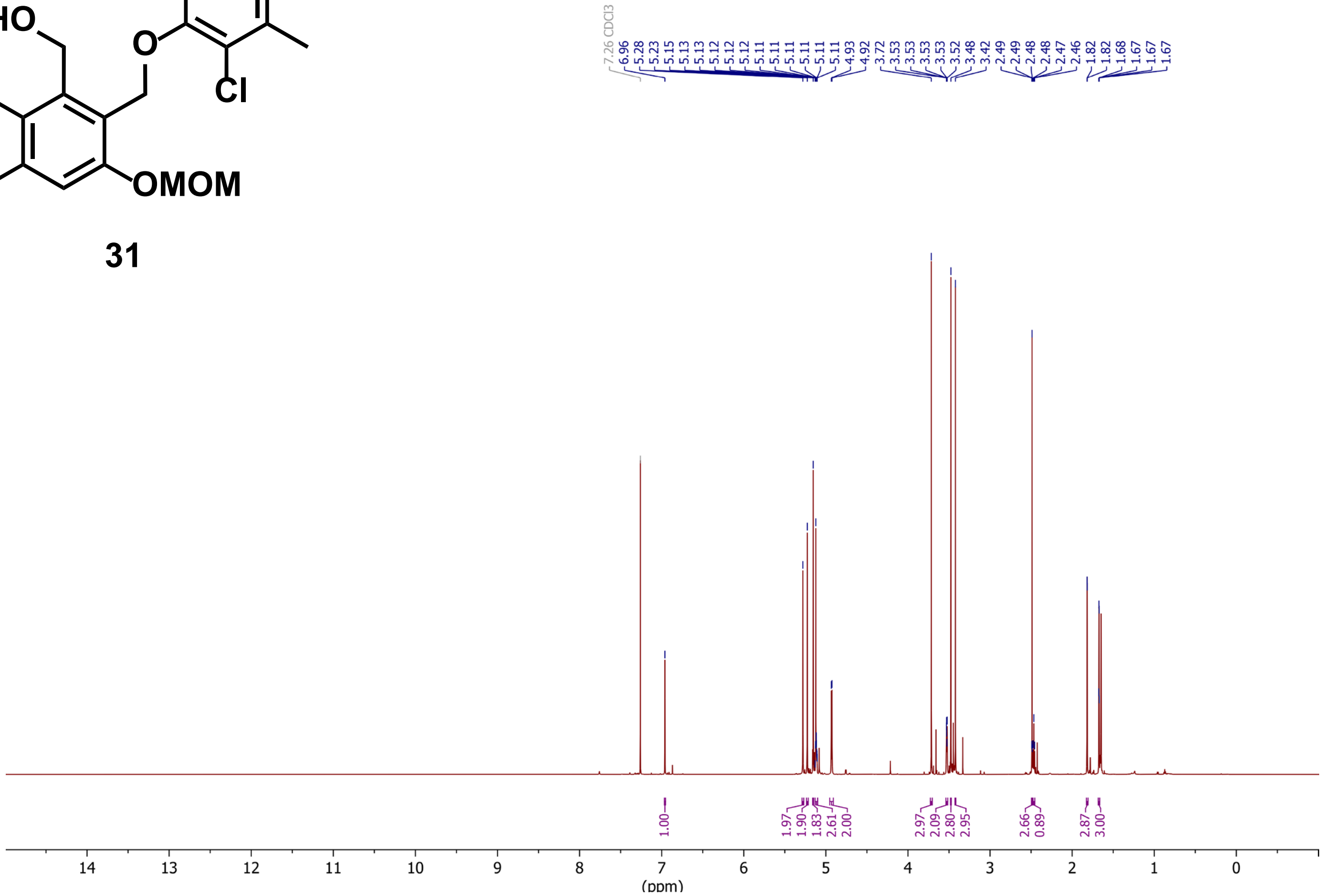
Frequency: 800.29 MHz — Nucleus: 1H — Solvent: CDCl3
7.26 CDCl3
6.96
5.28
5.23
5.15
5.13
5.13
5.12
5.12
5.12
5.11
5.11
5.11
5.11
5.11
5.11
4.93
4.92
3.72
3.53
3.53
3.53
3.53
3.52
3.48
3.42
2.49
2.49
2.48
2.48
2.47
2.46
1.82
1.82
1.68
1.67
1.67
1.67
1.00
1.97
1.90
1.83
2.61
2.00
2.97
2.09
2.80
2.95
2.66
0.89
2.87
3.00
14
13
12
11
10
9
8
7
6
5
4
3
2
1
0
(ppm)

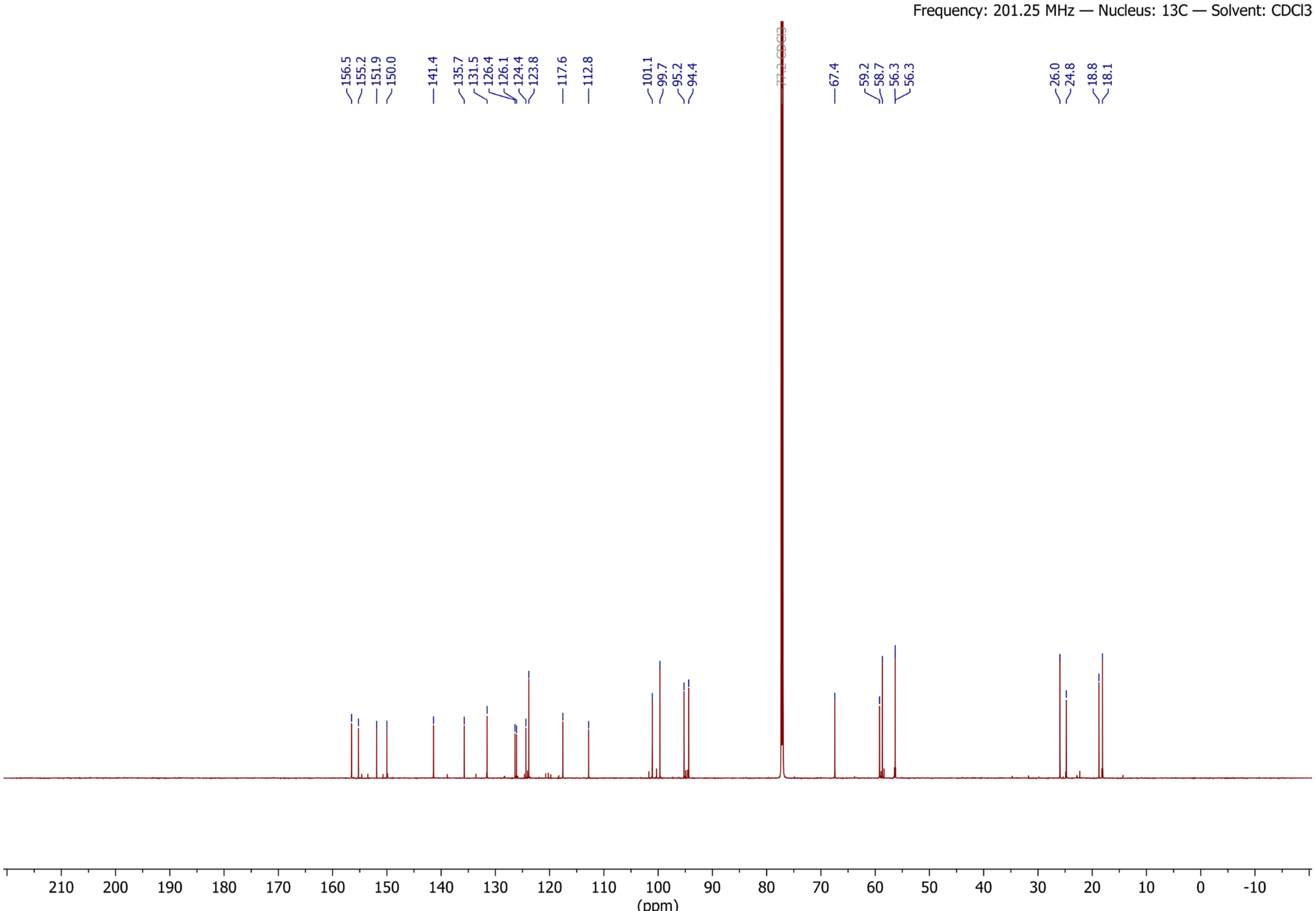
Frequency: 201.25 MHz — Nucleus: 13C — Solvent: CDCl3
156.5
155.2
151.9
150.0
141.4
135.7
131.5
126.4
126.1
124.4
123.8
117.6
112.8
101.1
99.7
95.2
94.4
77.2 CDCl3
67.4
59.2
58.7
56.3
56.3
26.0
24.8
18.8
18.1
210
200
190
180
170
160
150
140
130
120
110
100
90
80
70
60
50
40
30
20
10
0
-10
(ppm)

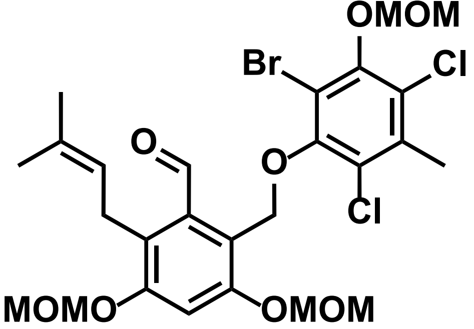

32

Frequency: 800.29 MHz — Nucleus: 1H — Solvent: CDCl3

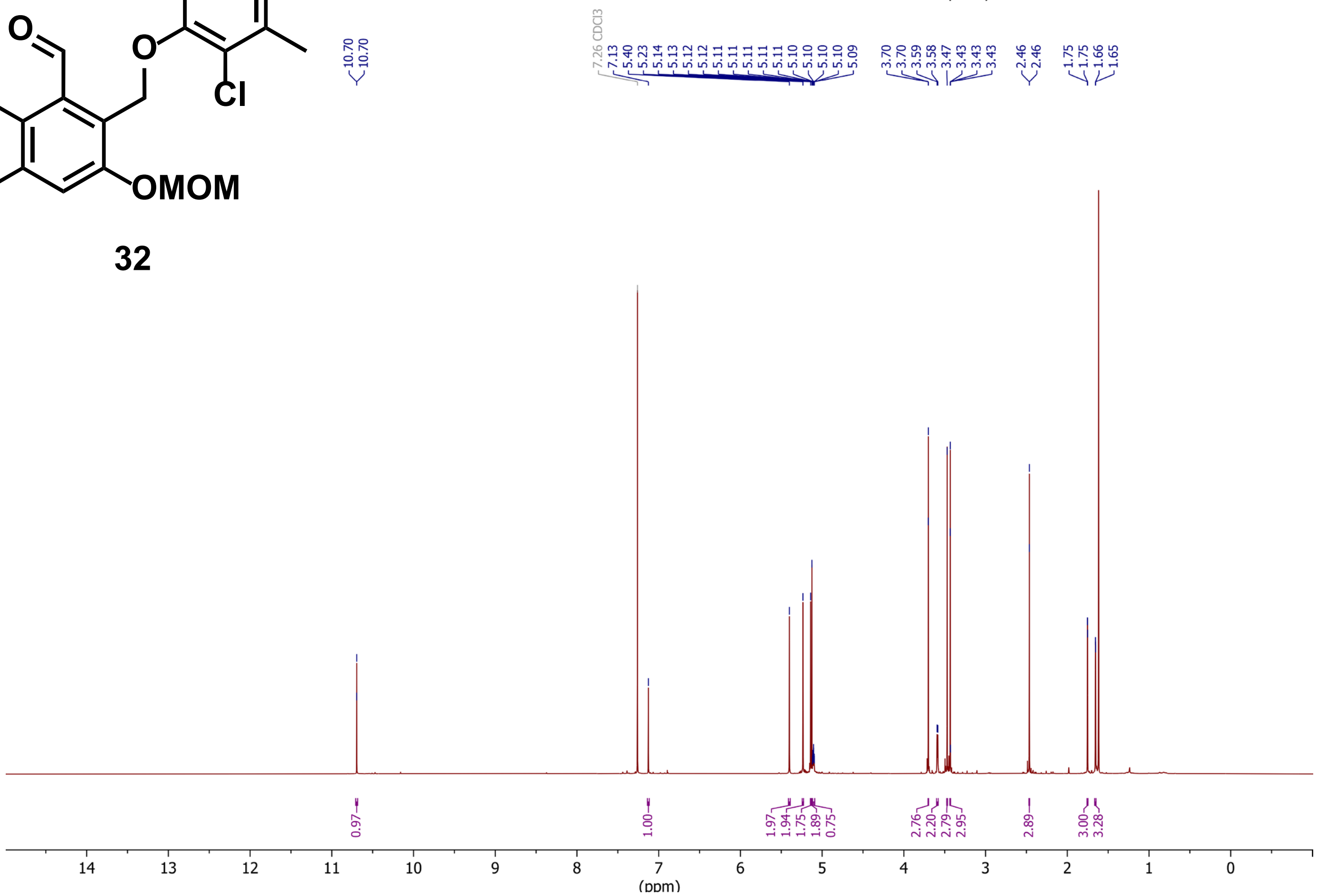

Frequency: 201.25 MHz — Nucleus: 13C — Solvent: CDCl3

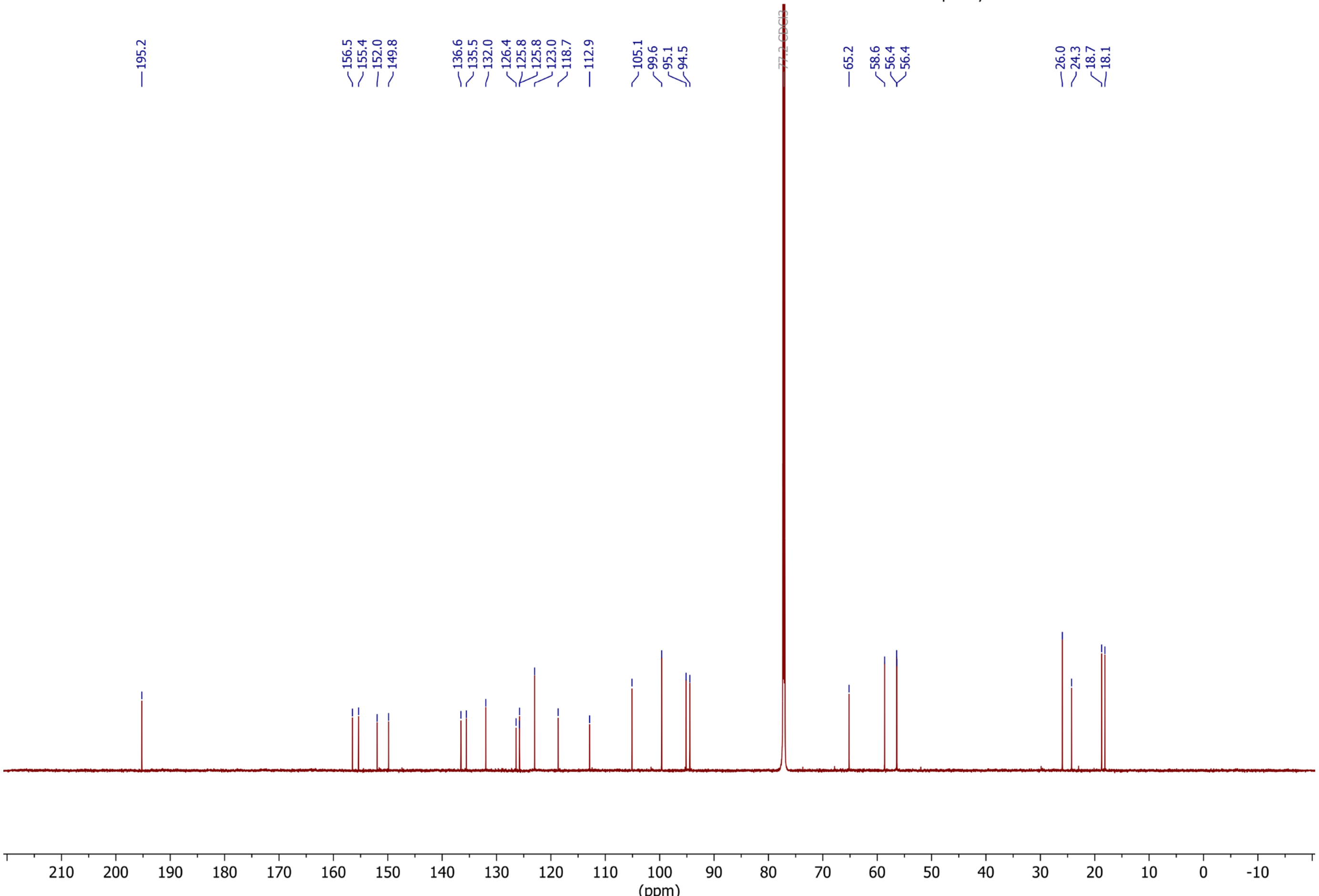

Experiment: HSQC — Frequency: 800.29 MHz — Nucleus: (1H, 13C) — Solvent: CDCl3

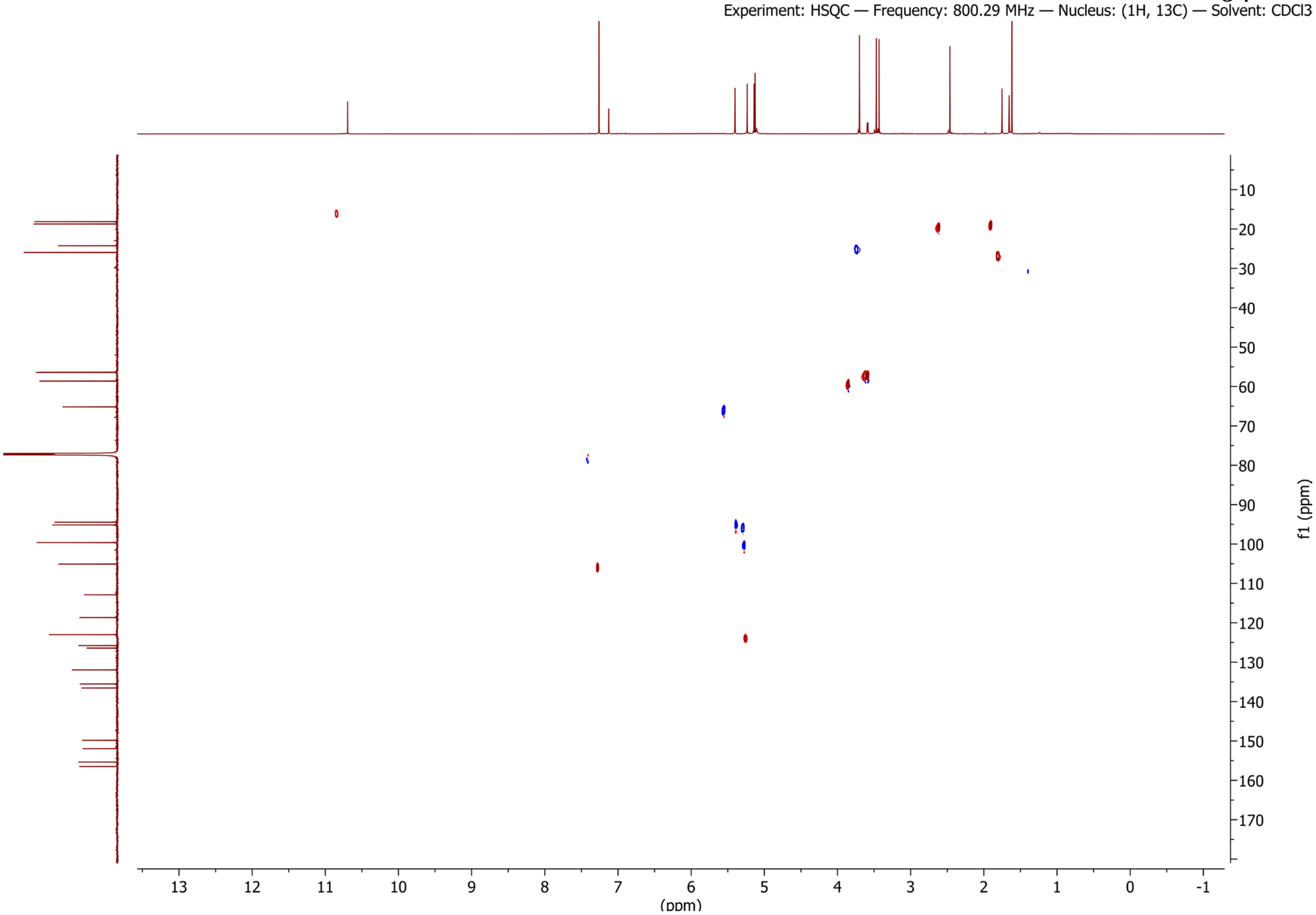

Experiment: HMBC Frequency: 800.29 MHz — Nucleus: (1H, 13C) — Solvent: CDCl3

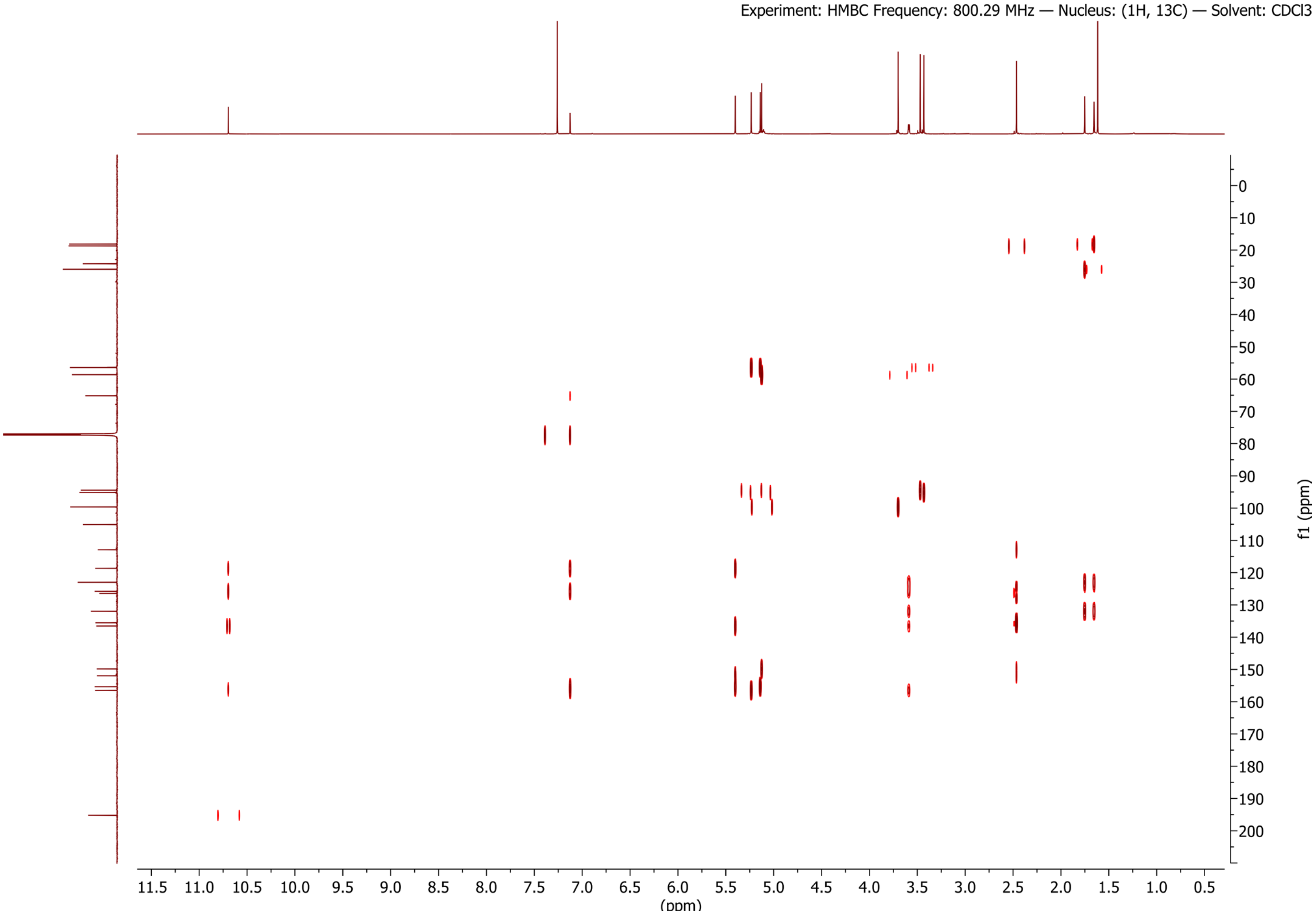

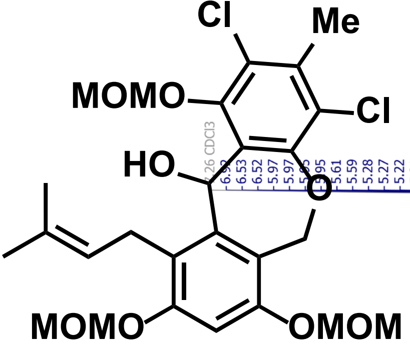
Cl
Me
MOMO
Cl
HO
O
MOMO
OMOM

33

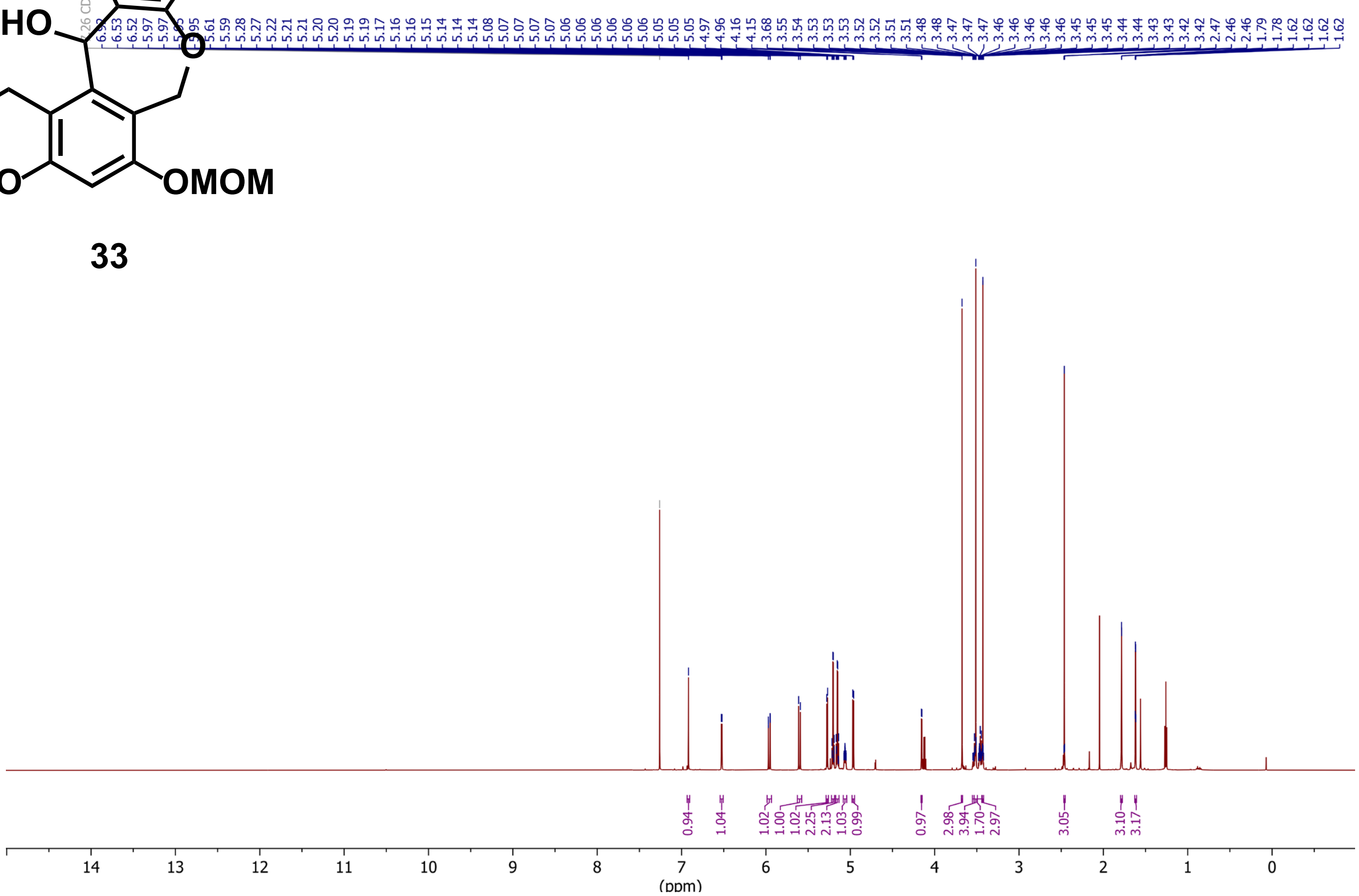
Frequency: 600.13 MHz — Nucleus: 1H — Solvent: CDCl3
(ppm)

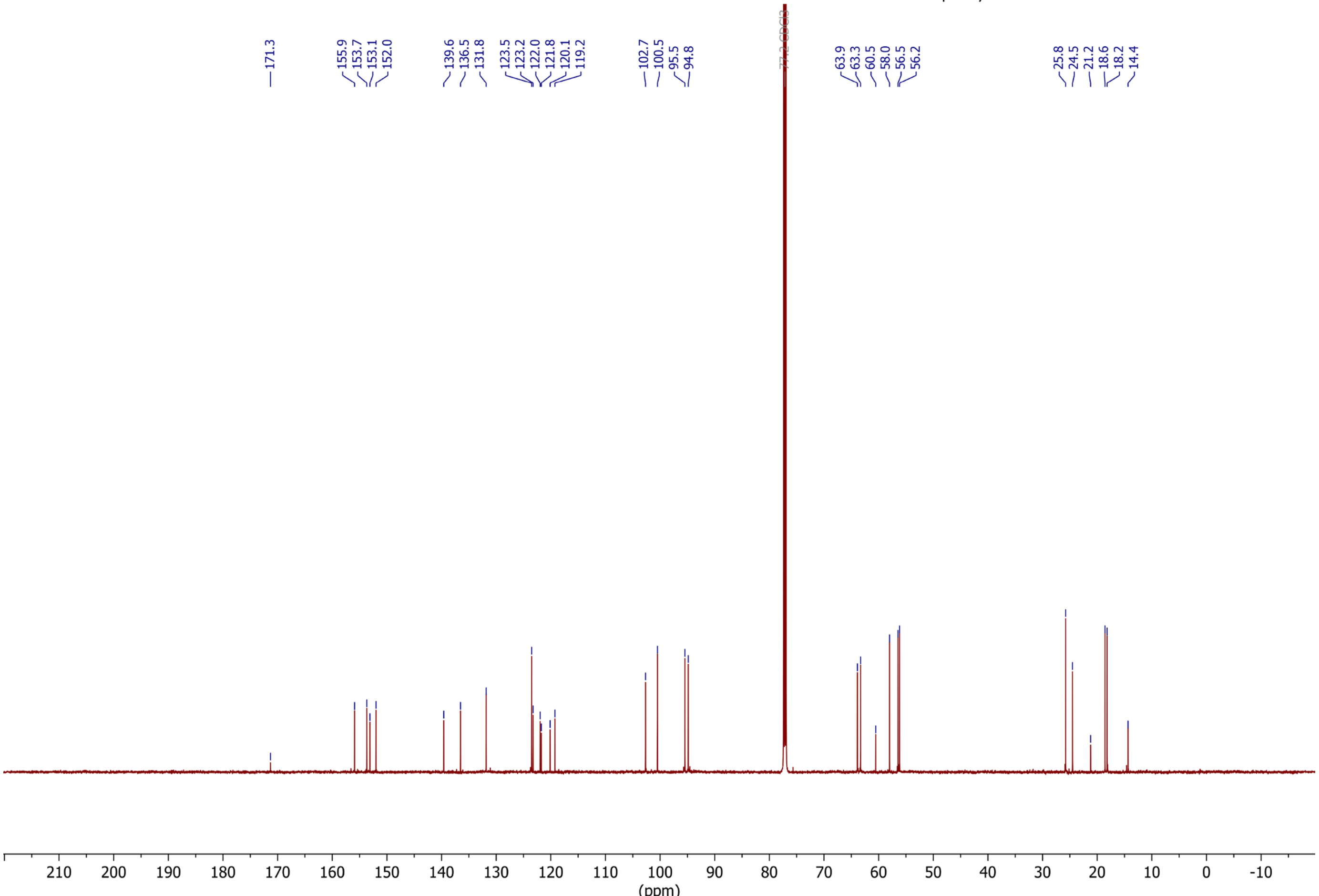
Frequency: 150.92 MHz — Nucleus: 13C — Solvent: CDCl3
171.3
155.9
153.7
153.1
152.0
139.6
136.5
131.8
123.5
123.2
122.0
121.8
120.1
119.2
102.7
100.5
95.5
94.8
63.9
63.3
60.5
58.0
56.5
56.2
25.8
24.5
21.2
18.6
18.2
14.4
(ppm)

Experiment: HSQC — Frequency: 600.13 MHz — Nucleus: (1H, 13C) — Solvent: CDCl3

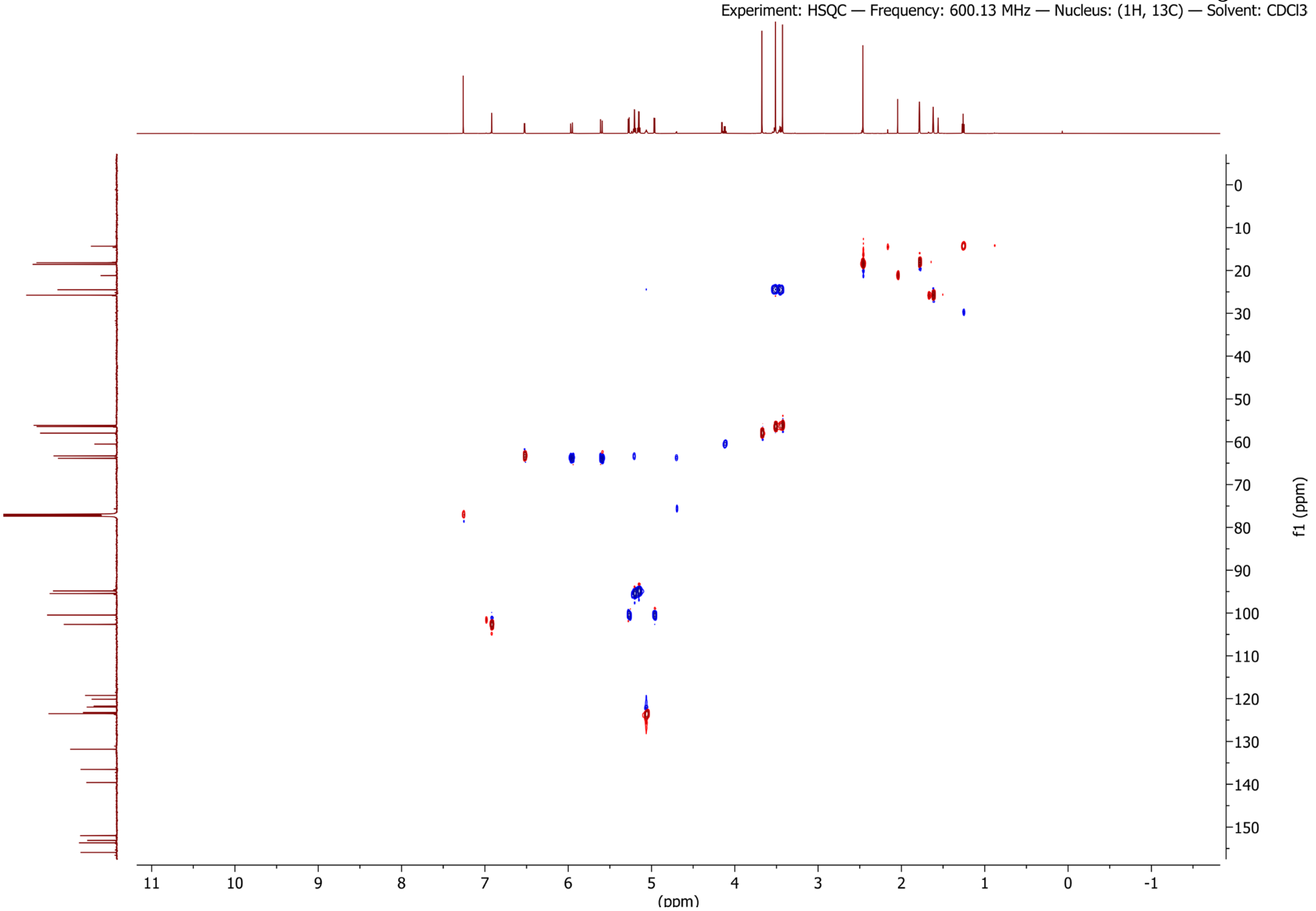

Experiment: HMBC — Frequency: 600.13 MHz — Nucleus: (1H, 13C) — Solvent: CDCl3

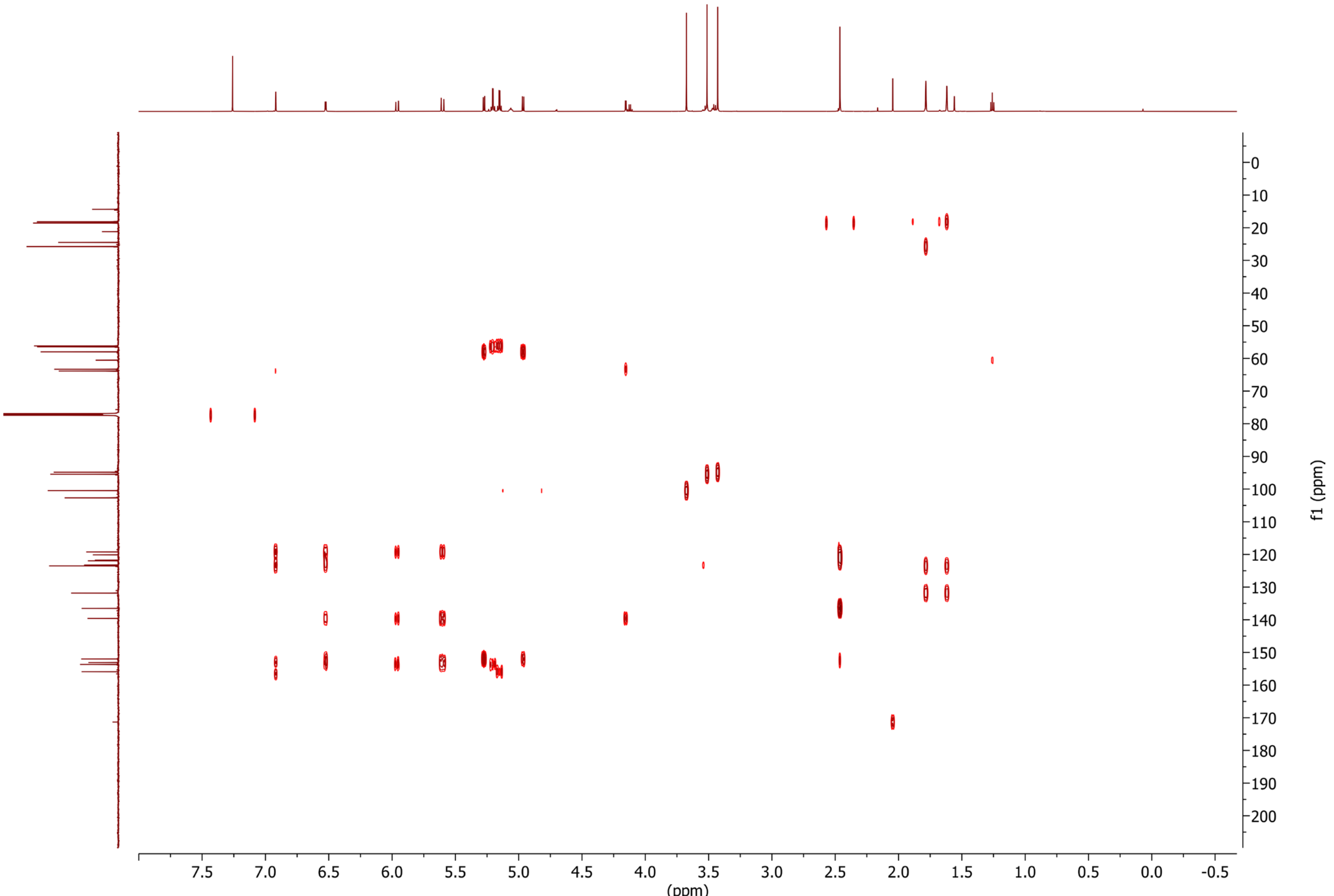

X-ray Crystallography Report for 22

(The following crystallography report prepared by the X-ray crystallography center at Emory University)

Submitted by: **Benjamin Deprez**

Solved by: **John Bacsa**

R_1=4.03%

Crystal Data and Experimental

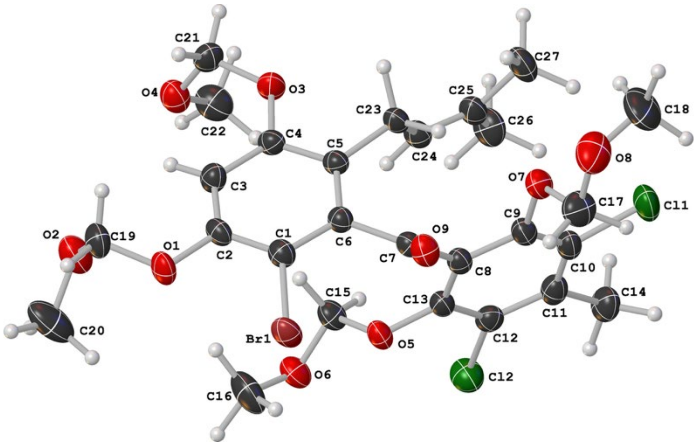

Experimental. Single colorless needle-shaped crystals of WY-4-55-P3 were used as supplied. A suitable crystal with dimensions 0.20 × 0.04 × 0.03 mm^3 was selected and mounted on a XtaLAB AFC11 (RCD3): quarter-chi single diffractometer. The crystal was kept at a constant T = 173.0(1) K during data collection. The structure was solved with ShelXT (Sheldrick, 2015) and by using Olex2 1.5-alpha (Dolomanov et al., 2009). The structure was refined with olex2.refine 1.5-alpha (Bourhis et al., 2015) using full matrix least squares minimisation on $\boldsymbol{F^2}$.

Crystal Data. $C_{27}H_{33}BrCl_2O_9$, M_r = 652.37, triclinic, P-1 (No. 2), a = 8.9711(7) Å, b = 12.5254(10) Å, c = 13.1847(9) Å, α = 79.147(7)°, β = 81.27(1)°, γ = 83.33(1)°, V = 1432.3(2) Å^3, T = 173.02(10) K, Z = 2, Z' = 1, μ(Cu K_α) = 4.118, 36155 reflections measured, 5417 unique (R_{int} = 0.0816) which were used in all calculations. The final wR_2 was 0.0811 (all data) and R_1 was 0.0403 (I≥2 σ(I)).

**Compound**	WY-4-55-P3
Formula	$C_{27}H_{33}BrCl_2O_9$
$D_{calc.}$/ g cm^{-3}	1.513
μ/mm^{-1}	4.118
Formula Weight	652.37
Color	colorless
Shape	needle-shaped
Size/mm^3	0.20×0.04×0.03
T/K	173.02(10)
Crystal System	triclinic
Space Group	P-1
a/Å	8.9711(7)
b/Å	12.5254(10)
c/Å	13.1847(9)
α/°	79.147(7)
β/°	81.27(1)
γ/°	83.330(7)
V/Å^3	1432.28(19)
Z	2
Z'	1
Wavelength/Å	1.54184
Radiation type	Cu K$_\alpha$
Θ_{min}/°	3.44
Θ_{max}/°	72.89
Measured Refl's.	36155
Indep't Refl's	5417
Refl's I≥2 σ(I)	3752
R_{int}	0.0816
Parameters	554
Restraints	393
Largest Peak	0.5175
Deepest Hole	-0.7636
GooF	0.9938
wR_2 (all data)	0.0811
wR_2	0.0701
R_1 (all data)	0.0731
R_1	0.0403

Structure Quality Indicators

**Reflections:**	d min (CuKα) 2Θ=145.8° 0.81	I/σ(I) 16.5	Rint m=6.66 8.16%	Full 135.4° 95% to 145.8° 98.6
**Refinement:**	Shift 0.001	Max Peak 0.5	Min Peak -0.8	GooF 0.994

A colourless needle-shaped-shaped crystal with dimensions 0.20 × 0.04 × 0.03 mm^3 was mounted. Data were collected using a XtaLAB AFC11 (RCD3): quarter-chi single diffractometer operating at T = 173.02(10) K.

Data were measured using ω scans with Cu K_α radiation. The diffraction pattern was indexed and the total number of runs and images was based on the strategy calculation from the program CrysAlisPro 1.171.42.100a (Rigaku OD, 2023). The maximum resolution that was achieved was Θ = 72.89° (0.81 Å).

The unit cell was refined using CrysAlisPro 1.171.42.100a (Rigaku OD, 2023) on 1906 reflections, 5% of the observed reflections.

Data reduction, scaling and absorption corrections were performed using CrysAlisPro 1.171.42.100a (Rigaku OD, 2023). The final completeness is 98.61 % out to 72.89° in Θ. A numerical absorption correction based on gaussian integration over a multifaceted crystal model was performed using CrysAlisPro 1.171.42.74a (Rigaku Oxford Diffraction, 2022). An empirical absorption correction using spherical harmonics, implemented in SCALE3 ABSPACK scaling algorithm was also applied. The absorption coefficient μ of this material is 4.118 mm^{-1} at this wavelength (λ = 1.54184Å) and the minimum and maximum transmissions are 0.515 and 1.000.

The structure was solved and the space group P-1 (# 2) determined by the ShelXT (Sheldrick, 2015) structure solution program using dual methods and refined by full matrix least squares minimisation on $\boldsymbol{F^2}$ using version of olex2.refine 1.5-alpha (Bourhis et al., 2015). Hydrogen atom positions were determined from the charge density and refined using the Hirshfeld model. SOFTWARE: ORCA 5.0 PARTITIONING: NoSpherA2 INT ACCURACY: Normal METHOD: PBE BASIS SET: def2-SVP CHARGE: 0 MULTIPLICITY: 1 DATE: 2025-08-15_13-52-57

There is a single formula unit in the asymmetric unit, which is represented by the reported formula. The number of formula units in the unit cell, Z is 2 and the number of formula units, Z' in the symmetry independent unit is 1. The moiety formula is C27 H33 Br Cl2 O9.

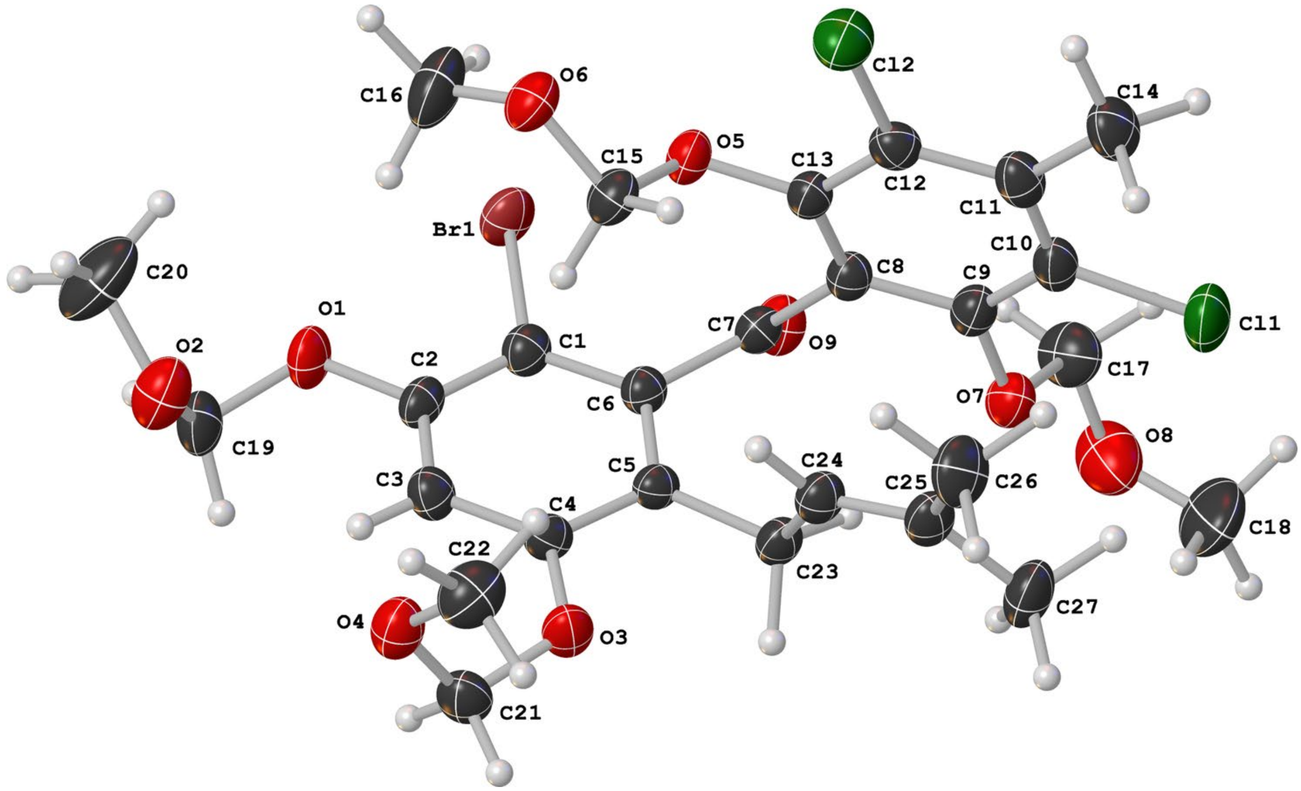

Figure 3 The molecular structure shown using thermal ellipsoids for the non-hydrogen atom (shown at the 50% probability level)

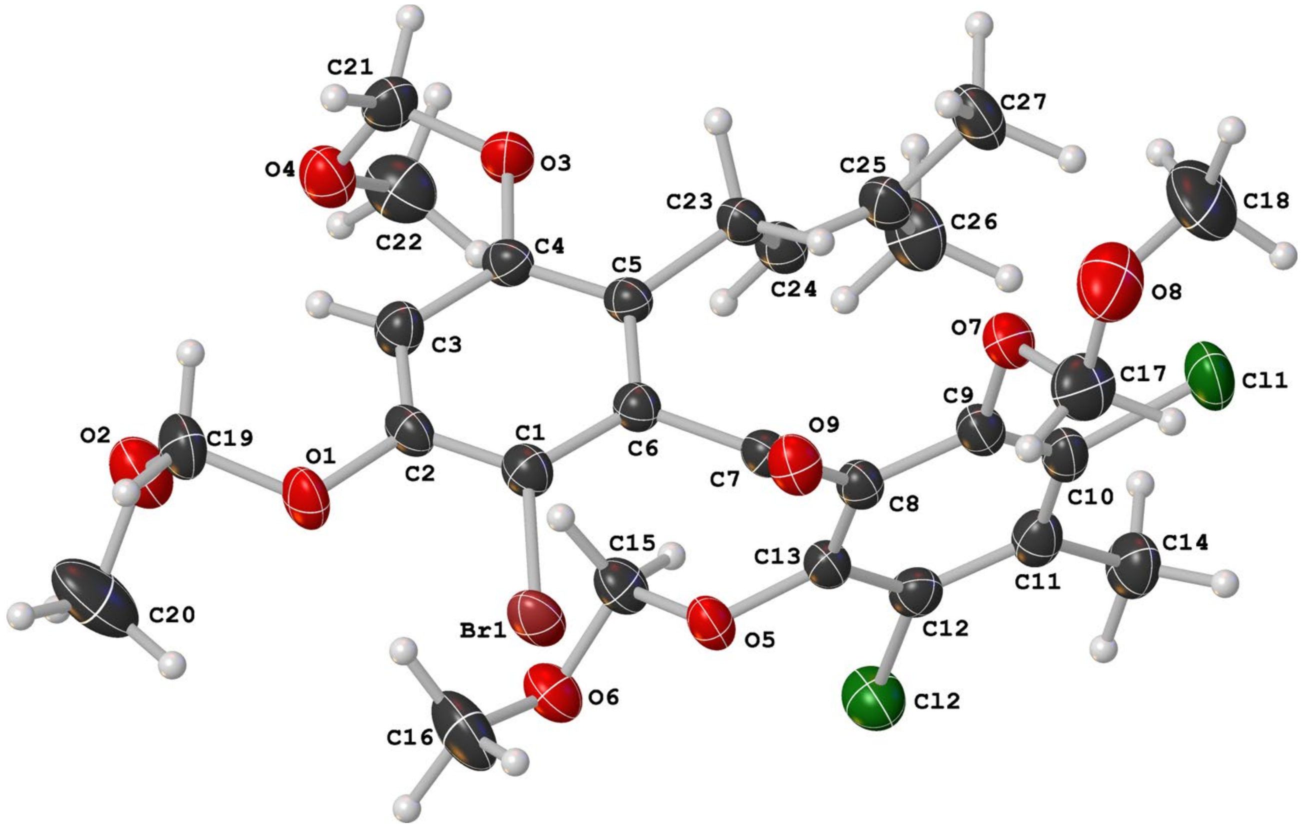

Figure 4 The molecular structure shown using thermal ellipsoids for the non-hydrogen atom (shown at the 50% probability level)

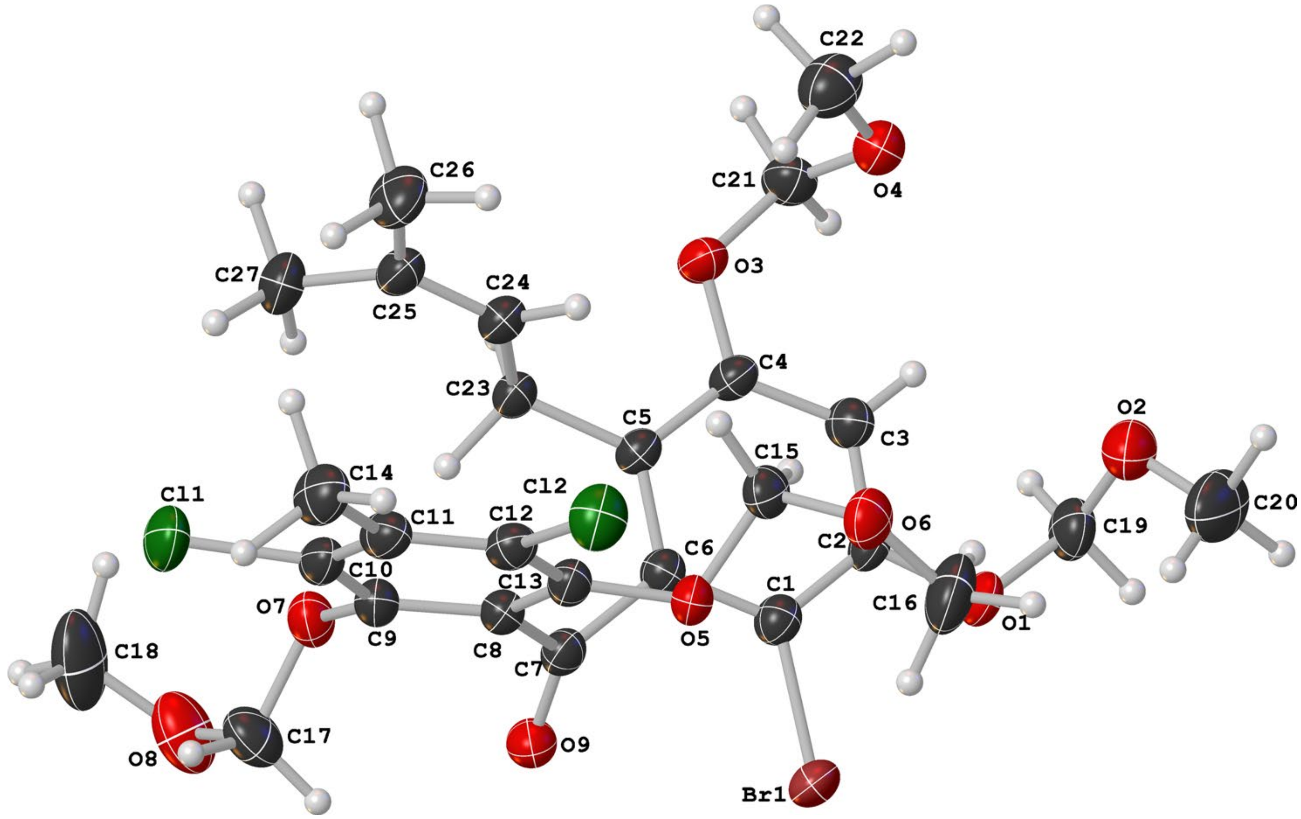

Figure 5 The molecular structure shown using thermal ellipsoids for the non-hydrogen atom (shown at the 50% probability level)

Data Plots: Diffraction Data

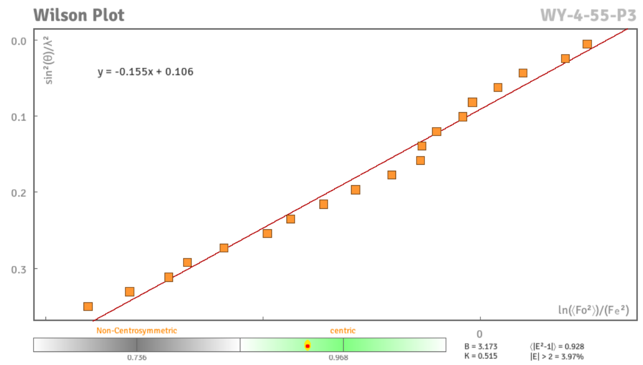

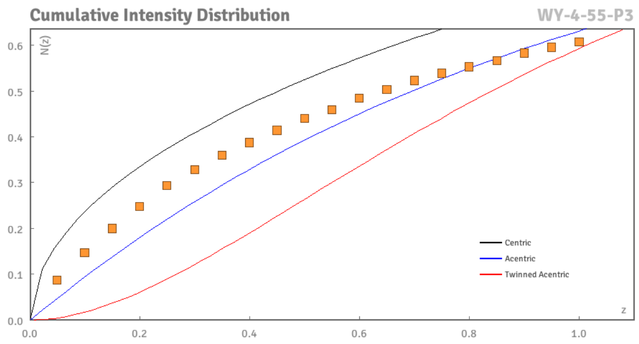

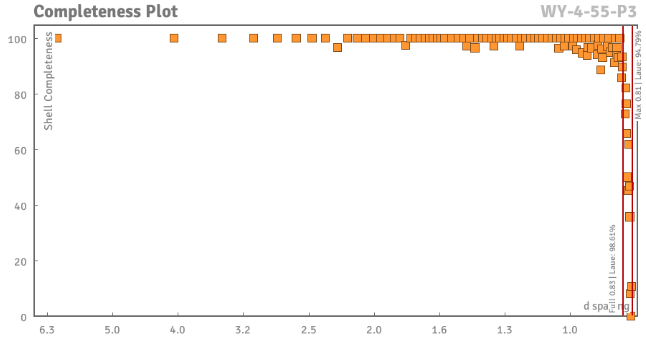

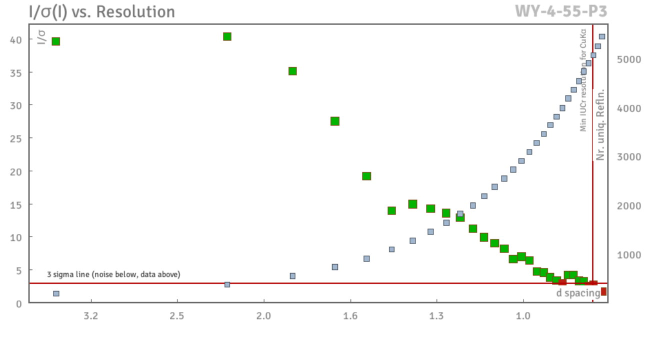

Data Plots: Refinement and Data

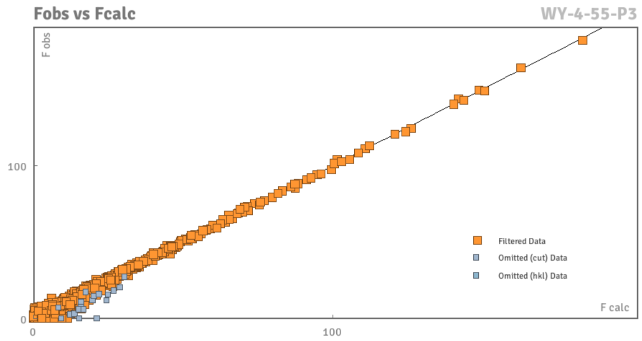

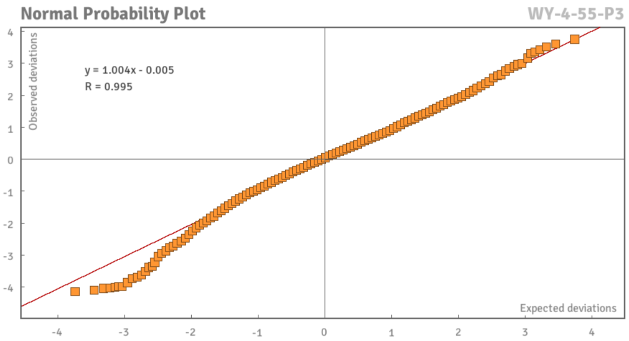

Reflection Statistics

Total reflections (after filtering)	36071	Unique reflections	5417
Completeness	0.948	Mean I/σ	11.58
hkl_{max} collected	(11, 15, 16)	hkl_{min} collected	(-10, -15, -16)
hkl_{max} used	(11, 15, 16)	hkl_{min} used	(-10, -14, 0)
Lim d_{max} collected	100.0	Lim d_{min} collected	0.77
d_{max} used	12.83	d_{min} used	0.81
Friedel pairs	3620	Friedel pairs merged	1
Inconsistent equivalents	4	R_{int}	0.0815
R_{sigma}	0.0605	Intensity transformed	0
Omitted reflections	0	Omitted by user (OMIT hkl)	84
Multiplicity	(1872, 1560, 1318, 1188, 851, 670, 539, 373, 240, 155, 96, 62, 56, 32, 29, 9, 4, 3, 2)	Maximum multiplicity	24
Removed systematic absences	0	Filtered off (Shel/OMIT)	0

Table 8: Fractional Atomic Coordinates (×10^4) and equivuivalent Isotropic Displacement Parameters (Å^2×10^3) for WY-4-55-P3. U_{eq} is defined as 1/3 of the trace of the orthogonalised U_{ij}.

Atom	x	y	z	U_{eq}
Br1	1636.4(8)	1569.1(6)	4420.2(6)	37.27(11)
Cl2	4903.9(10)	5991.9(7)	2128.5(7)	45.2(2)
Cl1	8108.0(10)	3444.3(8)	-481.8(7)	47.0(2)
O5	3883(2)	3885.6(17)	3303.1(16)	32.2(5)
O1	2577(2)	1225.7(18)	6510.3(17)	37.5(5)
O3	7977(2)	1354.9(18)	5464.5(16)	35.0(5)
O7	6687(2)	1664.4(17)	986.7(16)	35.9(5)
O9	4032(2)	1149.6(18)	2470.9(17)	35.9(5)
O4	7873(2)	2007.3(19)	7021.3(18)	43.2(6)
O6	3352(2)	4858.0(18)	4647.3(18)	41.7(6)
O2	3040(3)	1780(2)	8015(2)	53.6(7)
O8	6633(3)	328(2)	41(2)	64.0(8)
C8	5249(3)	2779(2)	2140(2)	27.8(7)
C9	6208(3)	2675(3)	1214(2)	31.7(7)
C2	3843(3)	1337(2)	5784(2)	29.6(7)
C5	6347(3)	1525(2)	4217(2)	26.6(7)
C13	4886(3)	3806(3)	2427(2)	29.0(7)
C6	4878(3)	1585(2)	3961(2)	27.3(7)
C1	3634(3)	1464(2)	4741(2)	31.5(7)
C11	6429(4)	4650(3)	850(2)	35.3(8)
C7	4651(3)	1775(3)	2831(2)	28.8(7)

Atom	x	y	z	U_{eq}
C4	6524(3)	1402(2)	5276(2)	28.9(7)
C10	6814(3)	3603(3)	601(2)	34.4(8)
C3	5286(3)	1337(2)	6057(3)	33.1(7)
C24	8380(3)	2674(3)	3216(2)	32.4(7)
C21	8304(4)	1126(3)	6502(3)	38.2(8)
C12	5448(3)	4731(3)	1779(2)	31.6(7)
C23	7750(3)	1571(2)	3424(2)	30.5(7)
C15	4456(4)	4196(3)	4161(3)	34.9(8)
C19	2770(4)	913(3)	7579(3)	42.1(8)
C25	9506(3)	2992(3)	2465(3)	37.3(8)
C26	10058(4)	4101(3)	2324(3)	51.5(10)
C17	5914(5)	1310(3)	235(3)	49.3(10)
C22	8634(4)	2943(3)	6544(3)	53.4(10)
C14	7055(4)	5628(3)	147(3)	46.7(10)
C27	10325(4)	2254(3)	1743(3)	49.6(10)
C16	2138(5)	4298(3)	5225(3)	64.7(13)
C20	1732(5)	2451(4)	8283(4)	77.0(14)
C18	8064(6)	413(4)	-553(4)	84.6(15)

Table 9: Anisotropic Displacement Parameters (×10[4]) for WY-4-55-P3. The anisotropic displacement factor exponent takes the form: $-2\pi^2[h^2a^{*2} \times U_{11} + \dots + 2hka^* \times b^* \times U_{12}]$

Atom	U_{11}	U_{22}	U_{33}	U_{23}	U_{13}	U_{12}
Br1	22.39(17)	50.2(2)	40.2(2)	-6.10(15)	0.07(15)	-11.95(17)
Cl2	50.3(5)	33.5(5)	48.9(6)	-4.9(4)	1.4(4)	-5.5(4)
Cl1	43.6(5)	60.1(6)	32.7(5)	-7.9(4)	8.9(4)	-5.8(4)
O5	27.7(11)	37.8(13)	31.0(12)	-3.7(9)	2.7(9)	-10.2(9)
O1	29.7(12)	48.0(14)	32.5(12)	-11.3(10)	7.5(9)	-5.8(10)
O3	24.2(11)	46.7(14)	33.6(12)	-2.5(10)	-4.0(9)	-5.9(10)
O7	36.3(13)	39.9(13)	30.6(13)	0.5(10)	-0.1(10)	-9.5(11)
O9	36.1(13)	41.5(14)	33.0(13)	-12.3(11)	-2.4(10)	-10.0(11)
O4	35.8(13)	56.2(14)	38.4(14)	-5.5(9)	-3.1(10)	-11.0(9)
O6	42.3(13)	40.9(13)	41.7(14)	1.2(9)	1.8(9)	-15.3(10)
O2	46.8(14)	67.1(17)	49.9(16)	-12.4(11)	4.1(11)	-21.7(11)
O8	93(2)	48.3(15)	50.8(17)	3.0(12)	-7.9(13)	-17.2(12)
C8	25.6(16)	33.3(18)	24.1(17)	-4.5(13)	1.8(13)	-7.4(14)
C9	29.4(17)	37.6(19)	26.9(18)	-4.1(14)	2.5(14)	-6.6(15)
C2	25.4(15)	30.7(17)	30.6(18)	-6.4(11)	3.6(11)	-3.4(14)
C5	19.7(15)	30.8(16)	27.6(17)	-3.2(11)	-0.7(11)	-2.4(13)
C13	29.4(17)	28.9(18)	27.1(17)	-2.0(14)	-1.3(14)	-3.4(14)
C6	24.2(16)	31.6(17)	24.7(17)	-3.8(13)	-0.6(13)	-2.7(14)
C1	32.0(18)	31.2(18)	29.9(18)	-7.1(14)	3.8(15)	-5.7(15)
C11	32.6(18)	41.7(19)	30.0(18)	-8.8(13)	-1.2(13)	-1.2(13)
C7	23.2(16)	33.1(18)	29.9(18)	-4.0(13)	-0.5(14)	-6.2(15)
C4	22.6(15)	33.5(17)	28.5(17)	-4.0(12)	-0.5(11)	-1.7(13)
C10	29.4(17)	44(2)	28.0(18)	-6.1(15)	-1.2(14)	-2.1(16)
C3	27.7(15)	38.9(19)	31.1(17)	-7.6(11)	1.4(10)	-3.4(13)
H3	28(8)	60(20)	31(3)	-11(5)	2.1(18)	-6(2)
C24	24.2(15)	38.3(17)	32.8(17)	-4.9(10)	2.9(11)	-5.6(11)
H24	55(16)	47(7)	60(14)	-22(5)	32(7)	-21(4)
C21	32.5(18)	46.9(19)	35.4(17)	-2.7(12)	-9.0(11)	-4.3(10)
H21a	38(7)	52(4)	39(6)	-7(2)	-9(3)	-3(2)
H21b	33(3)	54(8)	40(8)	-2.1(16)	-9.5(16)	-8(3)
C12	28.9(17)	32.8(18)	31.6(19)	-4.9(14)	-1.7(14)	-2.0(15)
C23	22.3(15)	38.3(17)	29.4(17)	-3.1(10)	0.6(11)	-5.5(11)
H23a	25(5)	41(5)	36(8)	-1(2)	-2(3)	-6(2)
H23b	26(8)	41(8)	30(4)	-5(3)	0(2)	-6(2)

Atom	U_{11}	U_{22}	U_{33}	U_{23}	U_{13}	U_{12}
C15	34.8(17)	36.2(18)	34.8(17)	-1.9(11)	-0.9(10)	-12.8(11)
H15a	45(8)	40(4)	41(6)	-2(2)	-5(3)	-10(2)
H15b	36(4)	39(7)	40(8)	-3(2)	-1(2)	-12(3)
C19	44(2)	49.2(19)	30.1(16)	-10.1(13)	6.7(12)	-3.7(11)
H19a	46(4)	59(7)	35(6)	-14(2)	7(2)	-5(3)
H19b	48(5)	57(5)	39(8)	-6(2)	2(2)	-9(2)
C25	25.2(15)	51.8(18)	32.5(17)	-7.8(11)	3.6(10)	-5.0(11)
C26	47(2)	56(2)	50(2)	-20.6(13)	9.0(17)	-7.4(14)
H26a	55(7)	62(7)	54(6)	-17(3)	10(3)	-11(3)
H26b	48(3)	70(9)	58(9)	-19.7(19)	7(2)	-13(4)
H26c	57(9)	61(7)	52(4)	-19(3)	7(2)	-6(2)
C17	60(2)	48(2)	46(2)	-3.9(14)	-12.0(16)	-18.0(14)
H17a	64(4)	54(10)	60(13)	-7(2)	-8(3)	-23(4)
H17b	68(11)	54(7)	51(6)	-5(4)	-13(4)	-14(3)
C22	49(2)	51(2)	61(3)	-8.1(13)	1.8(17)	-13.3(14)
H22a	56(8)	58(8)	62(4)	-8(3)	0(3)	-12(2)
H22b	55(9)	59(6)	68(7)	-7(3)	0(3)	-20(3)
H22c	49(3)	62(8)	69(9)	-7.0(17)	2(2)	-16(4)
C14	47(2)	48(2)	40(2)	-14.0(15)	2.8(16)	3.6(15)
H14a	49(3)	59(9)	47(8)	-15(2)	2(2)	1(4)
H14b	59(9)	51(8)	41(3)	-15(3)	0(2)	3(2)
H14c	56(7)	51(4)	50(8)	-11(2)	2(3)	0(2)
C27	37(2)	68(2)	43(2)	-5.3(15)	9.2(15)	-16.9(15)
H27a	38(3)	80(9)	53(9)	-5(2)	7(2)	-18(4)
H27b	44(8)	70(4)	56(9)	-8(2)	9(3)	-18(2)
H27c	48(9)	76(8)	45(3)	-8(3)	6(2)	-15(2)
C16	60(2)	68(3)	63(3)	-13.4(15)	22.1(16)	-24.2(18)
H16a	60(7)	73(8)	69(7)	-13(3)	20(3)	-28(3)
H16b	71(9)	72(6)	69(6)	-12(3)	18(3)	-21(3)
H16c	64(6)	78(7)	75(8)	-10(3)	21(3)	-33(3)
C20	56(2)	89(3)	95(4)	-0.9(16)	2.7(18)	-50(2)
H20a	71(9)	95(7)	101(6)	-2(3)	-5(3)	-46(3)
H20b	62(6)	98(8)	105(8)	-7(3)	5(3)	-49(3)
H20c	75(9)	103(7)	106(8)	-7(3)	-1(3)	-59(4)
C18	98(3)	83(3)	68(3)	12.2(18)	5.9(17)	-26(2)

Table 10: Bond Lengths in Å for WY-4-55-P3.

Atom	Atom	Length/Å
Br1	C1	1.887(3)
Cl2	C12	1.723(3)
Cl1	C10	1.725(3)
O5	C13	1.364(3)
O5	C15	1.443(4)
O1	C2	1.373(3)
O1	C19	1.420(4)
O3	C4	1.356(3)
O3	C21	1.412(4)
O7	C9	1.362(4)
O7	C17	1.451(4)
O9	C7	1.212(3)
O4	C21	1.393(4)
O4	C22	1.419(4)
O6	C15	1.378(3)
O6	C16	1.408(4)
O2	C19	1.381(4)
O2	C20	1.403(4)

Atom	Atom	Length/Å
O8	C17	1.370(4)
O8	C18	1.403(5)
C8	C9	1.401(4)
C8	C13	1.397(4)
C8	C7	1.508(4)
C9	C10	1.400(4)
C2	C1	1.392(4)
C2	C3	1.395(4)
C5	C6	1.400(4)
C5	C4	1.406(4)
C5	C23	1.509(4)
C13	C12	1.400(4)
C6	C1	1.400(4)
C6	C7	1.505(4)
C11	C10	1.403(4)
C11	C12	1.410(4)
C11	C14	1.499(4)
C4	C3	1.394(4)

Atom	Atom	Length/Å
C3	H3	1.1030
C24	H24	1.1030
C24	C23	1.514(4)
C24	C25	1.342(4)
C21	H21a	1.09(2)
C21	H21b	1.09(2)
C23	H23a	1.1110
C23	H23b	1.1110
C15	H15a	1.12(2)
C15	H15b	1.12(2)
C19	H19a	1.08(2)
C19	H19b	1.08(2)
C25	C26	1.498(5)
C25	C27	1.504(4)
C26	H26a	1.0970
C26	H26b	1.0970
C26	H26c	1.0970
C17	H17a	1.10(2)
C17	H17b	1.10(2)

Atom	Atom	Length/Å
C22	H22a	1.077(17)
C22	H22b	1.077(17)
C22	H22c	1.077(17)
C14	H14a	1.051(16)
C14	H14b	1.051(16)
C14	H14c	1.051(16)
C27	H27a	1.0970
C27	H27b	1.0970
C27	H27c	1.0970
C16	H16a	1.079(18)
C16	H16b	1.079(18)
C16	H16c	1.079(18)
C20	H20a	1.07(2)
C20	H20b	1.07(2)
C20	H20c	1.07(2)
C18	H18a	1.10(3)
C18	H18b	1.10(3)
C18	H18c	1.10(3)

Table 11: Bond Angles in ° for WY-4-55-P3.

Atom	Atom	Atom	Angle/°
C15	O5	C13	116.8(2)
C19	O1	C2	118.5(3)
C21	O3	C4	119.7(2)
C17	O7	C9	116.1(3)
C22	O4	C21	112.6(3)
C16	O6	C15	113.6(3)
C20	O2	C19	113.8(3)
C18	O8	C17	113.7(3)
C13	C8	C9	119.8(3)
C7	C8	C9	119.5(3)
C7	C8	C13	120.7(3)
C8	C9	O7	119.8(3)
C10	C9	O7	120.5(3)
C10	C9	C8	119.2(3)
C1	C2	O1	117.1(3)
C3	C2	O1	122.5(3)
C3	C2	C1	120.4(3)
C4	C5	C6	118.0(3)
C23	C5	C6	123.7(3)
C23	C5	C4	118.2(3)
C8	C13	O5	118.1(3)
C12	C13	O5	121.6(3)
C12	C13	C8	120.1(3)
C1	C6	C5	120.7(3)
C7	C6	C5	119.1(3)
C7	C6	C1	120.2(3)
C2	C1	Br1	118.2(2)
C6	C1	Br1	121.7(2)
C6	C1	C2	119.9(3)
C12	C11	C10	117.1(3)
C14	C11	C10	120.5(3)
C14	C11	C12	122.3(3)
C8	C7	O9	120.3(3)
C6	C7	O9	122.3(3)
C6	C7	C8	117.4(3)
C5	C4	O3	114.7(3)

Atom	Atom	Atom	Angle/°
C3	C4	O3	123.5(3)
C3	C4	C5	121.7(3)
C9	C10	Cl1	118.6(3)
C11	C10	Cl1	119.1(2)
C11	C10	C9	122.3(3)
C4	C3	C2	119.0(3)
H3	C3	C2	120.52(18)
H3	C3	C4	120.52(19)
C23	C24	H24	117.36(16)
C25	C24	H24	117.4(2)
C25	C24	C23	125.3(3)
O4	C21	O3	112.6(3)
H21a	C21	O3	109.08(17)
H21a	C21	O4	109.08(17)
H21b	C21	O3	109.08(16)
H21b	C21	O4	109.08(17)
H21b	C21	H21a	107.8
C13	C12	Cl2	118.8(2)
C11	C12	Cl2	119.8(2)
C11	C12	C13	121.3(3)
C24	C23	C5	112.3(2)
H23a	C23	C5	109.15(17)
H23a	C23	C24	109.15(17)
H23b	C23	C5	109.15(17)
H23b	C23	C24	109.15(17)
H23b	C23	H23a	107.9
O6	C15	O5	109.0(3)
H15a	C15	O5	109.89(16)
H15a	C15	O6	109.89(17)
H15b	C15	O5	109.89(15)
H15b	C15	O6	109.89(17)
H15b	C15	H15a	108.3
O2	C19	O1	112.5(3)
H19a	C19	O1	109.10(16)
H19a	C19	O2	109.10(17)
H19b	C19	O1	109.10(17)

Atom	Atom	Atom	Angle/˚
H19b	C19	O2	109.10(19)
H19b	C19	H19a	107.8
C26	C25	C24	121.4(3)
C27	C25	C24	122.6(3)
C27	C25	C26	116.0(3)
H26a	C26	C25	109.5
H26b	C26	C25	109.5
H26b	C26	H26a	109.5
H26c	C26	C25	109.5
H26c	C26	H26a	109.5
H26c	C26	H26b	109.5
O8	C17	O7	107.6(3)
H17a	C17	O7	110.20(18)
H17a	C17	O8	110.2(2)
H17b	C17	O7	110.20(18)
H17b	C17	O8	110.2(2)
H17b	C17	H17a	108.5
H22a	C22	O4	109.5
H22b	C22	O4	109.5
H22b	C22	H22a	109.5
H22c	C22	O4	109.5
H22c	C22	H22a	109.5
H22c	C22	H22b	109.5
H14a	C14	C11	109.5
H14b	C14	C11	109.5
H14b	C14	H14a	109.5
H14c	C14	C11	109.5

Atom	Atom	Atom	Angle/˚
H14c	C14	H14a	109.5
H14c	C14	H14b	109.5
H27a	C27	C25	109.5
H27b	C27	C25	109.5
H27b	C27	H27a	109.5
H27c	C27	C25	109.5
H27c	C27	H27a	109.5
H27c	C27	H27b	109.5
H16a	C16	O6	109.5
H16b	C16	O6	109.5
H16b	C16	H16a	109.5
H16c	C16	O6	109.5
H16c	C16	H16a	109.5
H16c	C16	H16b	109.5
H20a	C20	O2	109.5
H20b	C20	O2	109.5
H20b	C20	H20a	109.5
H20c	C20	O2	109.5
H20c	C20	H20a	109.5
H20c	C20	H20b	109.5
H18a	C18	O8	109.5
H18b	C18	O8	109.5
H18b	C18	H18a	109.5
H18c	C18	O8	109.5
H18c	C18	H18a	109.5
H18c	C18	H18b	109.5

Table 12: Torsion Angles in ˚ for WY-4-55-P3.

Atom	Atom	Atom	Atom	Angle/˚
Br1	C1	C2	O1	4.1(3)
Br1	C1	C2	C3	-175.2(2)
Br1	C1	C6	C5	178.8(2)
Br1	C1	C6	C7	-1.6(3)
Cl2	C12	C13	O5	2.3(3)
Cl2	C12	C13	C8	177.0(2)
Cl2	C12	C11	C10	-179.2(3)
Cl2	C12	C11	C14	1.1(3)
Cl1	C10	C9	O7	3.1(3)
Cl1	C10	C9	C8	175.0(2)
Cl1	C10	C11	C12	-175.8(3)
Cl1	C10	C11	C14	3.9(3)
O5	C13	C8	C9	176.4(3)
O5	C13	C8	C7	-5.2(3)
O5	C13	C12	C11	-177.0(3)
O5	C15	O6	C16	71.9(3)
O1	C2	C1	C6	179.8(3)
O1	C2	C3	C4	177.0(3)
O1	C19	O2	C20	79.2(4)
O3	C4	C5	C6	-179.7(2)
O3	C4	C5	C23	1.0(3)
O3	C4	C3	C2	-176.7(3)
O3	C21	O4	C22	-61.1(3)
O7	C9	C8	C13	173.3(3)
O7	C9	C8	C7	-5.2(3)
O7	C9	C10	C11	-175.6(3)
O7	C17	O8	C18	-72.0(4)
O9	C7	C8	C9	-51.9(3)
O9	C7	C8	C13	129.7(3)
O9	C7	C6	C5	122.6(3)
O9	C7	C6	C1	-57.1(3)
O4	C21	O3	C4	-73.8(3)
O6	C15	O5	C13	143.4(2)
O2	C19	O1	C2	80.5(3)
O8	C17	O7	C9	174.8(3)
C8	C9	O7	C17	101.6(3)
C8	C9	C10	C11	-3.7(4)
C8	C13	O5	C15	114.9(3)
C8	C13	C12	C11	-2.4(4)
C8	C7	C6	C5	-56.5(3)
C8	C7	C6	C1	123.8(3)
C9	C8	C13	C12	1.6(4)
C9	C8	C7	C6	127.3(3)
C9	C10	C11	C12	2.9(4)
C9	C10	C11	C14	-177.4(3)
C2	C1	C6	C5	3.2(3)
C2	C1	C6	C7	-177.1(3)
C2	C3	C4	C5	3.4(4)
C5	C4	O3	C21	-173.9(3)
C5	C23	C24	C25	-171.7(3)
C13	C8	C9	C10	1.4(4)
C13	C8	C7	C6	-51.2(3)
C13	C12	C11	C10	0.2(4)
C13	C12	C11	C14	-179.5(3)
C6	C5	C4	C3	0.2(3)
C6	C5	C23	C24	103.3(3)

Atom	Atom	Atom	Atom	Angle/˚
C6	C1	C2	C3	0.5(4)
C1	C2	O1	C19	169.6(3)
C1	C2	C3	C4	-3.7(3)
C1	C6	C5	C4	-3.5(3)
C1	C6	C5	C23	175.7(3)
C7	C8	C9	C10	-177.1(3)
C7	C8	C13	C12	-180.0(3)
C7	C6	C5	C4	176.8(3)
C7	C6	C5	C23	-3.9(3)
C4	C5	C23	C24	-77.5(3)
C10	C9	O7	C17	-86.6(3)
C3	C2	O1	C19	-11.1(4)
C3	C4	O3	C21	6.2(4)
C3	C4	C5	C23	-179.1(3)
C12	C13	O5	C15	-70.4(3)
C23	C24	C25	C26	-179.6(3)
C23	C24	C25	C27	-1.9(4)

Table 13: Hydrogen Fractional Atomic Coordinates (×10[4]) and equivuivalent Isotropic Displacement Parameters (Å[2]×10[3]) for WY-4-55-P3. U_{eq} is defined as 1/3 of the trace of the orthogonalised U_{ij}.

Atom	x	y	z	U_{eq}
H3	5445(3)	1286(2)	6879(3)	41(8)
H24	7872(3)	3263(3)	3724(2)	55(10)
H21a	7720(11)	429(13)	6921(8)	43(4)
H21b	9510(20)	909(5)	6499(3)	42(4)
H23a	8629(3)	931(2)	3708(2)	35(4)
H23b	7475(3)	1392(2)	2683(2)	33(4)
H15a	4776(7)	3446(14)	4730(10)	42(4)
H15b	5495(19)	4644(8)	3865(6)	38(4)
H19a	1759(19)	563(8)	8006(9)	48(4)
H19b	3711(18)	289(13)	7653(3)	48(4)
H26a	9361(15)	4571(7)	2876(11)	58(4)
H26b	11242(8)	4017(3)	2469(16)	59(4)
H26c	9980(20)	4527(7)	1525(6)	58(4)
H17a	4720(30)	1219(3)	550(7)	57(6)
H17b	5960(4)	1916(13)	-490(15)	57(6)
H22a	8235(7)	3277(6)	5809(12)	59(4)
H22b	8412(5)	3546(10)	7046(8)	60(4)
H22c	9830(20)	2716(4)	6415(4)	60(4)
H14a	8200(18)	5639(3)	231(3)	53(4)
H14b	6965(4)	5589(3)	-630(13)	52(4)
H14c	6440(10)	6342(12)	348(4)	54(4)
H27a	11522(6)	2108(14)	1859(12)	58(4)
H27b	9815(16)	1474(7)	1910(11)	57(4)
H27c	10240(20)	2642(8)	933(3)	57(4)
H16a	1570(11)	3963(7)	4707(9)	68(5)
H16b	2564(8)	3645(12)	5796(10)	72(4)
H16c	1349(14)	4857(10)	5616(7)	73(4)
H20a	1362(9)	2935(10)	7591(13)	86(5)
H20b	856(17)	1955(10)	8686(8)	86(5)
H20c	1977(6)	2977(11)	8778(10)	90(5)
H18a	7969(6)	1020(15)	-1263(18)	142(12)
H18b	8840(20)	668(7)	-95(11)	142(12)
H18c	8502(13)	-380(20)	-764(7)	142(12)

Citations

CrysAlisPro Software System, Rigaku Oxford Diffraction, (2023).

L.J. Bourhis and O.V. Dolomanov and R.J. Gildea and J.A.K. Howard and H. Puschmann, The Anatomy of a Comprehensive Constrained, Restrained, Refinement Program for the Modern Computing Environment - Olex2 Disected, *Acta Cryst. A*, (2015), **A71**, 59-71.

O.V. Dolomanov and L.J. Bourhis and R.J. Gildea and J.A.K. Howard and H. Puschmann, Olex2: A complete structure solution, refinement and analysis program, *J. Appl. Cryst.*, (2009), **42**, 339-341.

Sheldrick, G.M., ShelXT-Integrated space-group and crystal-structure determination, *Acta Cryst.*, (2015), **A71**, 3-8.

X-ray Crystallography Report for 23

(The following crystallography report prepared by the X-ray crystallography center at Emory University)

Submitted by: **Benjamin Deprez**

Solved by: **John Bacsa**

R_1=3.93%

Crystal Data and Experimental

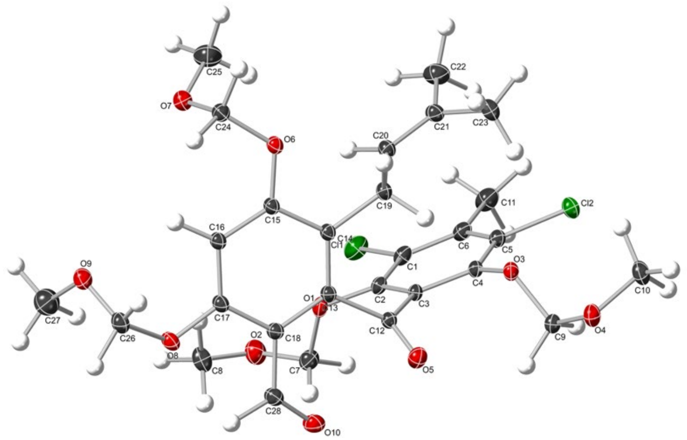

Experimental. Single colorless plate-shaped crystals of BD-6-195 were crystallised from ethyl acetate by slow evaporation. A suitable crystal with dimensions 0.41 × 0.30 × 0.27 mm^3 was selected and mounted on a loop with paratone on a XtaLAB Synergy, Dualflex, HyPix diffractometer. The crystal was kept at a steady T = 100.0(1) K during data collection. The structure was solved with the ShelXT 2018/2 (Sheldrick, 2018) solution program using dual methods and by using Olex2 1.5-alpha (Dolomanov et al., 2009) as the graphical interface. The model was refined with olex2.refine 1.5-alpha (Bourhis et al., 2015) using full matrix least squares minimisation on $\boldsymbol{F}^2$.

Crystal Data. $C_{28}H_{34}Cl_2O_{10}$, M_r = 601.481, triclinic, P-1 (No. 2), a = 9.8137(2) Å, b = 12.3147(3) Å, c = 14.0126(3) Å, α = 66.673(2)°, β = 82.916(2)°, γ = 67.184(2)°, V = 1432.45(6) Å^3, T = 100.00(10) K, Z = 2, Z' = 1, μ(Mo K$_\alpha$) = 0.283, 66054 reflections measured, 14639 unique (R_{int} = 0.0485) which were used in all calculations. The final wR_2 was 0.0882 (all data) and R_1 was 0.0393 (I≥2 σ(I)).

**Compound**	BD-6-195
Formula	$C_{28}H_{34}Cl_2O_{10}$
$D_{calc.}$/ g cm^{-3}	1.395
μ/mm^{-1}	0.283
Formula Weight	601.481
Colour	colorless
Shape	plate-shaped
Size/mm^3	0.41×0.30×0.27
T/K	100.00(10)
Crystal System	triclinic
Space Group	P-1
a/Å	9.8137(2)
b/Å	12.3147(3)
c/Å	14.0126(3)
α/°	66.673(2)
β/°	82.916(2)
γ/°	67.184(2)
V/Å^3	1432.45(6)
Z	2
Z'	1
Wavelength/Å	0.71073
Radiation type	Mo K$_\alpha$
Θ_{min}/°	2.36
Θ_{max}/°	37.87
Measured Refl's.	66054
Indep't Refl's	14639
Refl's I≥2 σ(I)	11007
R_{int}	0.0485
Parameters	611
Restraints	689
Largest Peak	0.9438
Deepest Hole	-0.4940
GooF	1.0102
wR_2 (all data)	0.0882
wR_2	0.0819
R_1 (all data)	0.0588
R_1	0.0393

Structure Quality Indicators

**Reflections:**	d min (MoKα) 2Θ=75.7° 0.58	I/σ(I) 23.0	Rint m=4.47 4.85%	Full 50.5° 95% to 75.7° 98.1
**Refinement:**	Shift -0.001	Max Peak 0.9	Min Peak -0.5	GooF 1.010

A colourless plate-shaped-shaped crystal with dimensions 0.41 × 0.30 × 0.27 mm^3 was mounted on a loop with paratone. Data were collected using a XtaLAB Synergy, Dualflex, HyPix diffractometer operating at T = 100.00(10) K.

Data were measured using ω scans with Mo K_α radiation. The diffraction pattern was indexed and the total number of runs and images was based on the strategy calculation from the program CrysAlisPro system (CCD 43.128a 64-bit (release 20-06-2024)). The maximum resolution that was achieved was Θ = 37.87° (0.58 Å).

Data reduction, scaling and absorption corrections were performed using CrysAlisPro 1.171.43.121a (Rigaku OD, 2024). The final completeness is 98.13 % out to 37.87° in Θ. The unit cell was refined using CrysAlisPro 1.171.43.121a (Rigaku OD, 2024) on 23306 reflections, 35% of the observed reflections.

A numerical absorption correction based on gaussian integration over a multifaceted crystal model was performed using CrysAlisPro 1.171.42.74a (Rigaku Oxford Diffraction, 2022). An empirical absorption correction using spherical harmonics, implemented in SCALE3 ABSPACK scaling algorithm was also applied. The absorption coefficient μ of this material is 0.283 mm^{-1} at this wavelength (λ = 0.71073Å) and the minimum and maximum transmissions are 0.394 and 1.000.

The structure was solved and the space group *P*-1 (# 2) determined by the ShelXT 2018/2 (Sheldrick, 2018) structure solution program using using dual methods and refined by full matrix least squares minimisation on $\boldsymbol{F^2}$ using version of olex2.refine 1.5-alpha (Bourhis et al., 2015). All non-hydrogen atoms were refined anisotropically. Refinement was by using NoSpherA2, an implementation of non-spherical atom-form-factors (F. Kleemiss, H. Puschmann, O. Dolomanov, S.Grabowsky - https://doi.org/10.1039/D0SC05526C – 2020). NoSpherA2 implementation of HAR makes use of tailor-made aspherical atomic form factors calculated from a Hirshfeld-partitioned electron density (ED) not from spherical-atom form factors. The ED was calculated from a Gaussian basis set single determinant SCF wavefunction from DFT using selected functionals for a fragment of this crystal. This fragment was embedded in an electrostatic crystal field by employing cluster charges. The following options were used: SOFTWARE: ORCA PARTITIONING: NoSpherA2 INT ACCURACY: Normal METHOD: PBE BASIS SET: def2-SVP CHARGE: 0 MULTIPLICITY: 1 DATE: 2024-08-28_13-37-27

There is a single formula unit in the asymmetric unit, which is represented by the reported sum formula. In other words: Z is 2 and Z' is 1. The moiety formula is C28 H34 Cl2 O10.

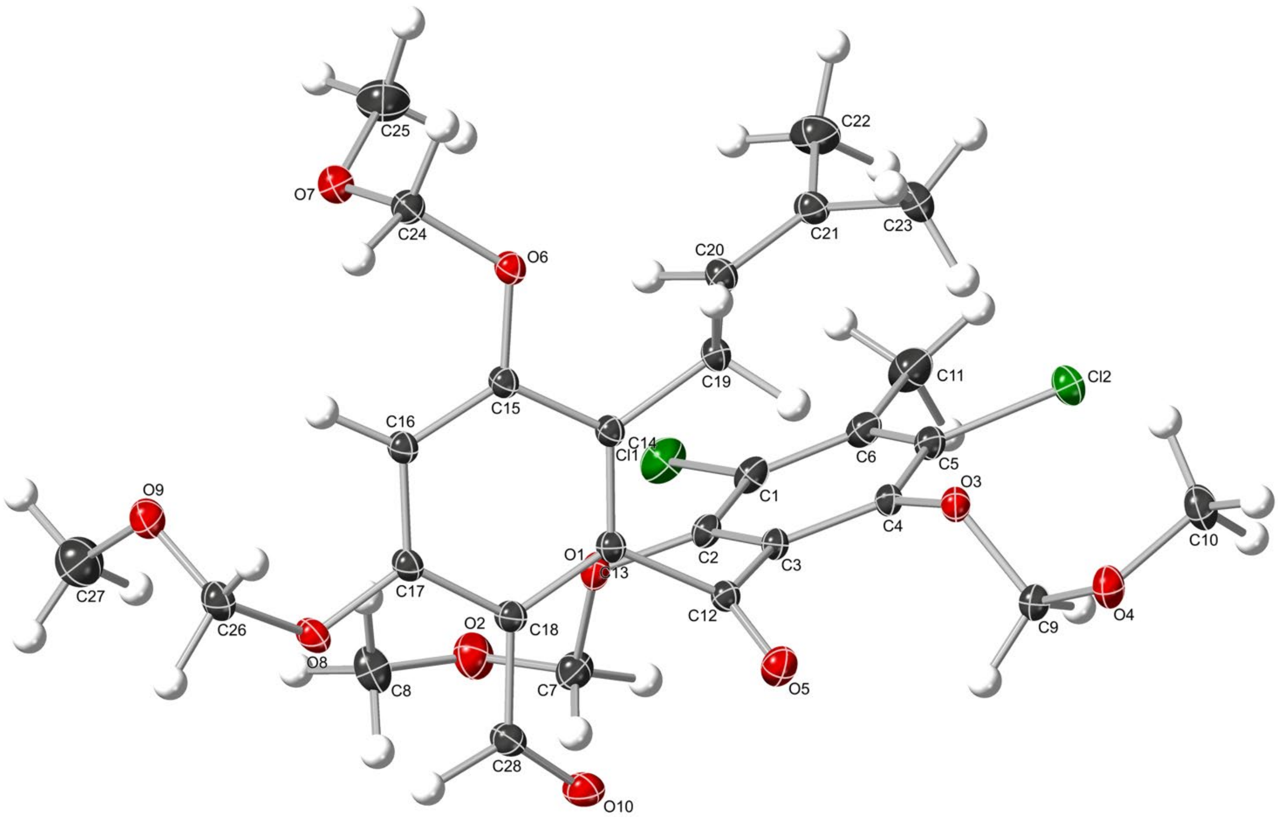

Figure 6 Thermal ellipsoidal representation (50% probability for all atoms, excluding hydrogens) of the molecular structure in the crystal structure.

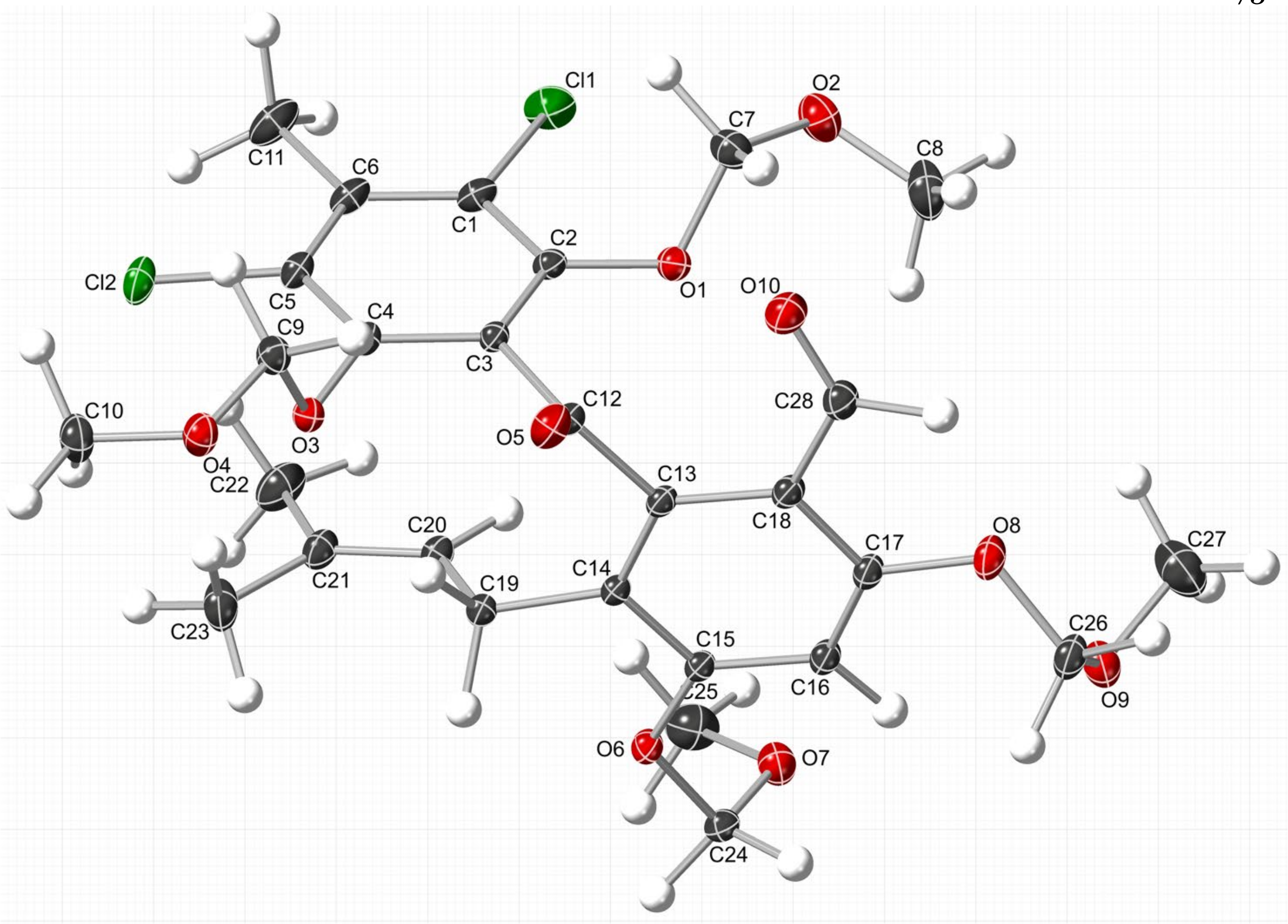

Figure 7 Thermal ellipsoidal representation (50% probability for all atoms, excluding hydrogens) of the molecular structure in the crystal structure.

Data Plots: Diffraction Data

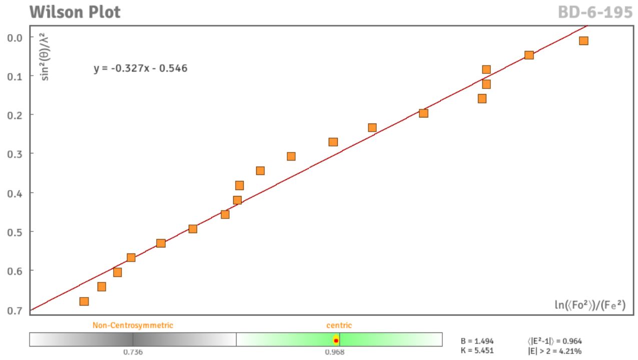

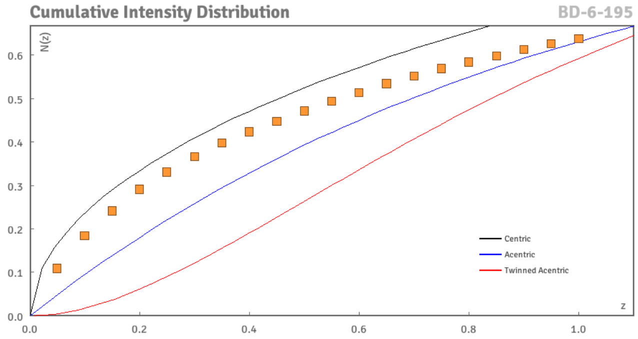

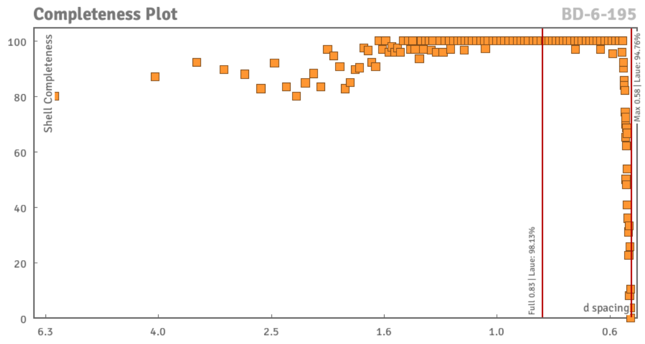

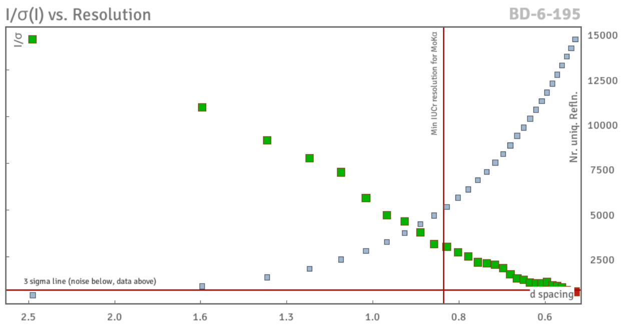

Data Plots: Refinement and Data

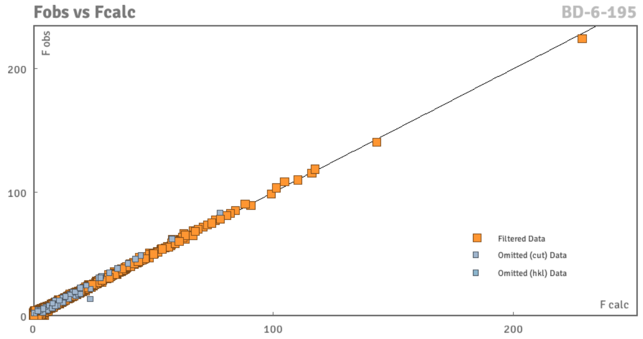

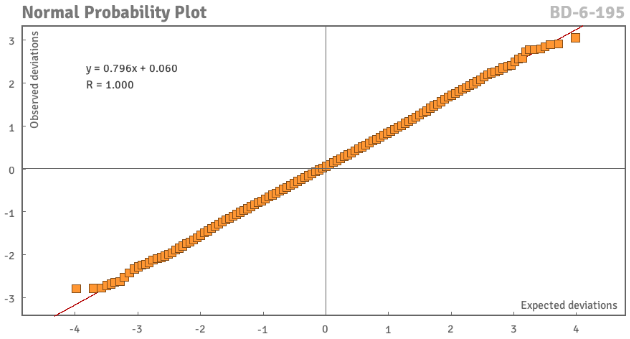

Reflection Statistics

Total reflections (after filtering)	65434	Unique reflections	14639
Completeness	0.948	Mean I/σ	13.73
hkl$_{max}$ collected	(16, 21, 24)	hkl$_{min}$ collected	(-16, -20, -23)
hkl$_{max}$ used	(16, 21, 24)	hkl$_{min}$ used	(-16, -18, 0)
Lim d$_{max}$ collected	100.0	Lim d$_{min}$ collected	0.36
d$_{max}$ used	8.62	d$_{min}$ used	0.58
Friedel pairs	11799	Friedel pairs merged	1
Inconsistent equivalents	0	R$_{int}$	0.0488
R$_{sigma}$	0.0434	Intensity transformed	0
Omitted reflections	620	Omitted by user (OMIT hkl)	0
Multiplicity	(7446, 8194, 5260, 3147, 1488, 655, 277, 51, 15)	Maximum multiplicity	16
Removed systematic absences	0	Filtered off (Shel/OMIT)	0

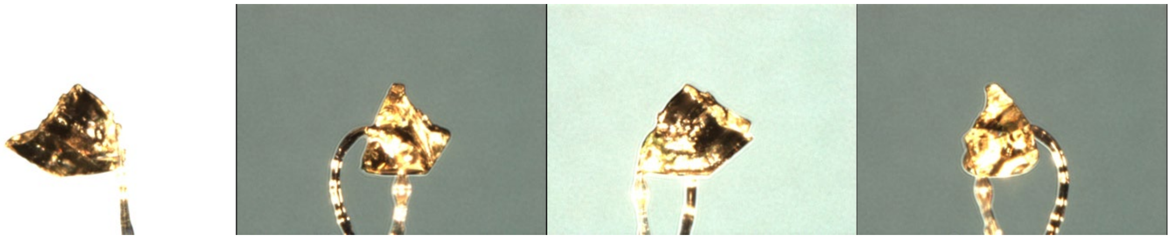

Table 14: Fractional Atomic Coordinates (×10^4) and equivuivalent Isotropic Displacement Parameters (Å^2×10^3) for BD-6-195. U_{eq} is defined as 1/3 of the trace of the orthogonalised U_{ij}.

Atom	x	y	z	U_{eq}
Cl1	1343.8(2)	6086.6(2)	9784.91(16)	25.50(5)
Cl2	5019.8(2)	1312.27(19)	10212.14(15)	23.34(4)
O1	3079.5(6)	6841.7(5)	7947.6(4)	16.89(10)

Atom	x	y	z	U_{eq}
O2	2107.3(7)	8627.1(6)	8373.8(5)	27.55(13)
O3	6326.5(6)	2603.1(5)	8350.5(4)	15.62(10)
O4	8728.5(6)	1206.5(5)	8534.5(4)	19.95(11)
O5	6809.7(6)	4905.9(6)	7083.2(5)	20.97(11)
O6	1882.7(6)	5981.7(5)	4716.5(4)	17.64(10)
O7	-472.0(7)	7527.1(6)	4161.1(5)	21.75(11)
O8	3328.2(7)	9462.5(6)	4329.1(5)	23.75(13)
O9	1227.9(7)	10663.5(6)	3198.7(5)	26.33(13)
O10	5874.7(7)	7516.9(6)	6844.6(5)	24.94(13)
C1	2832.4(8)	5046.8(8)	9388.9(5)	16.72(13)
C2	3552.2(7)	5560.7(7)	8496.7(5)	13.69(11)
C3	4757.1(7)	4749.8(7)	8147.8(5)	12.38(11)
C4	5219.7(7)	3427.8(7)	8704.7(5)	13.49(11)
C5	4456.3(8)	2950.3(7)	9580.3(5)	16.22(13)
C6	3258.3(8)	3736.8(8)	9951.9(5)	17.52(13)
C7	3338.9(9)	7555.8(8)	8471.1(7)	22.06(15)
C8	1873.2(12)	9594.8(9)	7368.5(9)	34.8(2)
C9	7772.4(8)	2169.6(7)	8828.0(6)	17.38(13)
C10	8598.0(10)	-0.1(8)	9105.6(7)	24.74(16)
C11	2472.6(9)	3203.4(10)	10903.1(6)	26.33(18)
C12	5479.1(8)	5273.3(7)	7145.6(5)	13.12(11)
C13	4451.8(8)	6180.5(7)	6200.9(5)	13.62(11)
C14	3647.6(8)	5695.1(7)	5846.0(5)	13.36(11)
C15	2658.1(8)	6528.0(7)	4980.7(5)	14.34(12)
C16	2510.8(8)	7792.7(7)	4462.2(6)	16.62(13)
C17	3392.4(8)	8238.6(7)	4798.9(5)	16.46(13)
C18	4360.6(8)	7453.6(7)	5675.2(5)	15.63(12)
C19	3777.0(8)	4323.2(7)	6329.5(5)	14.41(12)
C20	2527.9(8)	4136.1(7)	7040.8(5)	15.94(12)
C21	2469.9(9)	2996.7(8)	7632.1(6)	19.11(14)
C22	1239.3(11)	2858.1(10)	8363.7(7)	29.21(19)
C23	3647.2(11)	1783.3(8)	7614.5(8)	27.39(18)
C24	803.9(9)	6731.2(7)	3881.0(6)	17.32(13)
C25	-1298.3(11)	6855.7(11)	4891.0(8)	30.35(19)
C26	2755.1(10)	10174.5(8)	3281.0(6)	23.40(16)
C27	545.7(13)	11675.2(11)	3559.9(10)	39.6(2)
C28	5203.2(9)	8004.6(8)	6015.2(6)	20.13(14)

Table 15: Anisotropic Displacement Parameters (×10[4]) for BD-6-195. The anisotropic displacement factor exponent takes the form: $-2\pi^2[h^2a^{*2} \times U_{11} + ... + 2hka^* \times b^* \times U_{12}]$

Atom	U_{11}	U_{22}	U_{33}	U_{23}	U_{13}	U_{12}
Cl1	16.36(8)	33.17(11)	25.12(9)	-4.83(7)	6.11(6)	-15.09(8)
Cl2	27.38(9)	16.69(8)	20.65(8)	-10.96(7)	-2.36(6)	1.76(6)
O1	17.5(2)	14.0(2)	15.9(2)	-1.37(19)	-3.11(17)	-5.71(18)
O2	26.0(3)	20.5(3)	33.9(3)	-3.9(2)	2.1(2)	-13.2(2)
O3	16.9(2)	12.6(2)	15.2(2)	-3.45(18)	-1.86(17)	-4.35(18)
O4	18.4(2)	15.1(2)	20.1(3)	-2.5(2)	2.29(19)	-4.6(2)
O5	15.7(2)	22.7(3)	19.2(3)	-6.5(2)	1.63(19)	-3.6(2)
O6	23.4(3)	14.1(2)	15.3(2)	-8.0(2)	-5.13(18)	-2.80(19)
O7	21.7(3)	18.3(3)	24.5(3)	-6.8(2)	-1.6(2)	-7.3(2)
O8	33.3(3)	16.9(3)	20.8(3)	-14.5(2)	-8.4(2)	0.9(2)
O9	30.1(3)	17.2(3)	30.5(3)	-6.8(2)	-9.1(2)	-6.9(2)
O10	31.1(3)	26.2(3)	21.1(3)	-16.5(3)	-6.1(2)	-4.8(2)
C1	13.4(3)	21.7(3)	12.5(3)	-4.7(2)	0.6(2)	-5.8(2)
C2	13.1(3)	14.8(3)	11.3(3)	-3.2(2)	-0.6(2)	-4.7(2)
C3	12.9(3)	11.7(3)	10.6(3)	-3.6(2)	-0.18(19)	-3.2(2)

Atom	U_{11}	U_{22}	U_{33}	U_{23}	U_{13}	U_{12}
C4	13.9(3)	12.1(3)	12.0(3)	-4.2(2)	-1.2(2)	-2.3(2)
C5	16.6(3)	16.4(3)	12.6(3)	-7.0(2)	-1.5(2)	-1.1(2)
C6	15.4(3)	23.0(3)	11.3(3)	-8.2(3)	0.2(2)	-2.7(2)
C7	20.1(3)	20.8(4)	26.0(4)	-3.5(3)	-4.0(3)	-12.1(3)
H7a	27(3)	41(4)	31(4)	-13.6(15)	2.5(14)	-22(2)
H7b	34(5)	31(4)	26(2)	-6(2)	-4.3(11)	-13.0(11)
C8	36.6(5)	17.2(4)	44.5(6)	-8.2(4)	-8.4(4)	-4.9(4)
H8a	48(7)	36(6)	50(5)	-13(3)	-10(3)	-14(2)
H8b	59(5)	31(4)	67(7)	-6.2(19)	-6(3)	-15(2)
H8c	60(4)	89(7)	60(7)	-45(2)	8(2)	-32(3)
C9	15.9(3)	14.7(3)	19.0(3)	-3.4(2)	-1.1(2)	-5.6(3)
H9a	25(6)	10(5)	19(3)	3(4)	-2(2)	-6(2)
H9b	34(6)	23(4)	32(6)	-14(2)	-5(4)	-5(3)
C10	26.4(4)	14.1(3)	27.8(4)	-4.2(3)	0.1(3)	-5.1(3)
H10a	35(4)	36(7)	45(8)	-15(2)	2(3)	-11(5)
H10b	42(6)	37(6)	76(9)	-11(3)	16(4)	-32(3)
H10c	60(8)	35(7)	30(4)	-27(5)	-2(2)	-4(2)
C11	21.6(3)	36.4(5)	14.8(3)	-13.5(3)	2.7(3)	-1.6(3)
H11a	84(9)	137(11)	36(5)	-65(5)	1(3)	-29(3)
H11b	29(3)	68(7)	66(9)	-14.6(19)	6.3(19)	-16(4)
H11c	102(10)	59(4)	66(9)	-49(3)	38(5)	-25(3)
C12	13.9(3)	12.9(3)	11.5(3)	-5.2(2)	0.3(2)	-3.3(2)
C13	15.8(3)	13.8(3)	10.1(3)	-6.4(2)	0.2(2)	-2.4(2)
C14	15.9(3)	12.8(3)	10.4(3)	-5.8(2)	0.0(2)	-3.0(2)
C15	18.4(3)	13.4(3)	10.9(3)	-6.9(2)	-1.3(2)	-2.8(2)
C16	21.3(3)	14.2(3)	13.5(3)	-8.3(3)	-3.6(2)	-1.5(2)
H16	28(7)	30(7)	38(7)	-10(6)	-21(6)	-9(6)
C17	21.5(3)	14.3(3)	13.0(3)	-9.0(3)	-2.2(2)	-1.5(2)
C18	19.0(3)	15.9(3)	12.0(3)	-8.8(2)	-1.4(2)	-2.6(2)
C19	16.6(3)	12.7(3)	12.6(3)	-4.9(2)	0.7(2)	-4.0(2)
H19a	24(4)	25(5)	29(6)	-1(3)	-6(2)	-10(4)
H19b	31(6)	24(6)	24(5)	-9(4)	0(3)	-14(2)
C20	17.9(3)	15.3(3)	13.5(3)	-7.3(2)	1.5(2)	-3.6(2)
H20	32(5)	22(5)	38(7)	-6(3)	9(4)	-10(3)
C21	22.1(3)	19.3(3)	15.8(3)	-11.8(3)	-1.7(2)	-1.8(3)
C22	32.1(4)	37.9(5)	19.9(4)	-24.1(4)	3.9(3)	-3.3(3)
H22a	53(6)	44(5)	28(7)	-19(3)	5(4)	-7(3)
H22b	48(7)	64(7)	51(8)	-41(3)	11(5)	-24(4)
H22c	55(8)	69(8)	28(4)	-34(5)	-4(2)	-4(3)
C23	31.1(4)	15.6(3)	32.1(4)	-9.7(3)	-6.7(3)	-2.4(3)
H23a	126(13)	70(10)	49(5)	-25(7)	6(3)	-24(3)
H23b	56(6)	82(10)	131(12)	-9(4)	-36(4)	-53(7)
H23c	117(10)	48(6)	103(11)	-55(4)	32(6)	-23(4)
C24	21.8(3)	17.2(3)	13.4(3)	-8.2(3)	-2.6(2)	-4.5(2)
H24a	45(6)	35(6)	16(4)	-24(3)	-1(3)	0(3)
H24b	27(6)	26(5)	28(6)	-10(3)	-2(4)	-15(3)
C25	29.2(4)	39.0(5)	30.7(4)	-18.7(4)	8.0(3)	-17.1(4)
H25a	45(6)	47(6)	37(4)	-35(4)	5(3)	-12(3)
H25b	40(5)	60(7)	90(10)	-23(3)	24(3)	-41(4)
H25c	67(8)	62(7)	53(8)	-41(4)	9(5)	-31(3)
C26	30.2(4)	17.5(3)	19.0(3)	-11.5(3)	-4.4(3)	0.8(3)
H26a	46(6)	25(5)	29(5)	-18(3)	2(3)	-8(2)
H26b	54(6)	31(4)	30(6)	-28(2)	-4(4)	-1(3)
C27	39.0(5)	28.5(5)	54.0(7)	-6.7(4)	-6.3(5)	-21.8(5)
H27a	80(9)	34(6)	57(3)	-16(5)	-12.0(19)	-23.7(18)
H27b	71(7)	37(5)	90(9)	-24(3)	3(4)	-27(3)
H27c	44(3)	67(8)	106(10)	-5.7(18)	-11.9(19)	-50(5)
C28	24.7(3)	18.4(3)	18.6(3)	-11.6(3)	-2.3(3)	-4.0(3)
H28	78(9)	35(5)	27(6)	-39(4)	-23(5)	8(3)

Table 16: Bond Lengths in Å for BD-6-195.

Atom	Atom	Length/Å
Cl1	C1	1.7303(8)
Cl2	C5	1.7320(8)
O1	C2	1.3619(9)
O1	C7	1.4516(10)
O2	C7	1.3715(11)
O2	C8	1.4126(12)
O3	C4	1.3669(9)
O3	C9	1.4526(9)
O4	C9	1.3727(10)
O4	C10	1.4301(10)
O5	C12	1.2094(9)
O6	C15	1.3537(9)
O6	C24	1.4268(9)
O7	C24	1.3836(10)
O7	C25	1.4258(11)
O8	C17	1.3637(9)
O8	C26	1.4295(10)
O9	C26	1.3820(11)
O9	C27	1.4231(13)
O10	C28	1.2145(10)
C1	C2	1.4017(10)
C1	C6	1.3964(11)
C2	C3	1.4010(10)
C3	C4	1.4048(10)
C3	C12	1.5106(9)
C4	C5	1.3986(10)
C5	C6	1.3989(11)
C6	C11	1.4994(10)
C7	H7a	0.967(11)
C7	H7b	1.119(12)
C8	H8a	1.0976(19)
C8	H8b	1.0976(19)
C8	H8c	1.0976(19)
C9	H9a	1.093(10)
C9	H9b	1.084(11)
C10	H10a	1.0967(19)
C10	H10b	1.0967(19)
C10	H10c	1.0967(19)

Atom	Atom	Length/Å
C11	H11a	1.0956(19)
C11	H11b	1.0956(19)
C11	H11c	1.0956(19)
C12	C13	1.5170(9)
C13	C14	1.3877(10)
C13	C18	1.4147(10)
C14	C15	1.4136(9)
C14	C19	1.5092(10)
C15	C16	1.3892(10)
C16	H16	1.055(10)
C16	C17	1.3952(10)
C17	C18	1.4073(10)
C18	C28	1.4695(11)
C19	H19a	1.064(11)
C19	H19b	1.0978(19)
C19	C20	1.5116(10)
C20	H20	1.074(12)
C20	C21	1.3372(10)
C21	C22	1.5015(12)
C21	C23	1.5005(13)
C22	H22a	1.080(7)
C22	H22b	1.080(7)
C22	H22c	1.080(7)
C23	H23a	1.046(8)
C23	H23b	1.046(8)
C23	H23c	1.046(8)
C24	H24a	1.084(11)
C24	H24b	1.103(11)
C25	H25a	1.0967(19)
C25	H25b	1.0967(19)
C25	H25c	1.0967(19)
C26	H26a	1.117(13)
C26	H26b	1.111(12)
C27	H27a	1.088(8)
C27	H27b	1.088(8)
C27	H27c	1.088(8)
C28	H28	1.133(12)

Table 17: Bond Angles in ° for BD-6-195.

Atom	Atom	Atom	Angle/°
C7	O1	C2	114.71(6)
C8	O2	C7	113.73(8)
C9	O3	C4	113.96(6)
C10	O4	C9	113.94(6)
C24	O6	C15	119.69(6)
C25	O7	C24	113.00(7)
C26	O8	C17	118.35(6)
C27	O9	C26	113.83(8)
C2	C1	Cl1	118.15(6)
C6	C1	Cl1	119.37(6)
C6	C1	C2	122.47(7)
C1	C2	O1	120.95(7)

Atom	Atom	Atom	Angle/°
C3	C2	O1	119.17(6)
C3	C2	C1	119.87(7)
C4	C3	C2	118.92(6)
C12	C3	C2	120.84(6)
C12	C3	C4	120.08(6)
C3	C4	O3	120.60(6)
C5	C4	O3	119.65(6)
C5	C4	C3	119.52(7)
C4	C5	Cl2	117.91(6)
C6	C5	Cl2	119.27(5)
C6	C5	C4	122.81(7)
C5	C6	C1	116.39(6)
C11	C6	C1	121.75(8)

Atom	Atom	Atom	Angle/˚
C11	C6	C5	121.86(7)
O2	C7	O1	110.36(6)
H7a	C7	O1	101.7(6)
H7a	C7	O2	115.9(7)
H7b	C7	O1	110.8(6)
H7b	C7	O2	106.4(6)
H7b	C7	H7a	111.7(9)
H8a	C8	O2	109.5
H8b	C8	O2	109.5
H8b	C8	H8a	109.5
H8c	C8	O2	109.5
H8c	C8	H8a	109.5
H8c	C8	H8b	109.5
O4	C9	O3	108.13(6)
H9a	C9	O3	107.6(6)
H9a	C9	O4	113.4(5)
H9b	C9	O3	108.5(6)
H9b	C9	O4	108.7(7)
H9b	C9	H9a	110.4(8)
H10a	C10	O4	109.5
H10b	C10	O4	109.5
H10b	C10	H10a	109.5
H10c	C10	O4	109.5
H10c	C10	H10a	109.5
H10c	C10	H10b	109.5
H11a	C11	C6	109.5
H11b	C11	C6	109.5
H11b	C11	H11a	109.5
H11c	C11	C6	109.5
H11c	C11	H11a	109.5
H11c	C11	H11b	109.5
C3	C12	O5	121.48(6)
C13	C12	O5	121.83(6)
C13	C12	C3	116.42(6)
C14	C13	C12	117.19(6)
C18	C13	C12	121.58(6)
C18	C13	C14	121.18(6)
C15	C14	C13	118.30(6)
C19	C14	C13	123.60(6)
C19	C14	C15	118.09(6)
C14	C15	O6	114.03(6)
C16	C15	O6	123.97(6)
C16	C15	C14	122.00(6)
H16	C16	C15	123.3(6)
C17	C16	C15	118.54(6)
C17	C16	H16	118.1(6)
C16	C17	O8	122.39(6)
C18	C17	O8	116.22(6)
C18	C17	C16	121.34(7)
C17	C18	C13	118.48(6)
C28	C18	C13	123.10(6)
C28	C18	C17	118.40(7)

Atom	Atom	Atom	Angle/˚
H19a	C19	C14	111.5(6)
H19b	C19	C14	108.9(6)
H19b	C19	H19a	105.9(9)
C20	C19	C14	112.83(6)
C20	C19	H19a	108.4(6)
C20	C19	H19b	109.2(6)
H20	C20	C19	117.2(6)
C21	C20	C19	123.97(7)
C21	C20	H20	118.8(6)
C22	C21	C20	121.95(8)
C23	C21	C20	121.77(7)
C23	C21	C22	116.28(7)
H22a	C22	C21	109.5
H22b	C22	C21	109.5
H22b	C22	H22a	109.5
H22c	C22	C21	109.5
H22c	C22	H22a	109.5
H22c	C22	H22b	109.5
H23a	C23	C21	109.5
H23b	C23	C21	109.5
H23b	C23	H23a	109.5
H23c	C23	C21	109.5
H23c	C23	H23a	109.5
H23c	C23	H23b	109.5
O7	C24	O6	111.98(6)
H24a	C24	O6	110.2(6)
H24a	C24	O7	106.7(7)
H24b	C24	O6	103.4(6)
H24b	C24	O7	110.3(6)
H24b	C24	H24a	114.4(9)
H25a	C25	O7	109.5
H25b	C25	O7	109.5
H25b	C25	H25a	109.5
H25c	C25	O7	109.5
H25c	C25	H25a	109.5
H25c	C25	H25b	109.5
O9	C26	O8	113.53(7)
H26a	C26	O8	110.0(6)
H26a	C26	O9	107.6(6)
H26b	C26	O8	103.9(6)
H26b	C26	O9	110.8(7)
H26b	C26	H26a	111.0(9)
H27a	C27	O9	109.5
H27b	C27	O9	109.5
H27b	C27	H27a	109.5
H27c	C27	O9	109.5
H27c	C27	H27a	109.5
H27c	C27	H27b	109.5
C18	C28	O10	125.18(7)
H28	C28	O10	119.9(6)
H28	C28	C18	114.9(6)

Table 18: Torsion Angles in ˚ for BD-6-195.

Atom	Atom	Atom	Atom	Angle/˚
Cl1	C1	C2	O1	-0.98(7)
Cl1	C1	C2	C3	-179.66(5)
Cl1	C1	C6	C5	179.10(6)
Cl1	C1	C6	C11	-0.89(7)
Cl2	C5	C4	O3	2.89(6)
Cl2	C5	C4	C3	177.48(5)

Atom	Atom	Atom	Atom	Angle/˚
Cl2	C5	C6	C1	-178.13(6)
Cl2	C5	C6	C11	1.85(7)
O1	C2	C1	C6	177.96(6)
O1	C2	C3	C4	-178.64(6)
O1	C2	C3	C12	-3.26(7)
O1	C7	O2	C8	-73.46(8)
O3	C4	C3	C2	175.65(6)
O3	C4	C3	C12	0.22(7)
O3	C4	C5	C6	-176.31(6)
O3	C9	O4	C10	-79.14(7)
O5	C12	C3	C2	136.69(7)
O5	C12	C3	C4	-47.98(8)
O5	C12	C13	C14	110.21(8)
O5	C12	C13	C18	-67.18(8)
O6	C15	C14	C13	-177.59(6)
O6	C15	C14	C19	2.75(7)
O6	C15	C16	C17	-178.92(8)
O6	C24	O7	C25	-69.44(7)
O8	C17	C16	C15	179.43(8)
O8	C17	C18	C13	179.04(7)
O8	C17	C18	C28	0.46(9)
O8	C26	O9	C27	70.69(9)
O10	C28	C18	C13	-11.72(11)

Atom	Atom	Atom	Atom	Angle/˚
O10	C28	C18	C17	166.80(9)
C1	C2	C3	C4	0.06(8)
C1	C2	C3	C12	175.44(6)
C1	C6	C5	C4	1.06(8)
C2	C3	C4	C5	1.11(7)
C2	C3	C12	C13	-49.20(7)
C3	C4	C5	C6	-1.72(8)
C3	C12	C13	C14	-63.88(7)
C3	C12	C13	C18	118.73(7)
C4	C5	C6	C11	-178.95(7)
C12	C13	C14	C15	178.64(6)
C12	C13	C14	C19	-1.71(8)
C12	C13	C18	C17	179.47(7)
C12	C13	C18	C28	-2.02(8)
C13	C14	C15	C16	2.22(8)
C13	C14	C19	C20	100.78(7)
C13	C18	C17	C16	1.47(9)
C14	C15	C16	C17	1.29(9)
C14	C19	C20	C21	-173.53(6)
C15	C16	C17	C18	-3.16(9)
C16	C17	C18	C28	-177.11(8)
C19	C20	C21	C22	177.11(7)
C19	C20	C21	C23	-1.80(9)

Table 19: Hydrogen Fractional Atomic Coordinates (×10[4]) and equivuivalent Isotropic Displacement Parameters (Å[2]×10[3]) for BD-6-195. U_{eq} is defined as 1/3 of the trace of the orthogonalised U_{ij}.

Atom	x	y	z	U_{eq}
H7a	4208(12)	7701(11)	8133(8)	30(2)
H7b	3556(13)	6980(12)	9324(9)	31(2)
H8a	1566.8(13)	9285.4(11)	6819.8(13)	46(3)
H8b	979(2)	10467.1(18)	7381.1(9)	56(3)
H8c	2892(2)	9784.0(10)	7123.5(10)	64(4)
H9a	7644(11)	1874(10)	9669(8)	21(2)
H9b	8156(13)	2955(11)	8534(9)	30(3)
H10a	7481(2)	70.3(8)	8977.7(7)	39(3)
H10b	9413.6(17)	-720.0(15)	8840.0(8)	51(4)
H10c	8798.8(11)	-275.8(9)	9936.9(16)	41(3)
H11a	2951.5(13)	3169.5(10)	11585.2(13)	79(5)
H11b	1296(2)	3811.7(14)	10810.9(6)	59(4)
H11c	2596.1(10)	2239(2)	11008.1(6)	73(4)
H16	1745(13)	8443(11)	3837(9)	31(3)
H19a	4796(12)	3721(11)	6758(9)	28(3)
H19b	3789(13)	3991(10)	5707(6)	25(3)
H20	1651(13)	4976(12)	7077(10)	33(3)
H22a	415(6)	3789(7)	8257.3(10)	44(3)
H22b	741(4)	2302(4)	8204.7(13)	47(4)
H22c	1677(3)	2384(3)	9155(6)	51(4)
H23a	3835.5(19)	1855.7(10)	6845(6)	85(5)
H23b	4625(8)	1625.7(15)	7963(3)	87(5)
H23c	3302(3)	1023(6)	8023(3)	86(5)
H24a	1232(13)	7339(12)	3231(8)	32(3)
H24b	571(12)	6013(11)	3718(9)	25(2)
H25a	-671.6(15)	6283.8(15)	5635.4(15)	38(3)
H25b	-2359(2)	7539.3(16)	5004.3(8)	60(4)
H25c	-1499.9(12)	6227.7(15)	4594.8(9)	53(4)
H26a	3174(14)	9558(12)	2819(10)	33(3)
H26b	3190(15)	10954(12)	2993(9)	37(3)
H27a	776(2)	11317(3)	4391(6)	55(3)
H27b	983(3)	12421(6)	3154(3)	64(4)
H27c	-644(9)	12061(3)	3410.1(15)	70(4)
H28	5188(16)	8965(13)	5438(10)	45(4)

Citations

CrysAlisPro (ROD), Rigaku Oxford Diffraction, Poland (?).

CrysAlisPro Software System, Rigaku Oxford Diffraction, (2024).

L.J. Bourhis and O.V. Dolomanov and R.J. Gildea and J.A.K. Howard and H. Puschmann, The Anatomy of a Comprehensive Constrained, Restrained, Refinement Program for the Modern Computing Environment - Olex2 Disected, *Acta Cryst. A*, (2015), **A71**, 59-71.

O.V. Dolomanov and L.J. Bourhis and R.J. Gildea and J.A.K. Howard and H. Puschmann, Olex2: A complete structure solution, refinement and analysis program, *J. Appl. Cryst.*, (2009), **42**, 339-341.

Sheldrick, G.M., ShelXT-Integrated space-group and crystal-structure determination, *Acta Cryst.*, (2015), **A71**, 3-8.
